# Supplementary material for: Equilibrium and thermodynamic studies of chromic overcrowded fluorenylidene-acridanes with modified fluorene moieties
Source: Commun Chem. 2020 Jul 24;3:93. doi: 10.1038/s42004-020-00345-6 (PMC9814365; doi:10.1038/s42004-020-00345-6)
Supplement: Supplementary file 2 — Supplementary Information [file 42004_2020_345_MOESM2_ESM.pdf]

Supplementary Information

Equilibrium and Thermodynamic Studies of Chromic Overcrowded Fluorenylidene-Acridanes with Modified Fluorene Moieties

Ya Wang[1], Yue Ma[1], Keisuke Ogumi[2,3], Bing Wang[1], Takafumi Nakagawa[4], Yao Fu[1], Yutaka Matsuo*[1,2,4,5]

[1] *Department of Chemistry, School of Chemistry and Materials Science, and Hefei National Laboratory for Physical Sciences at the Microscale, University of Science and Technology of China, 96 Jinzhai Road, Hefei, Anhui 230026, China; E-mail: matsuo@ustc.edu.cn*

[2] *Department of Chemical System Engineering, Graduate School of Engineering, Nagoya University, Furo-cho, Chikusa-ku, Nagoya 464-8603, Japan; E-mail: yutaka.matsuo@chem.mater.nagoya-u.ac.jp*

[3] *Tokyo Metropolitan Industrial Technology Research Institute, 2-4-10 Aomi, Koto-ku, Tokyo 135-0064, Japan*

[4] *Department of Mechanical Engineering, School of Engineering, The University of Tokyo, 7-3-1 Hongo, Bunkyo-ku, Tokyo 113-8656, Japan*

[5] *Institute of Materials Innovation, Institutes of Innovation for Future Society, Nagoya University, Furo-cho, Chikusa-ku, Nagoya 464-8603, Japan*

Table of Contents:

1. Supplementary Methods ······ 2

2. ^{1}H NMR, ^{13}C NMR, ^{19}F NMR, and HRMS Charts ······ 6

3. Thermal gravimetric Analysis (TGA) and differential scanning calorimetry (DSC) data ······ 26

4. Light Absorption Properties ······ 27

5. Fluorescence Properties ······ 29

6. X-ray Structure and Structure Analysis ······ 30

7. Crystal Data Collection Parameters ······ 34

8. Various Chromic Behavior ······ 53

9. Cyclic Voltammograms ······ 56

10. DFT Studies ······ 57

11. Charge Carrier Mobility Data ······ 62

12. Exaplanation of Equilibrium Study Based on Variable Temperature UV-Vis ······ 64

13. Melting and Protonation/Deprotonation ······ 66

14. Piezofluorochromism ······ 67

15. Powder XRD ······ 68

16. Supplementary References ······ 69

1. Supplementary Methods

1.1. Synthesis of the Starting Materials 1, 2, 3, 4, and 5

General remarks. All reagents commercially available were used as received without further purification. ^{1}H and ^{13}C NMR spectra were respectively recorded at 500.16 MHz and 125.77 MHz on a JEOL ECZ-500 system. High-resolution (HR) mass spectra were obtained by MALDI using a time-of-flight mass analyzer on a Bruker Ultra exTOF/TOF spectrometer. The UV–visible spectrum was recorded on a JASCO V-570 spectrometer. CV measurements were performed with a HOKUTO DENKO HZ-5000 voltammetric analyzer.

Procedures for synthesis of 1a and 1b

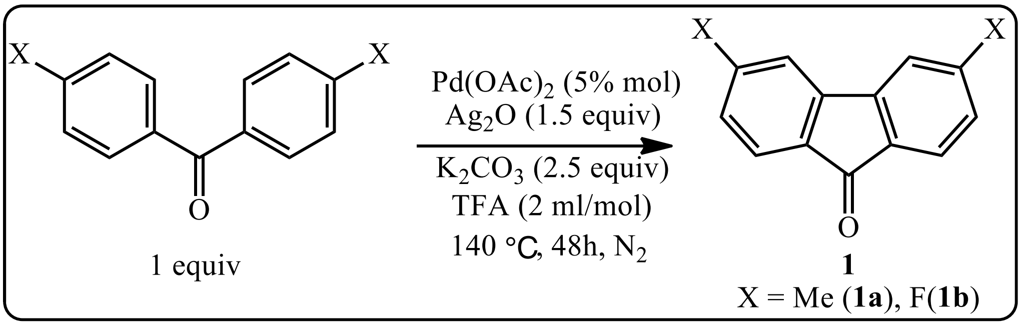

Supplementary Figure 1. Synthesis of **1a** and **1b** by Pd-catalyzed double C−H bond activations reaction.

Supplementary Note 1

1a: Under air atmosphere, $Pd(OAc)_2$ (2.2 mg, 0.01 mmol), Ag_2CO_3 (69.5 mg, 0.30 mmol), K_2CO_3 (69.1 mg, 0.50 mmol) and the benzophenone derivatives (42.0 mg, 0.20 mmol) were added into a Schlenk tube dried by hot-gun. The tube was stopped and degassed with N_2 for three times. Then trifluoroacetic acid (TFA) (0.50 mL) was added by syringe. The mixture was stirred under N_2 atmosphere at 140 ℃ for 48 h. Then the mixture was cooled down to room temperature and evaporated in vacuum and further purified by flash chromatography on silica gel with petroleum ether/ethyl acetate (15:1) to give the product **1a** (35.7 mg, 82%) as a yellow solid.
1b: Under air atmosphere, $Pd(OAc)_2$ (2.2 mg, 0.01 mmol), Ag_2CO_3 (69.5 mg, 0.30 mmol), K_2CO_3 (69.1 mg, 0.50 mmol,) and the benzophenone derivatives (43.6 mg, 0.20 mmol) were added into a Schlenk tube dried by hot-gun. The tube was stopped and degassed with N_2 for three times. Then trifluoroacetic acid (TFA) (0.50 mL) was added by syringe. The mixture was stirred under N_2 atmosphere at 140 ℃ for 48 h. Then the mixture was cooled down to room temperature and evaporated in vacuum and further purified by flash chromatography on silica gel with petroleum ether/ethyl acetate (15:1) to give the product **1b** (37.6mg, 87%) as a yellow solid[1].

Procedures for synthesis of 1c

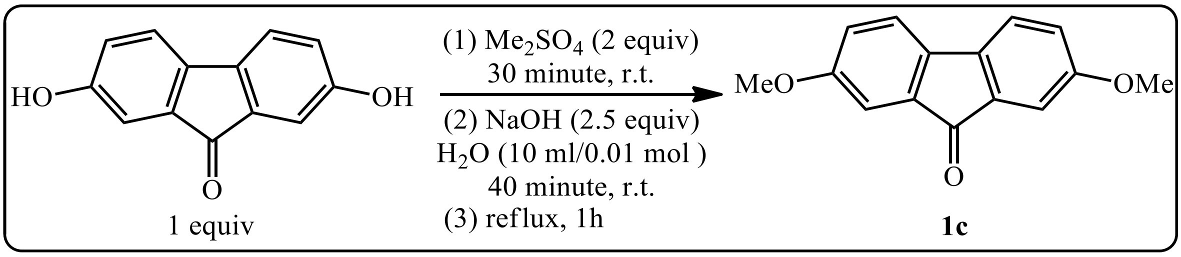

Supplementary Figure 2. Synthesis of **1c** by alkylation reaction. 2.12 g (0.01 mol) of bisphenol was added to a solution of 1 g of sodium hydroxide in 10 ml of water. The mixture was stirred for 30 min. 2.52 g (0.02 mmol) of dimethyl sulfate was added drop-wisely over a period of 40 min. The mixture was stirred for 1 h,

maintaining it slightly boiling, and cooled. The precipitate was filtered off and washed with water. The product (**1c**) was purified by recrystallization from ethanol[2].

Preparation of 2,7-di(thiophen-2-yl)-fluoren-9-one (1e) from 1d

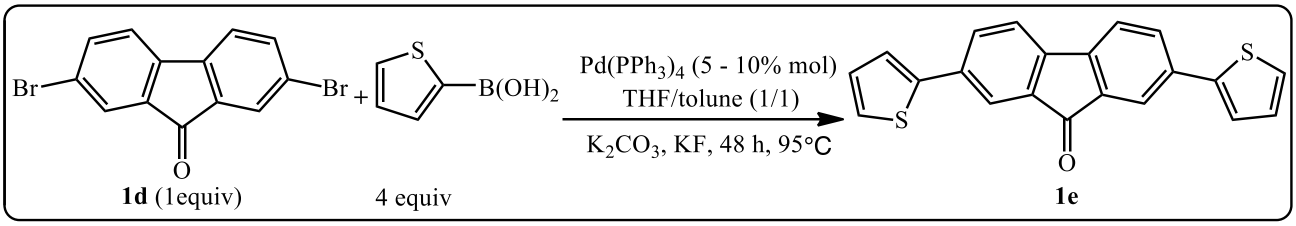

Supplementary Figure 3. Synthesis of **1e** by the Suzuki reaction. 2,7-dibromo-fluoren-9-one (**1d**, commercial available, 3.0 g, 9 mmol), thiophen-2-ylboronic acid (4.6 g, 36 mmol), $Pd(PPh_3)_4$ (1.0 g, 5-10% mmol), toluene/THF (54 mL), K_2CO_3/ KF (1.24 g, 9 mmol; 0.52 g, 9 mmol; 45 mmol). The production was purified by column chromatography (petroleum ether/dichloromethane, 3:1). To recrystallize it from petroleum ether containing a small amount of dichloromethane for three times give product **1e** (1.2 g, 39%) as a red solid[3].

Synthesis of 2,7-Nitro-9-fluorenone (1f)

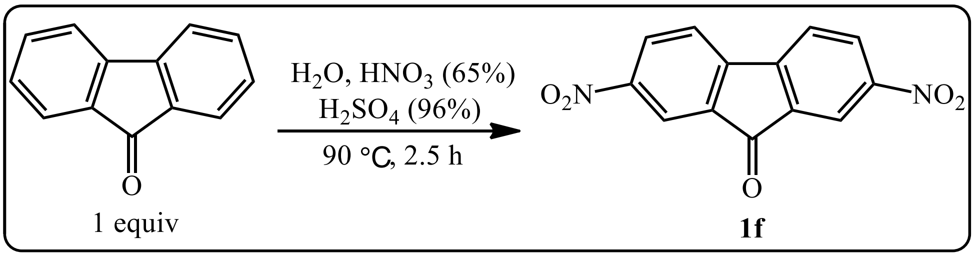

Supplementary Figure 4. Synthesis of **1f** by nitration. A mixture of 9-fluorenone (9.0 g, 50 mmol) and water (10 mL) was heated to 80 ℃. A mixture of HNO_3 (65%, 144 mmol) and H_2SO_4 (96%, 180 mmol) was then added dropwise. After being stirred at 90 ℃ for 2.5 h, the reaction mixture was quenched with water (200 mL). The crude product was filtered, washed with water (3×100 mL), and dried. The compound was purified by recrystallization from ethanol to give 10.35 g of **1f** as a yellow solid, yield 92%[4].

Synthesis of 9-fluorenehydrazone derivatives (2a-f)

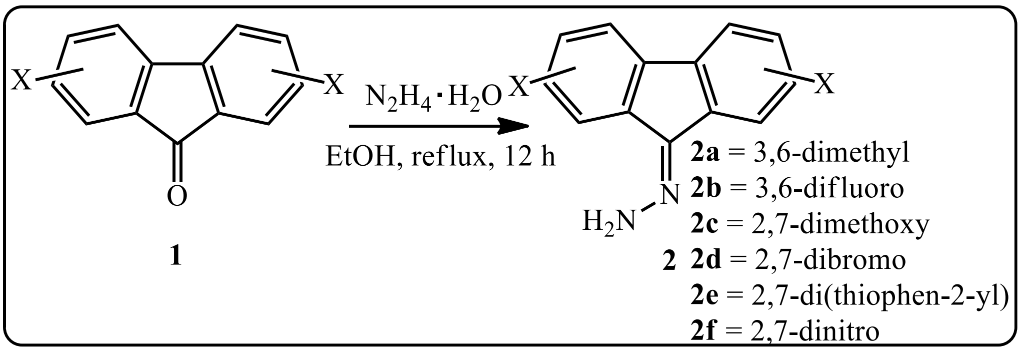

Supplementary Figure 5. Synthesis of Schiff base by aldimine condensation. Fluorene (20.0 mmol) and hydrazine monohydrate (8.0 mL, **ca.** 8 equiv.) were dissolved in ethanol (200 mL, 0.10 M). The reaction

mixture was refluxed and stirred for 12 h. After cooling the solution to room temperature, solvent was evaporated to obtain solids (~ 96%), which was pure enough to use for next reaction[5].

Synthesis of 9-diazofluorene derivatives (3a-f)

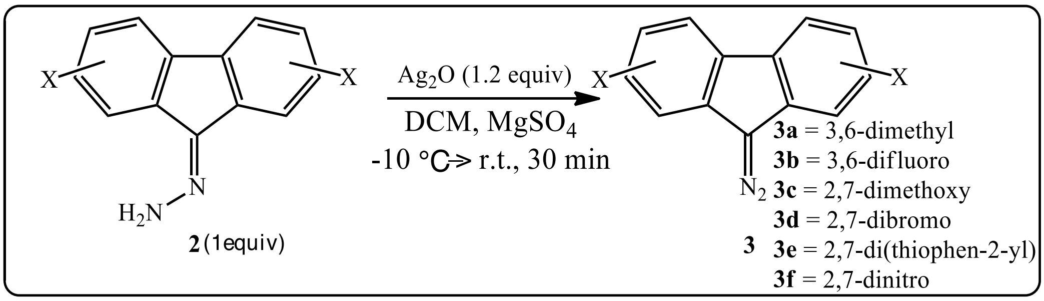

Supplementary Figure 6. Synthesis of 9-diazofluorene derivatives by oxidation of fluorenehydrazone. 9-Fluorenehydrazone derivatives (20.0 mmol) and excess amount of magnesium sulfate (2.86 g, 22.3 mmol) were put in a 500 mL two-neck flask. Dichloromethane (200 mL, 0.10 M) was added to the flask and cooled to 0 ºC with an ice bath. After silver oxide was added and stirred for 5 min, it was warmed up to room temperature and stirred for 30 min. The filtrate was dried to use for next reaction without further purification. The product gave the identical ^{1}H NMR chart to the literature[6].

Procedures for the synthesis of *N*-phenyl thioacridone (4)

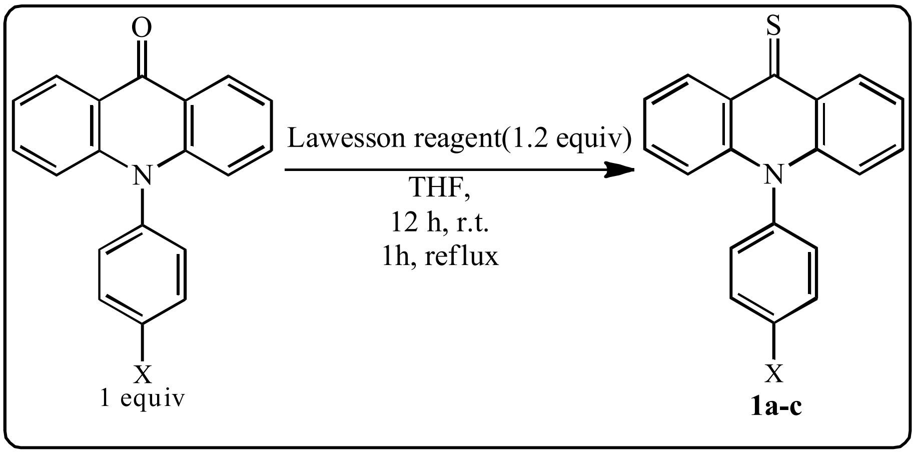

Supplementary Figure 7. Synthesis of thioacridone with Lawesson reagent. In a 300 mL two-neck flask, 10-arylacridin-9(10H)-one (50 mmol, 1 eq.) and Lawesson's reagent (60 mol, 1.2 eq.) were stirred in THF for 12 h at room temperature, and then it was heated up to reflux for 1 h. After drying, sticky brown crude product was obtained to purify silica gel column chromatography with dichloromethane. The resulted brown solids were reprecipitated from dichloromethane/methanol to obtain the target compound[7].

Synthesis of 9-(9H-fluoren-9-ylidene)-10-phenyl-9,10-dihydroacridine derivatives (5a-f)

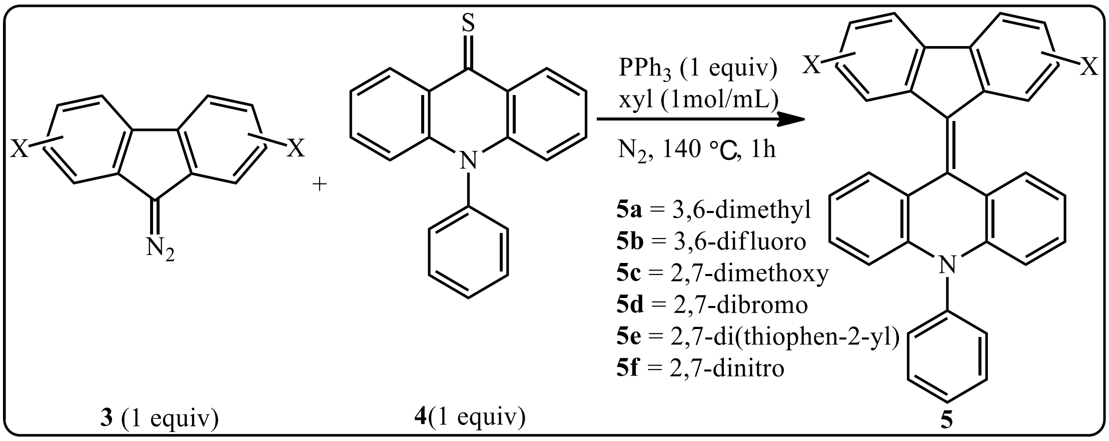

Supplementary Figure 8. Synthesis of Ph-FA derivatives by the Barton–Kellogg reaction (one pot method).

Supplementary Note 2

Into a Schlenk bottle, 10-phenylacridine-9(10H)-thione (**4**, 1 equiv) and triphenyl phosphine (1 equiv) are added, and then put the rubber cover on. The reaction system is designed to keep normal N_2 pressure by using a Schlenk line. To start the reaction, anhydrous xylene 4 mL/mmol was injected into the bottle, and diazofluorene **3** (1 equiv) was added drop wisely during the reaction. The mixture was refluxed totally for 1 h. After removing the solvent by reduced pressure distillation, the mixture was charged on a silica gel short column, and then dichloromethane was passed through the column to remove some impurities. Then, a triethylamine/dichloromethane mixture (1/1 to 1/5) was passed to desorb the product from silica gel, collecting the dark green or blue compounds. Second stage silica gel column chromatography, which was prior treated with triethylamine, was performed using petroleum ether/dichloromethane (10/1) eluent to obtain the target compounds **5**, yield 35-70%[8].

1.2. DFT Studies

All calculations on this section were carried out using Gaussian 16[9]. Geometry optimizations were performed using the B3LYP functional[10–12] with 6-31G(d) basis set. Frequency analyses were conducted at the same level of theory to obtain the thermal correction and confirm the stationary points to be minima. For single-point energy calculations, the B3LYP functional was applied with the 6-311+G(d,p) basis set. The simulated UV–Vis spectra was obtained based on the time-dependent density functional theory. The vertical excitation from the ground state was calculated at TD-B3LYP/6-311+G(d,p) level. Grimme's empirical dispersion-corrections[13] (known as Grimme-D3) and solvation effects of DMF solvent (using SMD model[14]) were introduced to all above calculations, including the optimization and frequency analysis.

2. ^{1}H NMR, ^{13}C NMR, ^{19}F NMR, and HRMS Charts

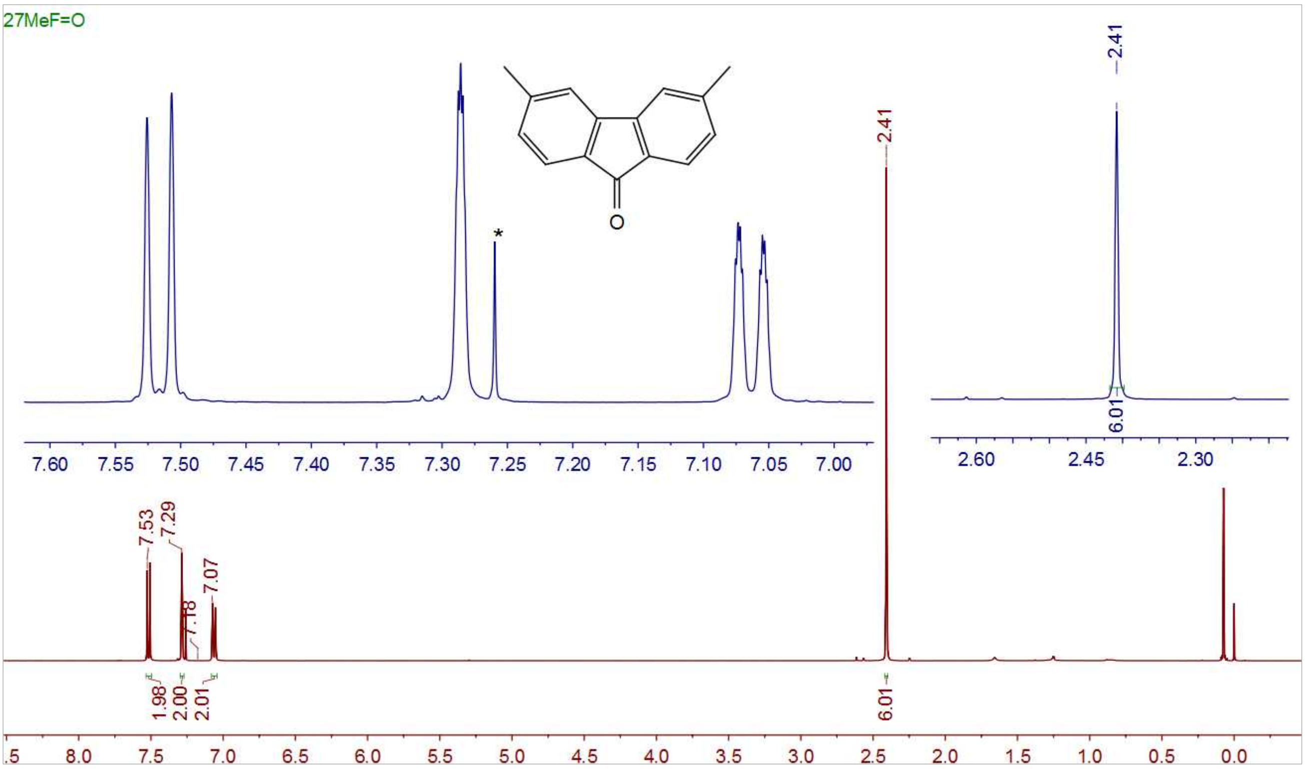

Supplementary Figure 9. ^{1}H NMR (400 MHz) of **1a** in $CDCl_3$.

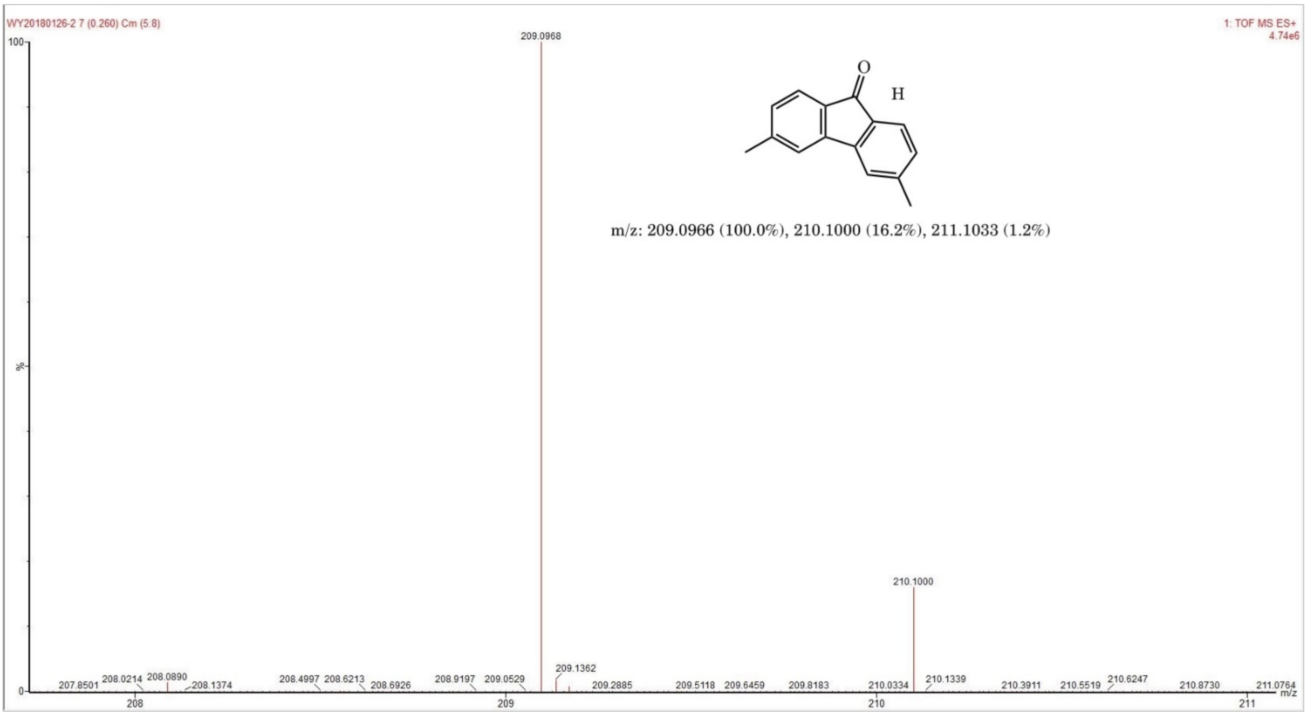

Supplementary Figure 10. HR ESI MS of **1a**. m/z = 209.0968.

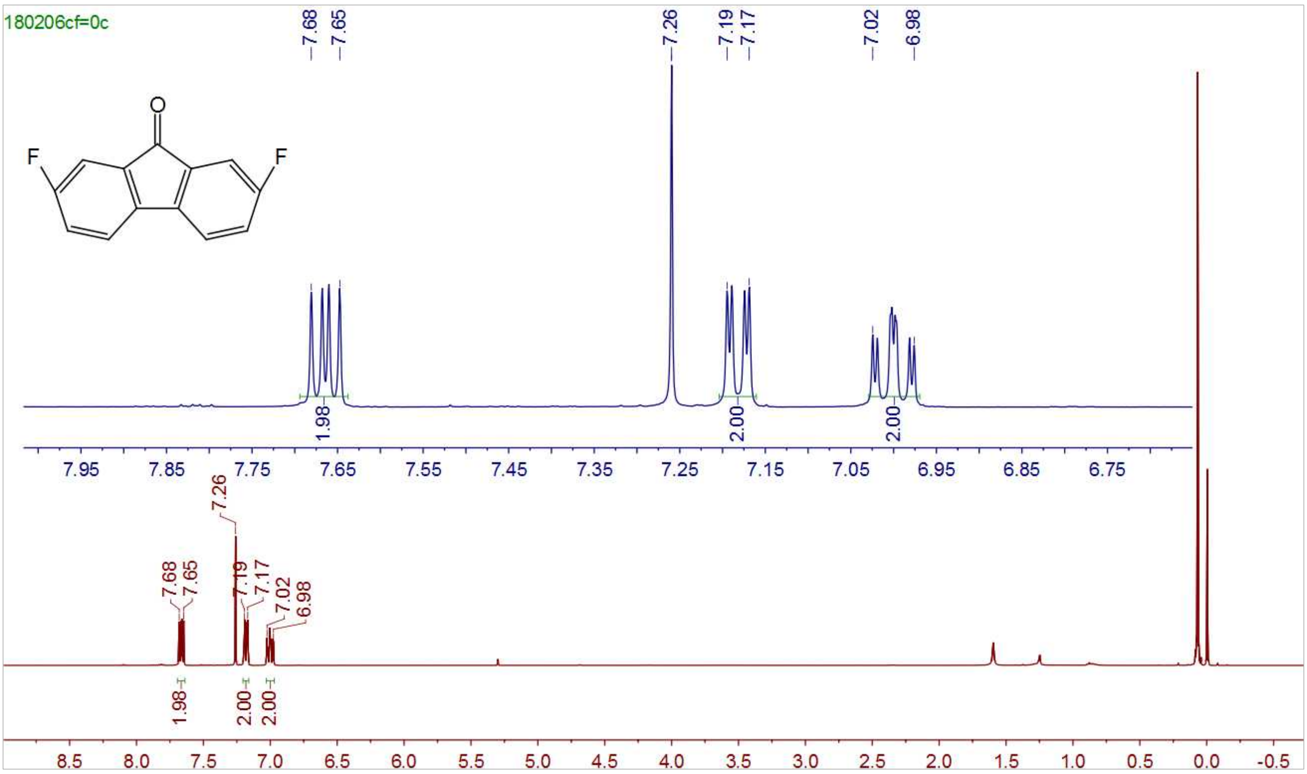

Supplementary Figure 11. ^{1}H NMR (400 MHz) of **1b** in $CDCl_3$.

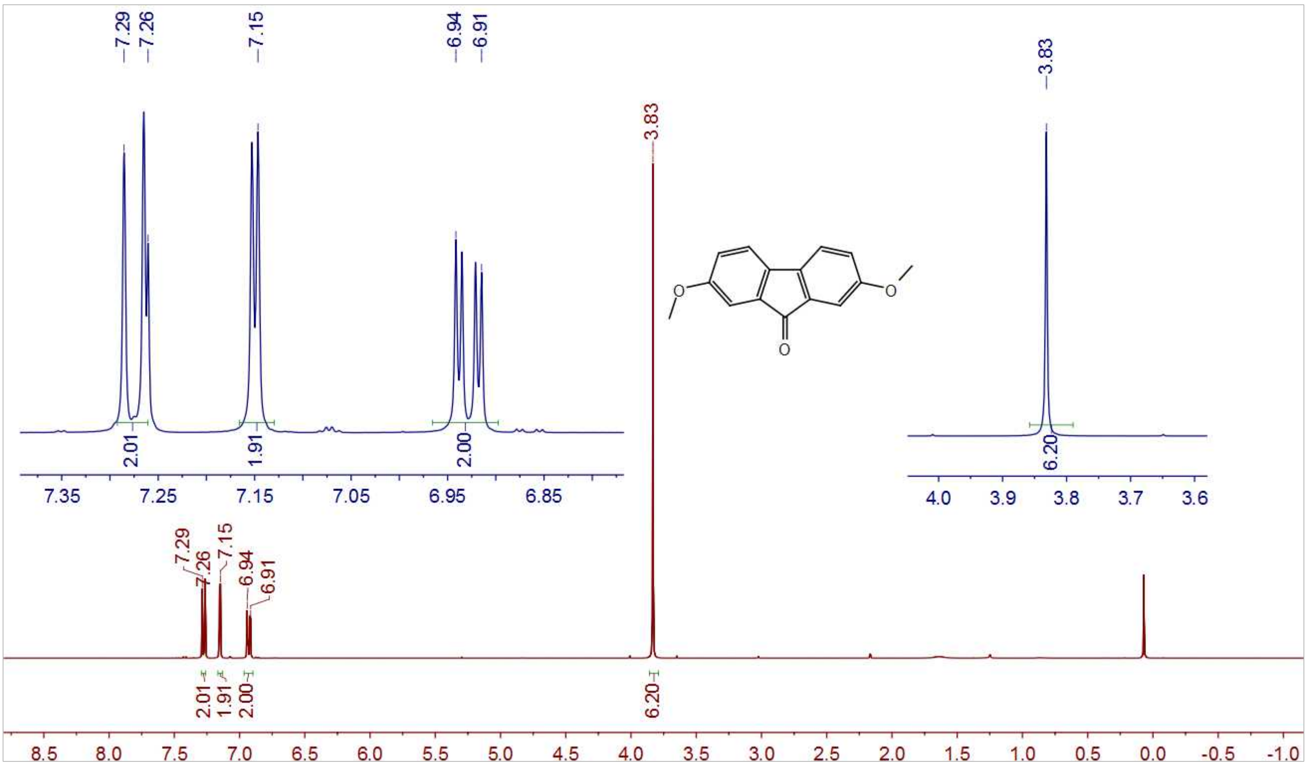

Supplementary Figure 12. ^{1}H NMR (400 MHz) of **1c** in $CDCl_3$.

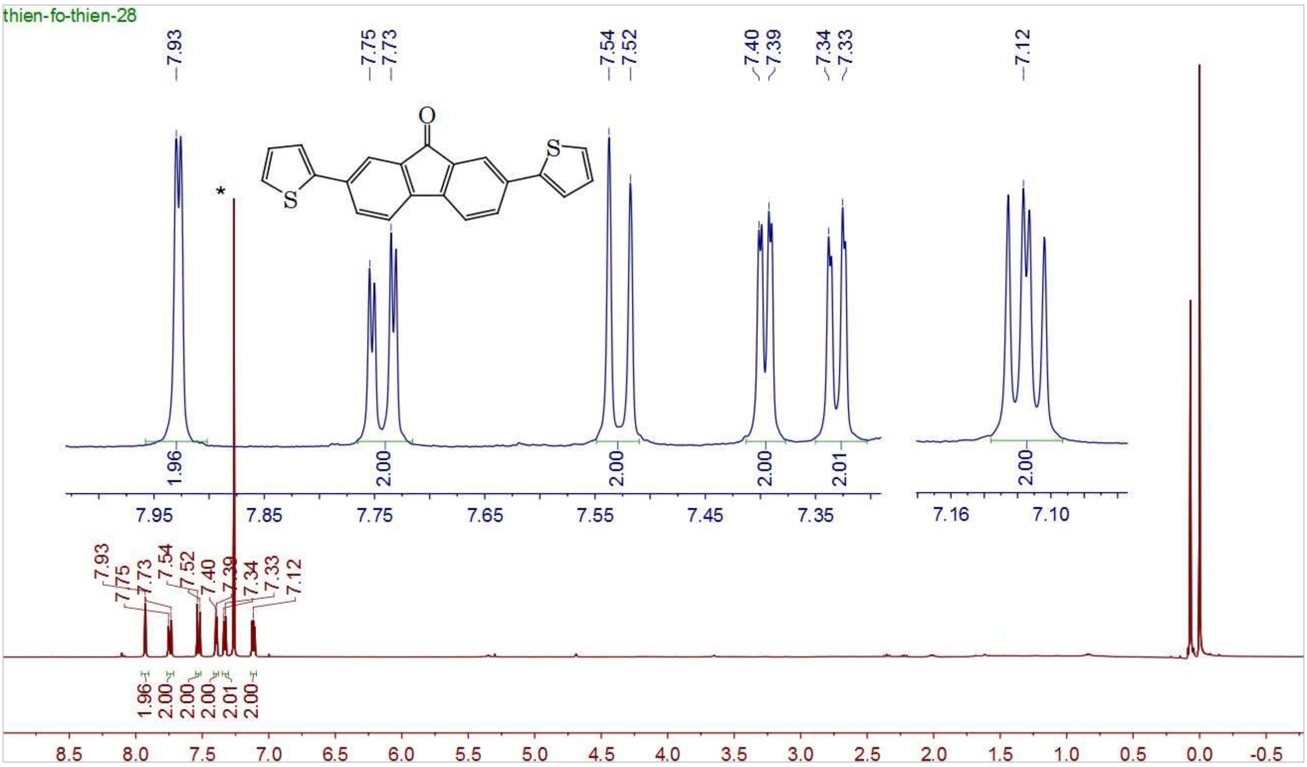

Supplementary Figure 13. ^{1}H NMR (400 MHz) of **1e** in $CDCl_3$.

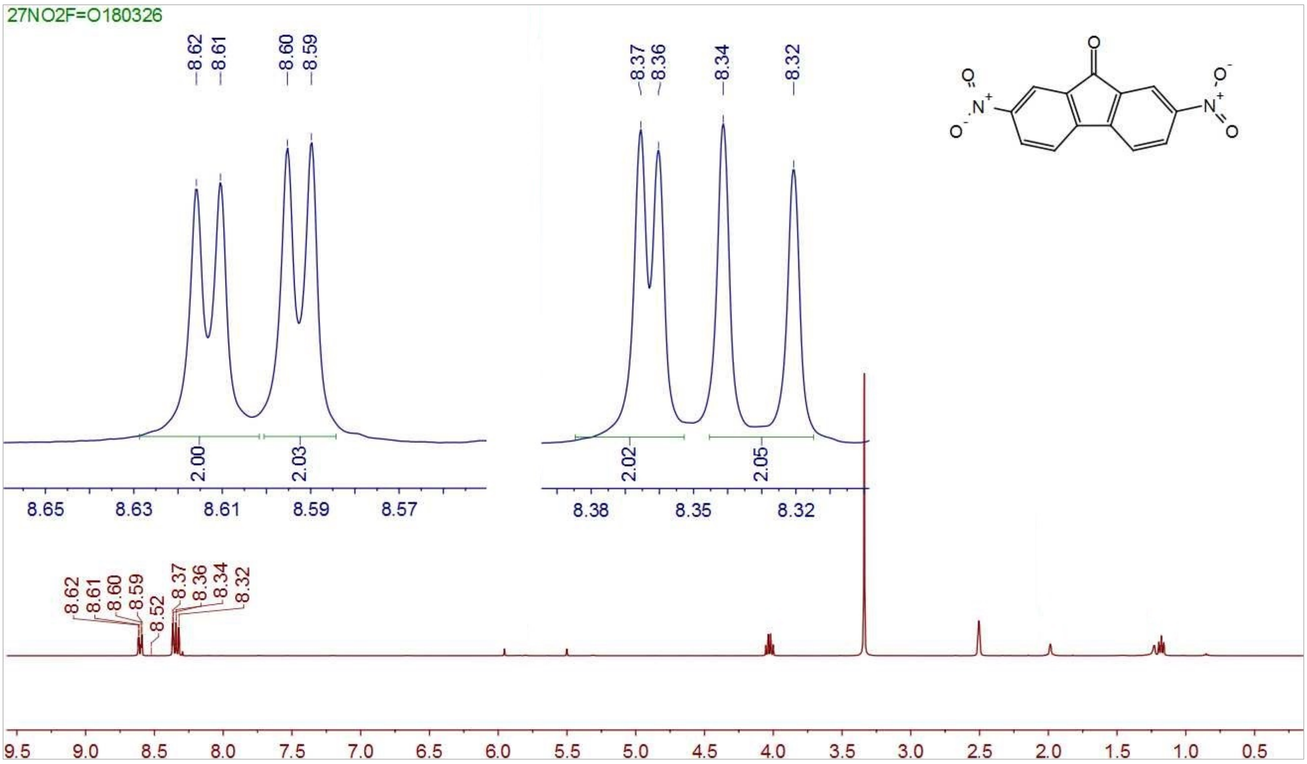

Supplementary Figure 14. ^{1}H NMR (400 MHz) of **1f** in DMSO-d_6.

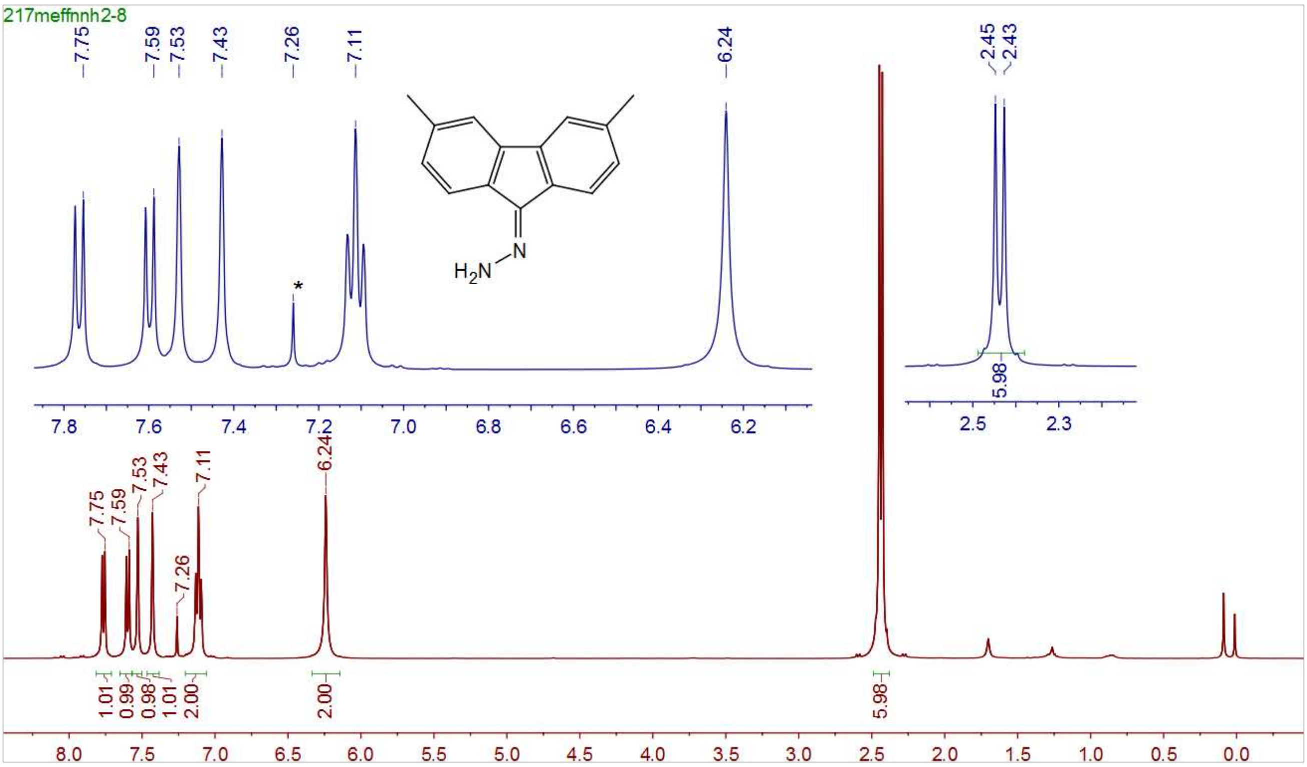

Supplementary Figure 15. ^{1}H NMR (400 MHz) of **2a** in $CDCl_3$.

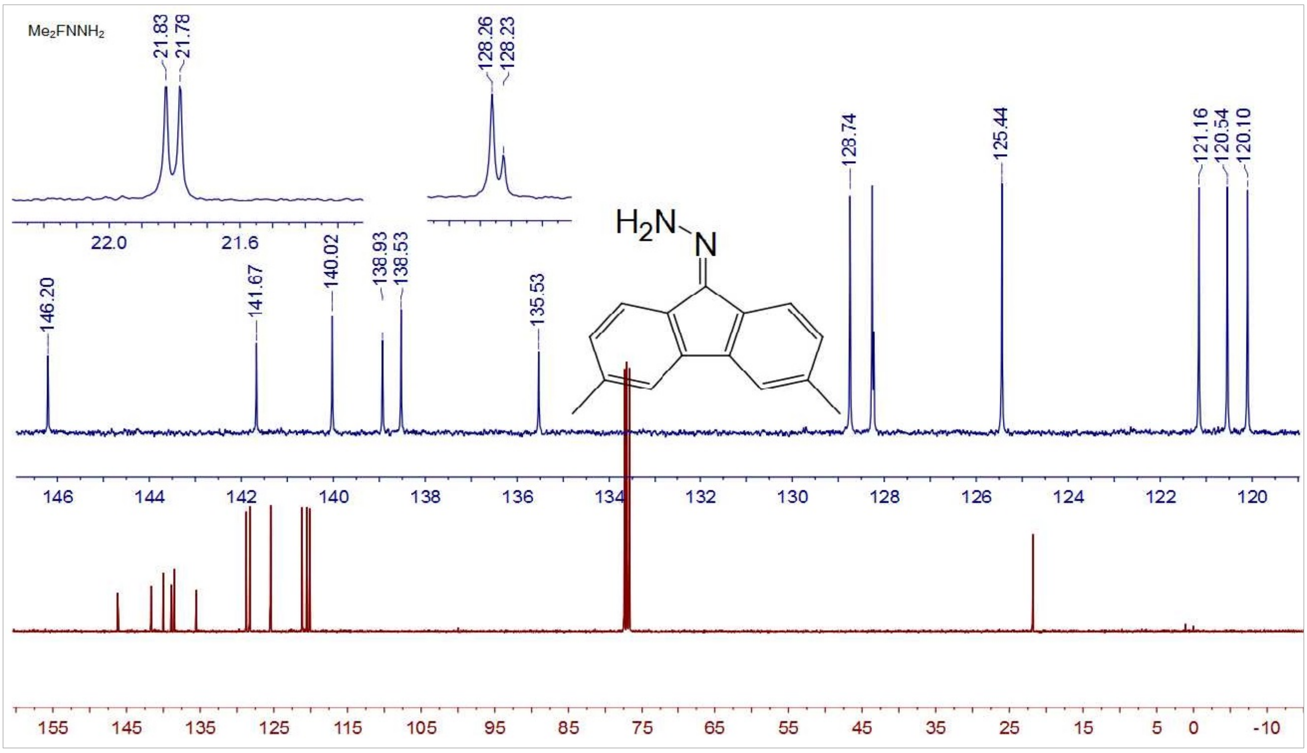

Supplementary Figure 16. ^{13}C NMR (100 MHz) of **2a** in $CDCl_3$.

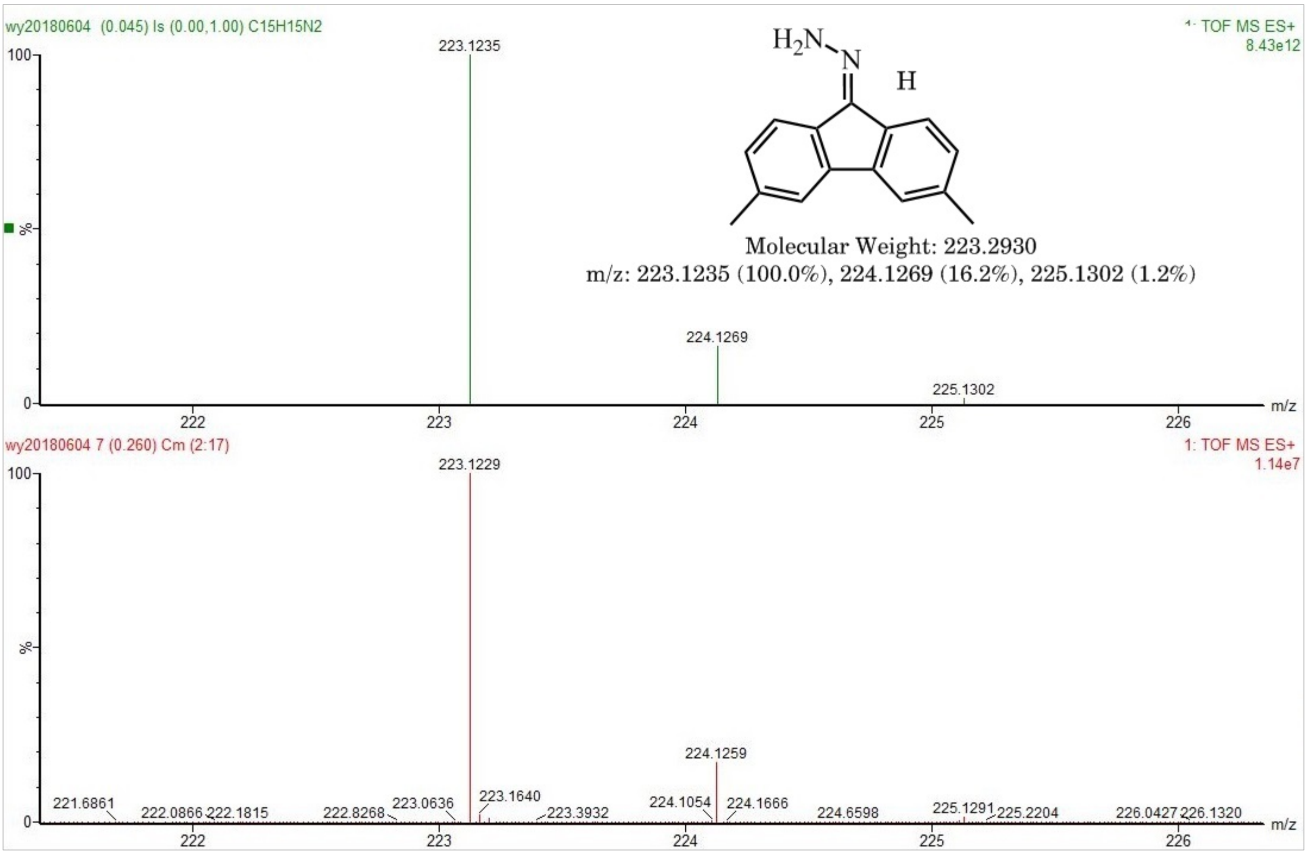

Supplementary Figure 17. HR ESI MS of **2a**. *m/z* = 223.1229.

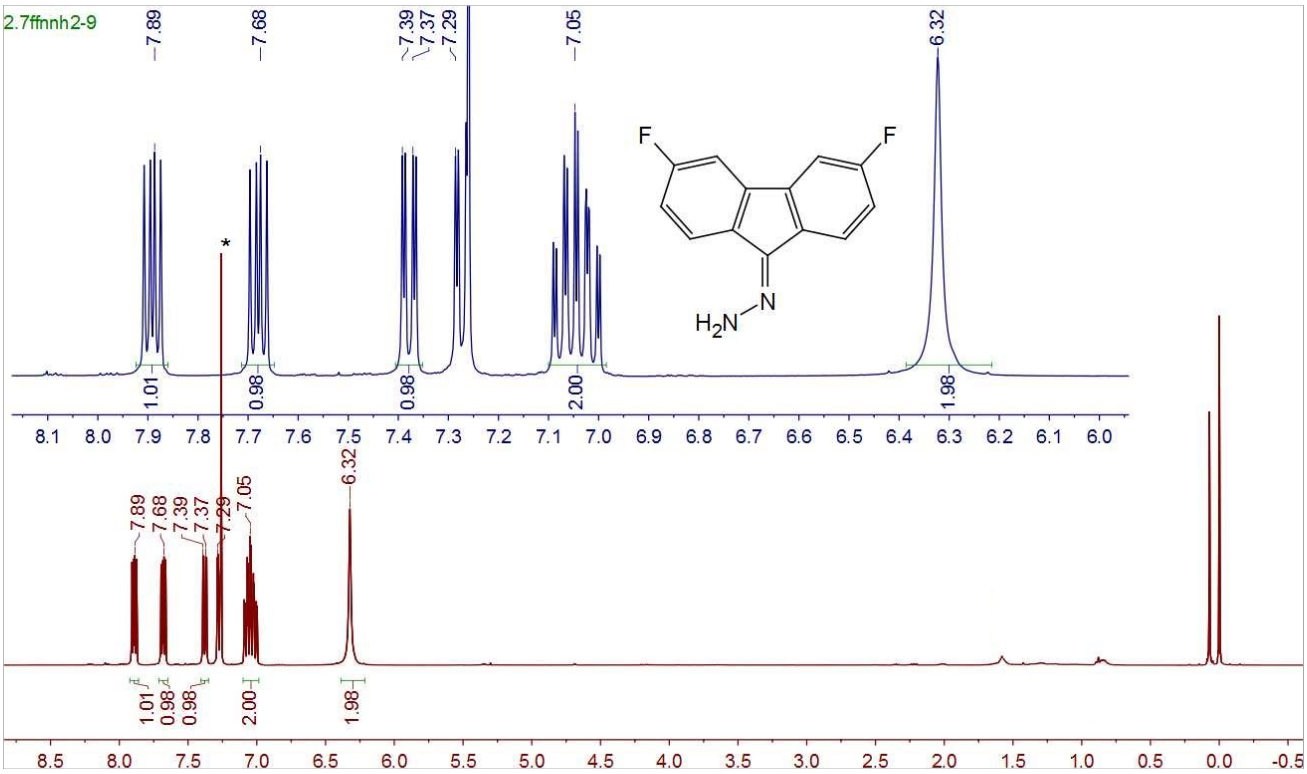

Supplementary Figure 18. ^{1}H NMR (400 MHz) of **2b** in $CDCl_3$.

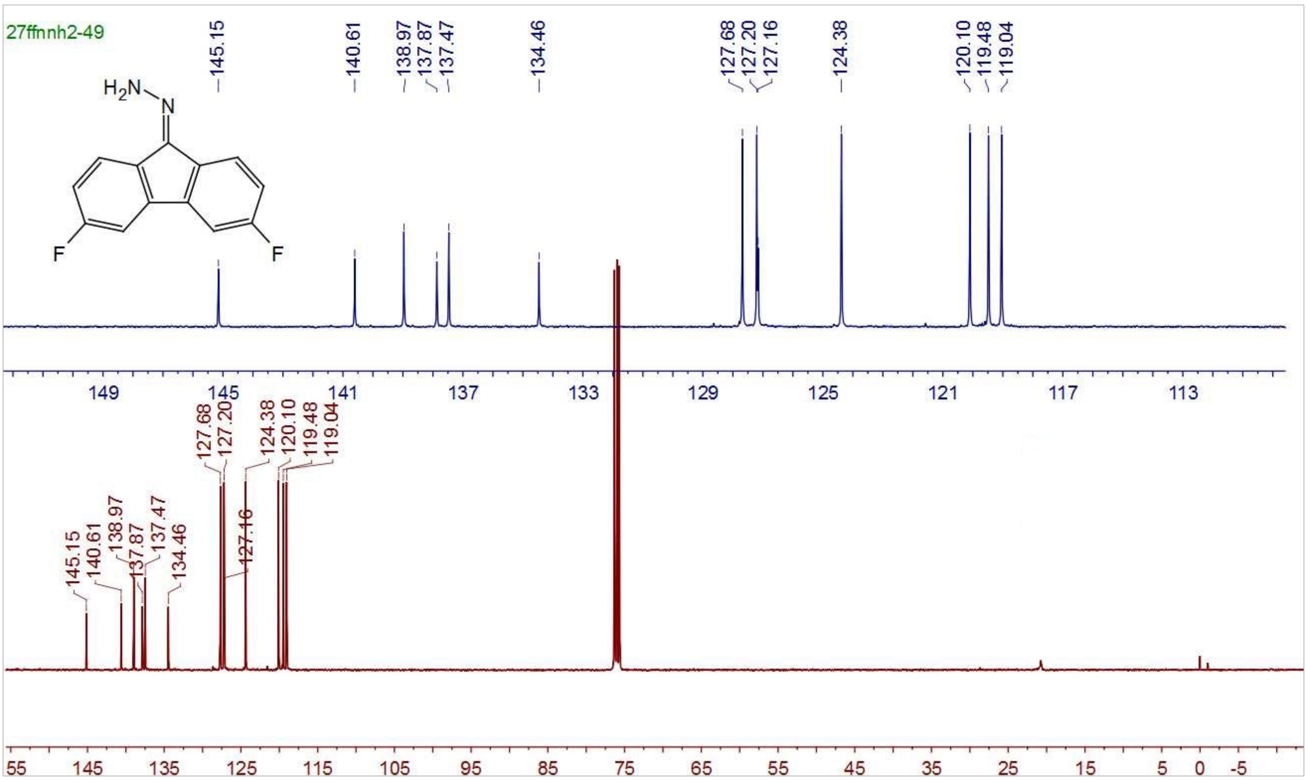

Supplementary Figure 19. ^{13}C NMR (100 MHz) of **2b** in $CDCl_3$.

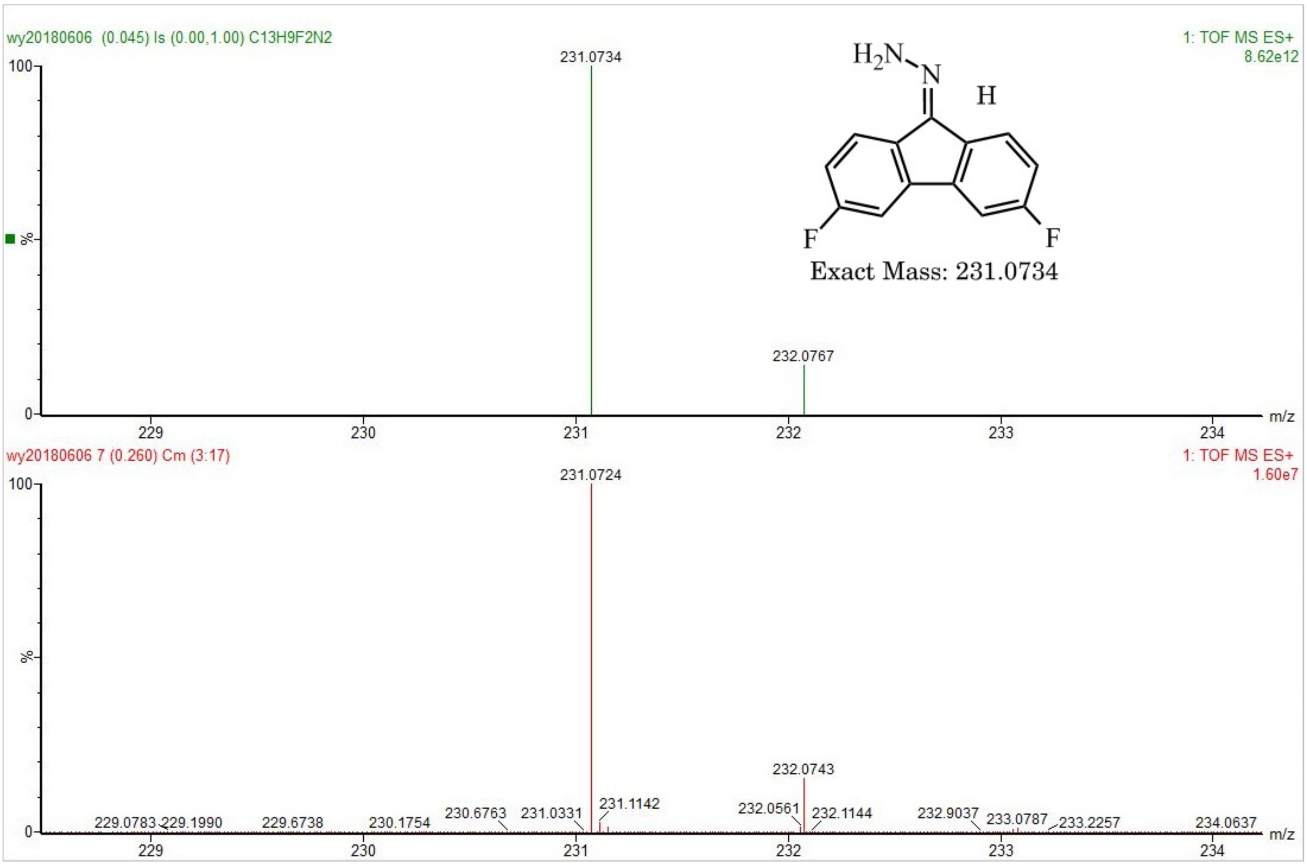

Supplementary Figure 20. HR ESI MS of **2b**. m/z = 231.0724.

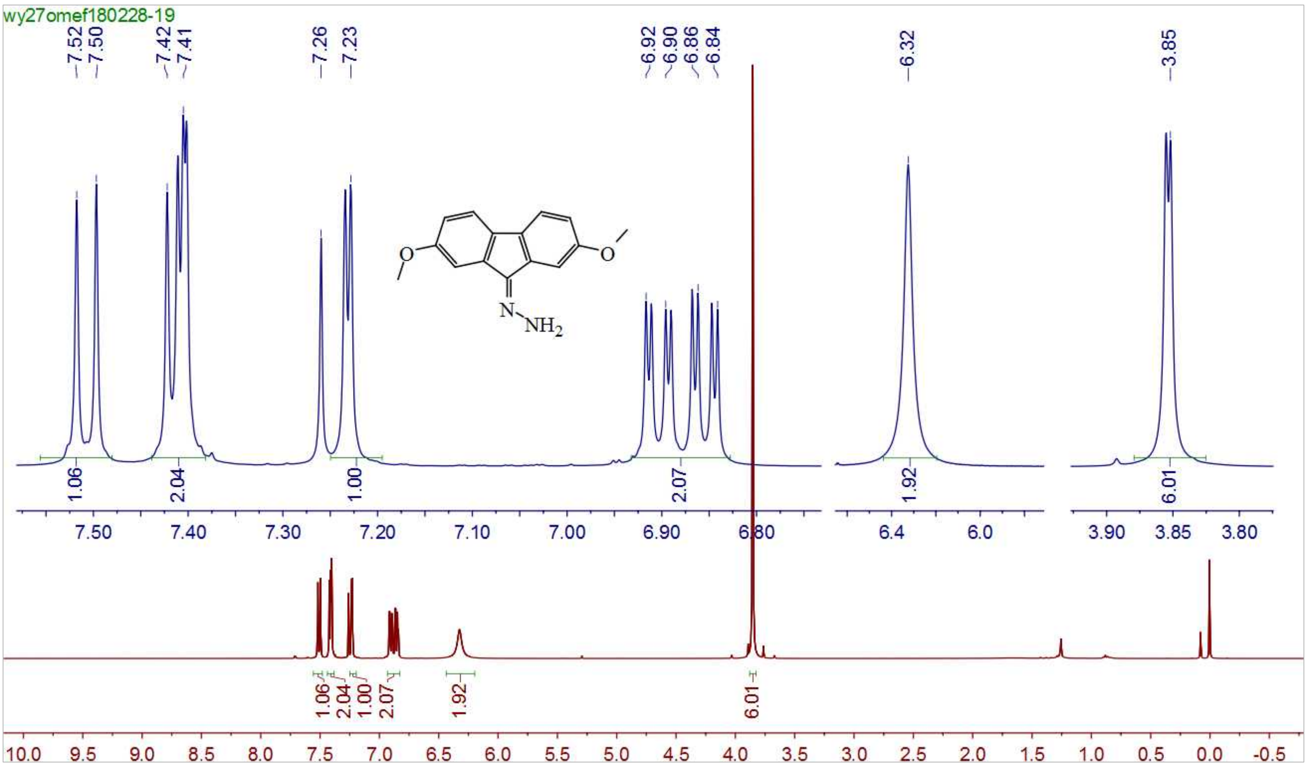

Supplementary Figure 21. ^{1}H NMR (400 MHz) of **2c** in $CDCl_3$.

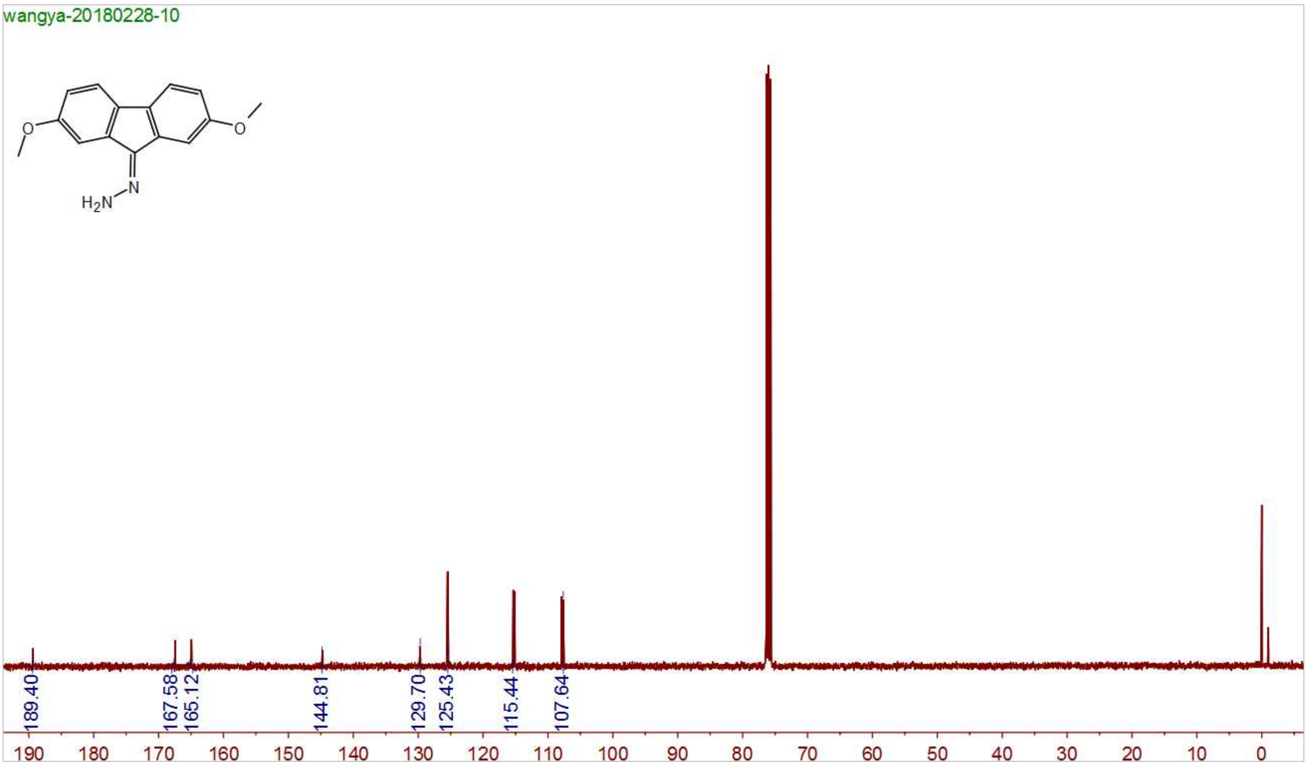

Supplementary Figure 22. ^{13}C NMR (100 MHz) of **2c** in $CDCl_3$.

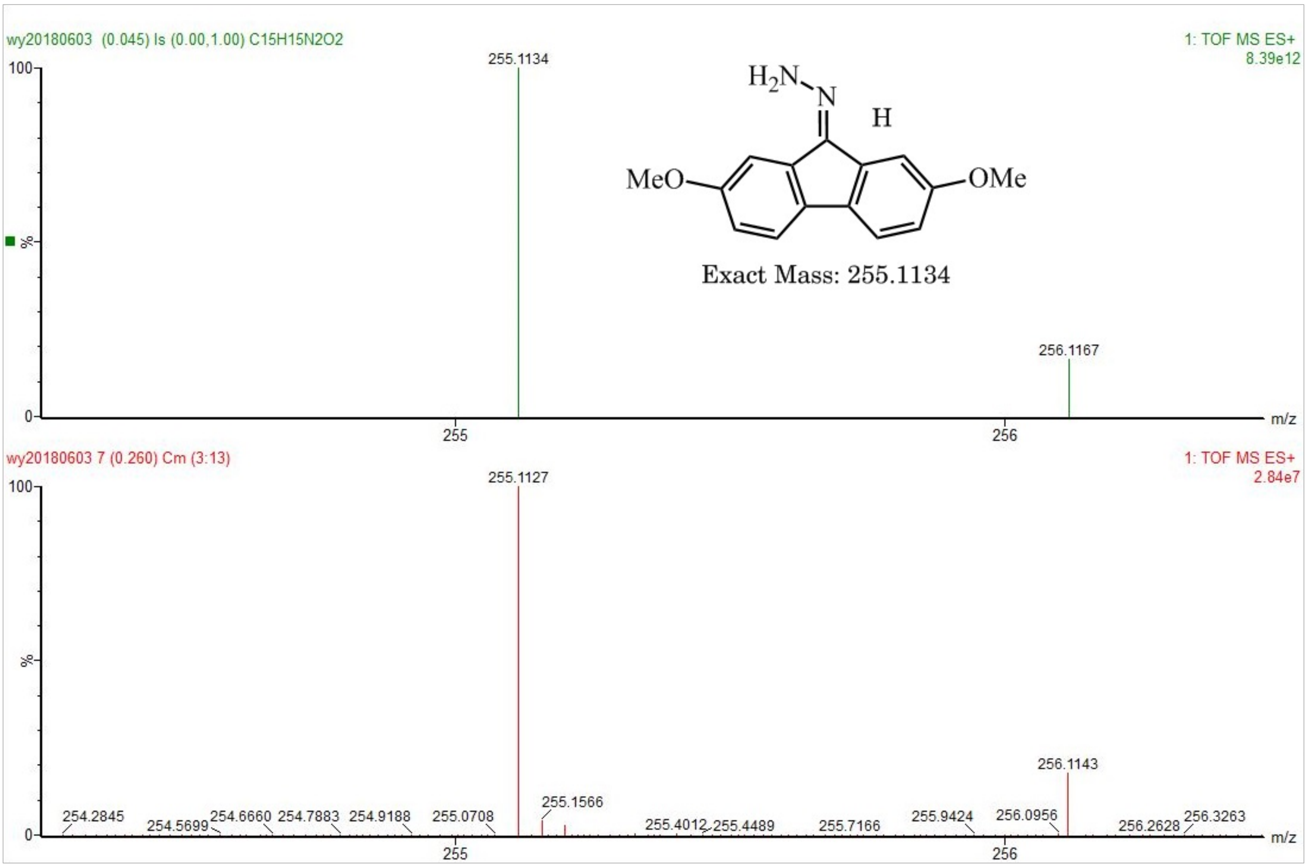

Supplementary Figure 23. HR ESI MS of **2c**. m/z = 255.1127.

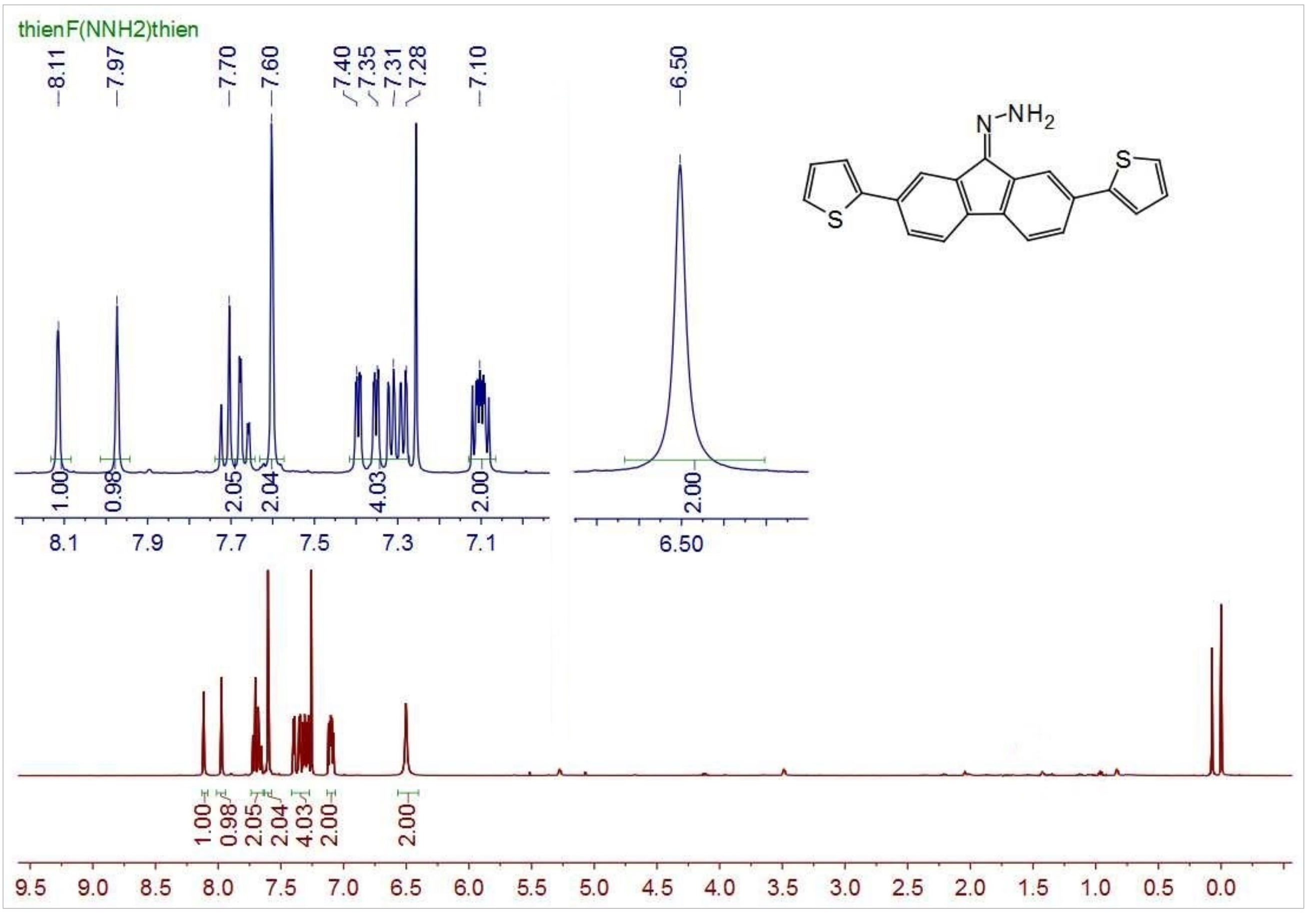

Supplementary Figure 24. ^{1}H NMR (400 MHz) of **2e** in $CDCl_3$.

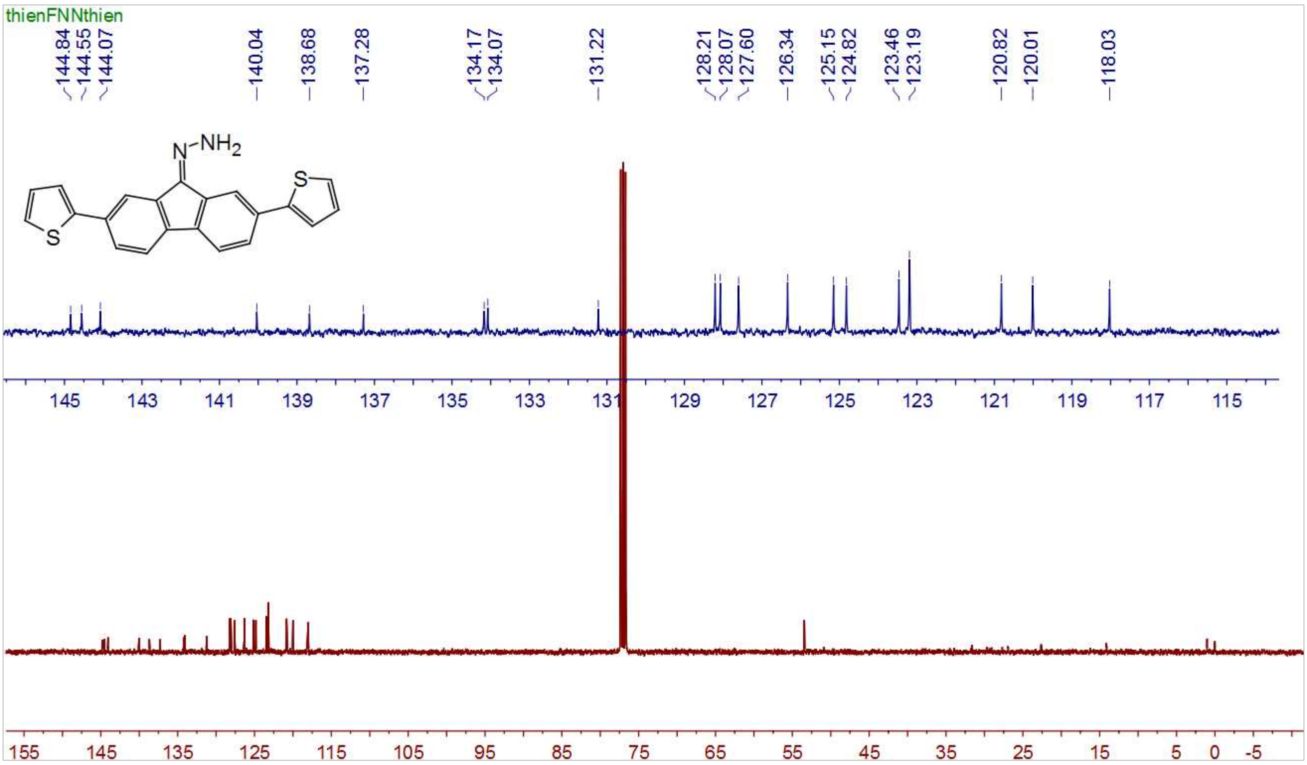

Supplementary Figure 25. ^{13}C NMR (100 MHz) of **2e** in $CDCl_3$.

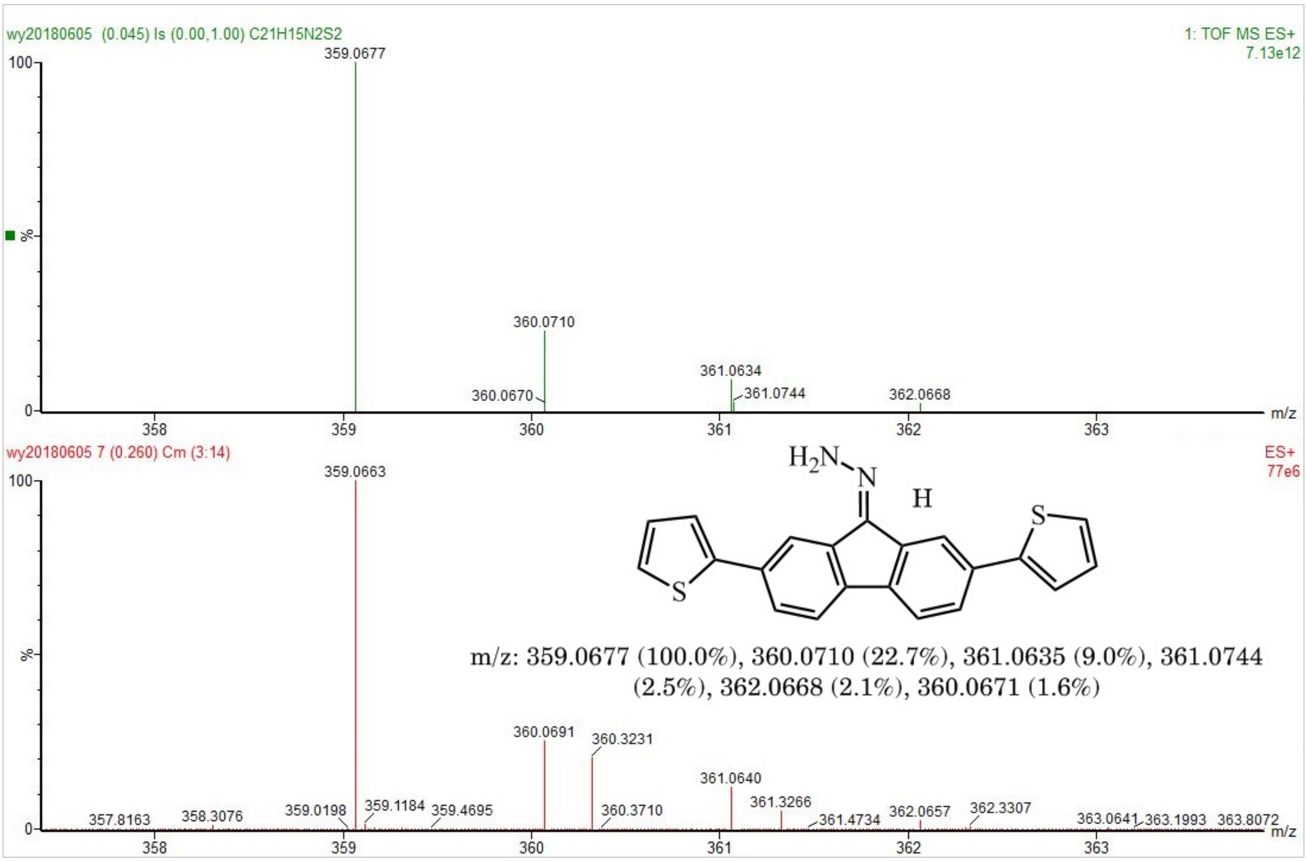

Supplementary Figure 26. HR ESI MS of **2e**. m/z = 359.0663.

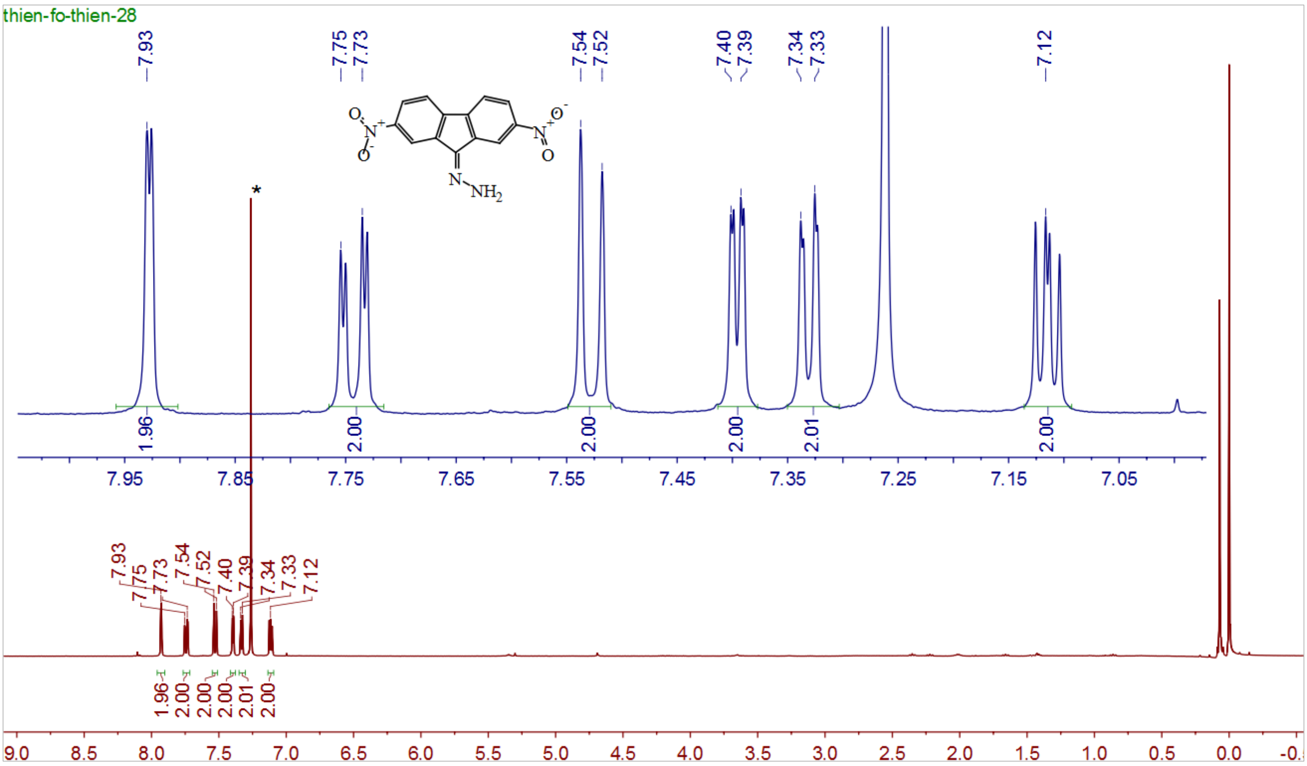

Supplementary Figure 27. ^{1}H NMR (400 MHz) of **2f** in $CDCl_3$.

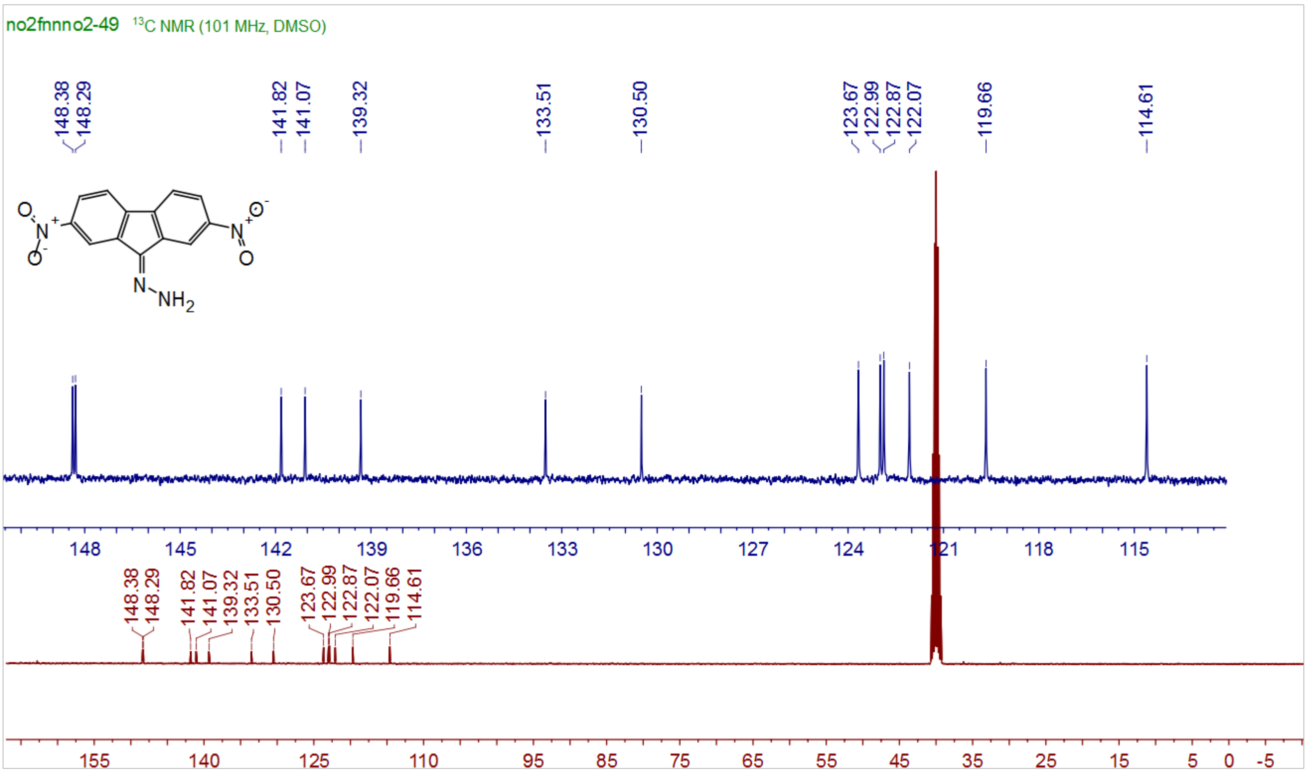

Supplementary Figure 28. ^{13}C NMR (100 MHz) of **2f** in DMSO-d_6.

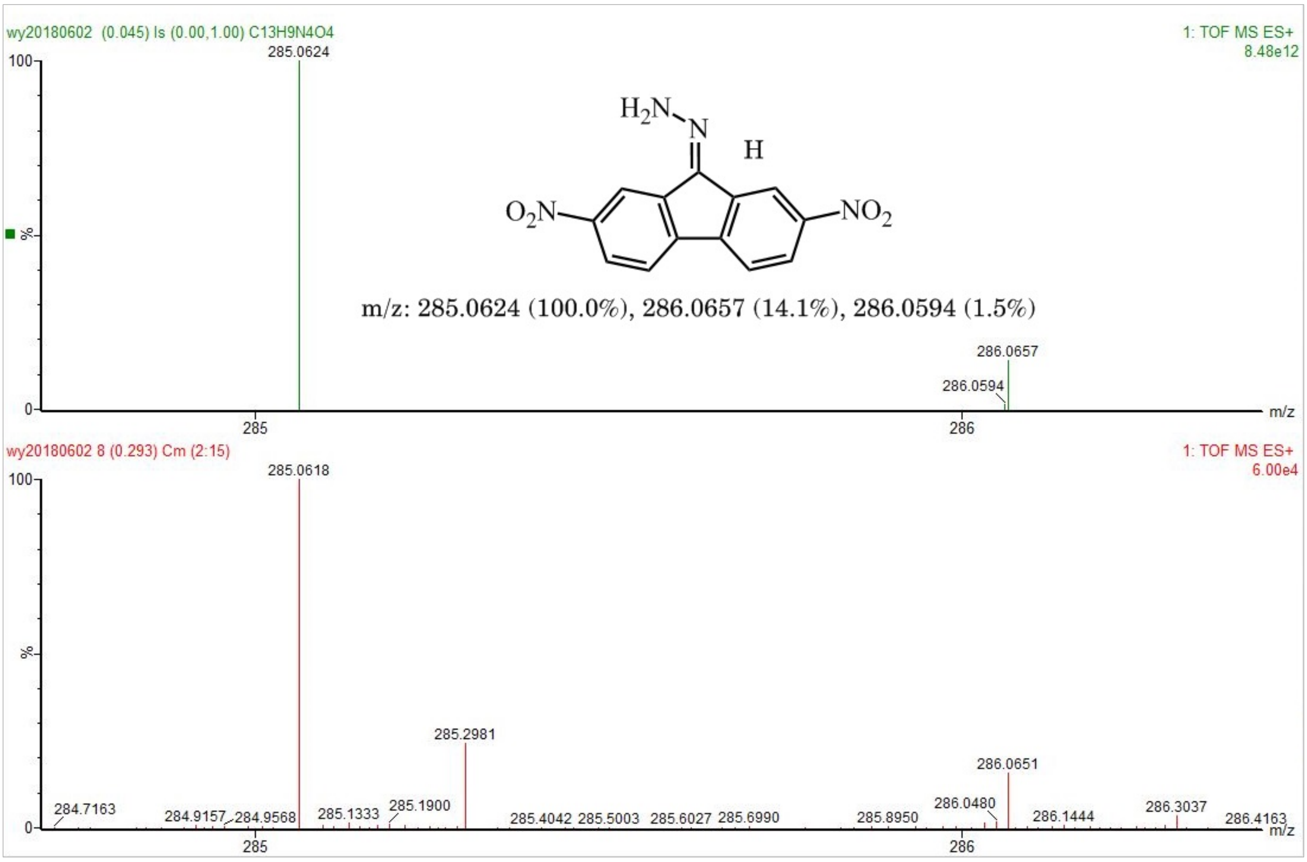

Supplementary Figure 29. HR ESI MS of **2f**. *m*/*z* = 285.0618.

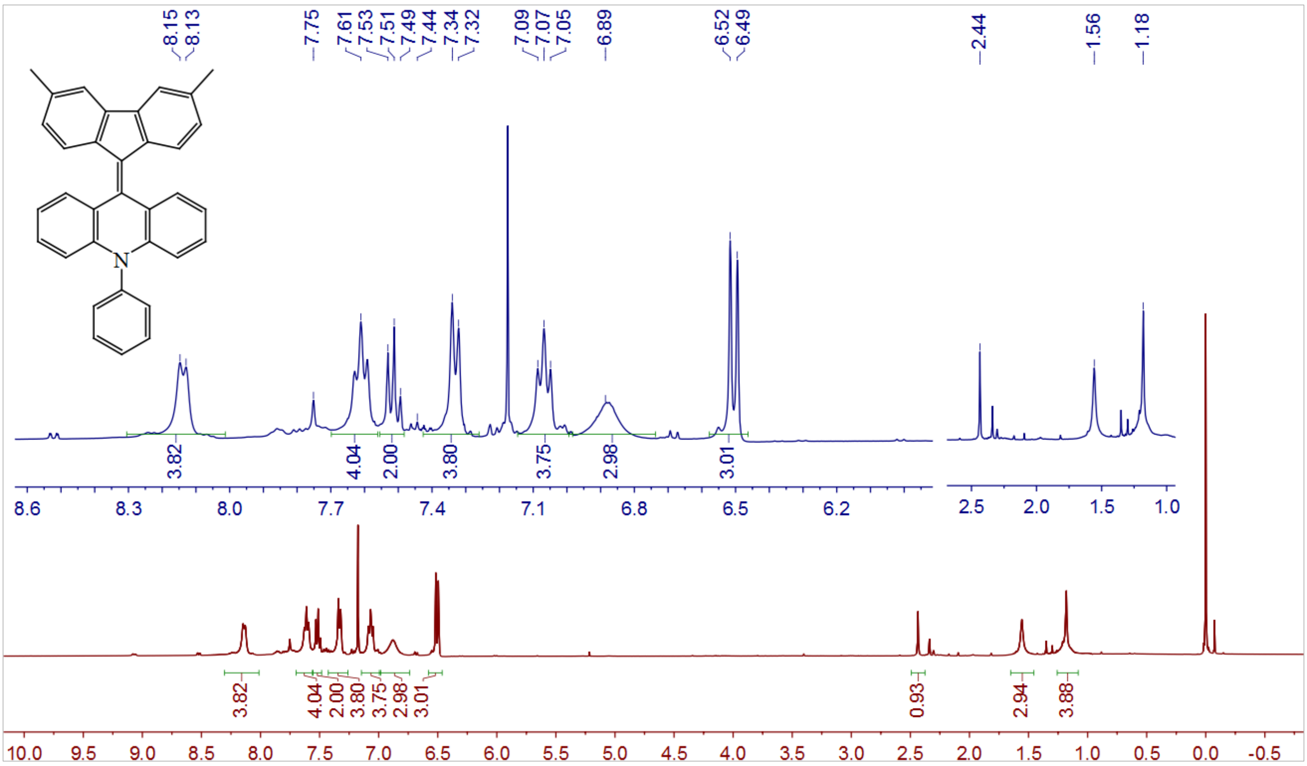

Supplementary Figure 30. ^{1}H NMR (400 MHz) of **5a** in $CDCl_3$.

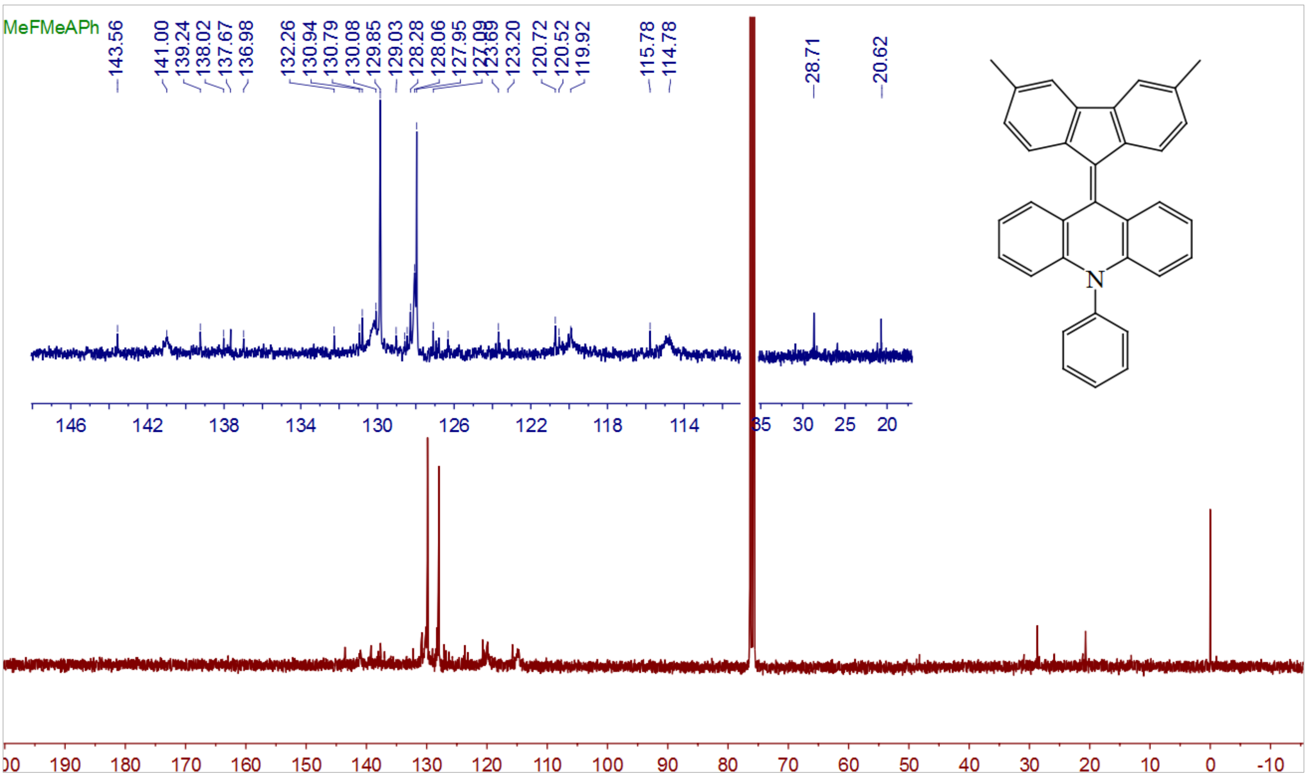

Supplementary Figure 31. ^{13}C NMR (100 MHz) of **5a** in $CDCl_3$.

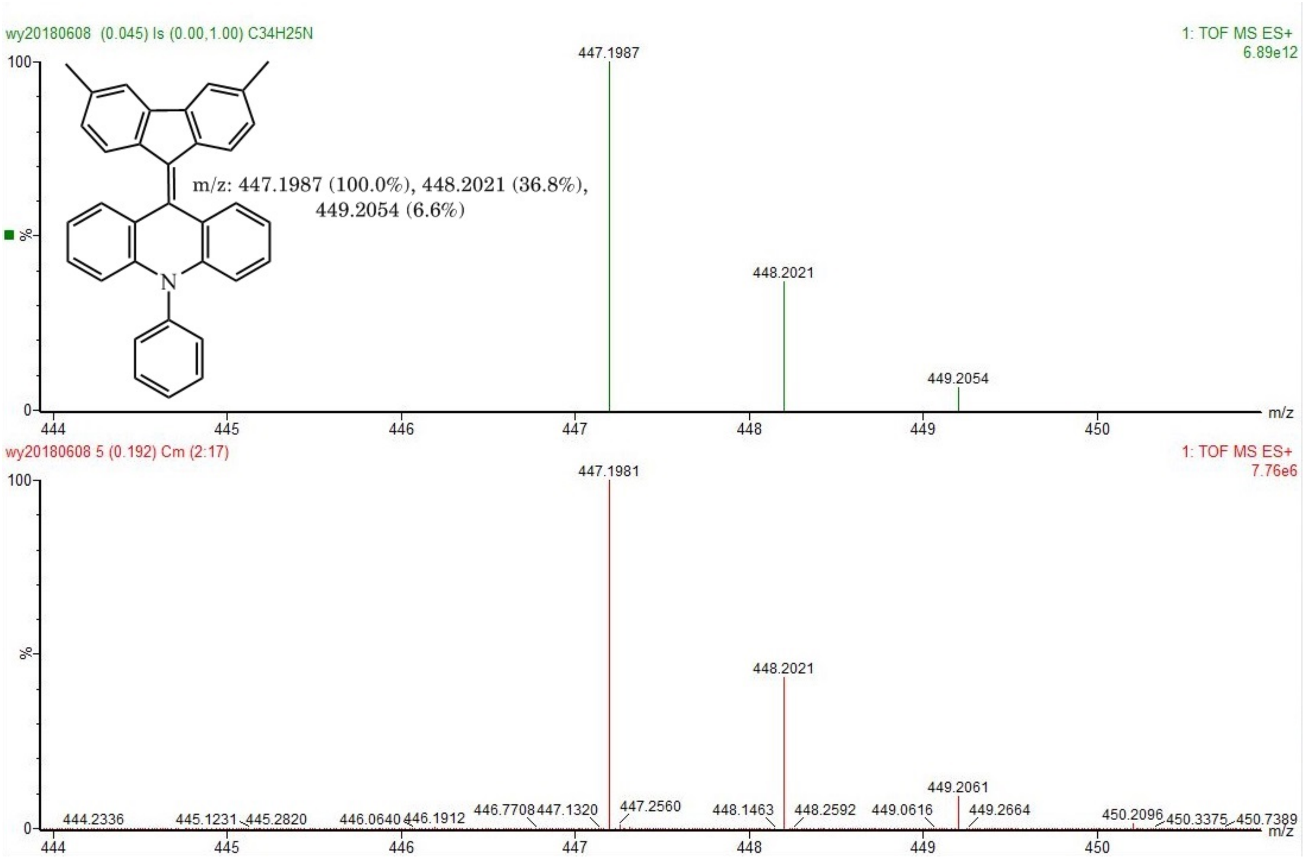

Supplementary Figure 32. HR ESI MS of **5a**. m/z = 447.1981.

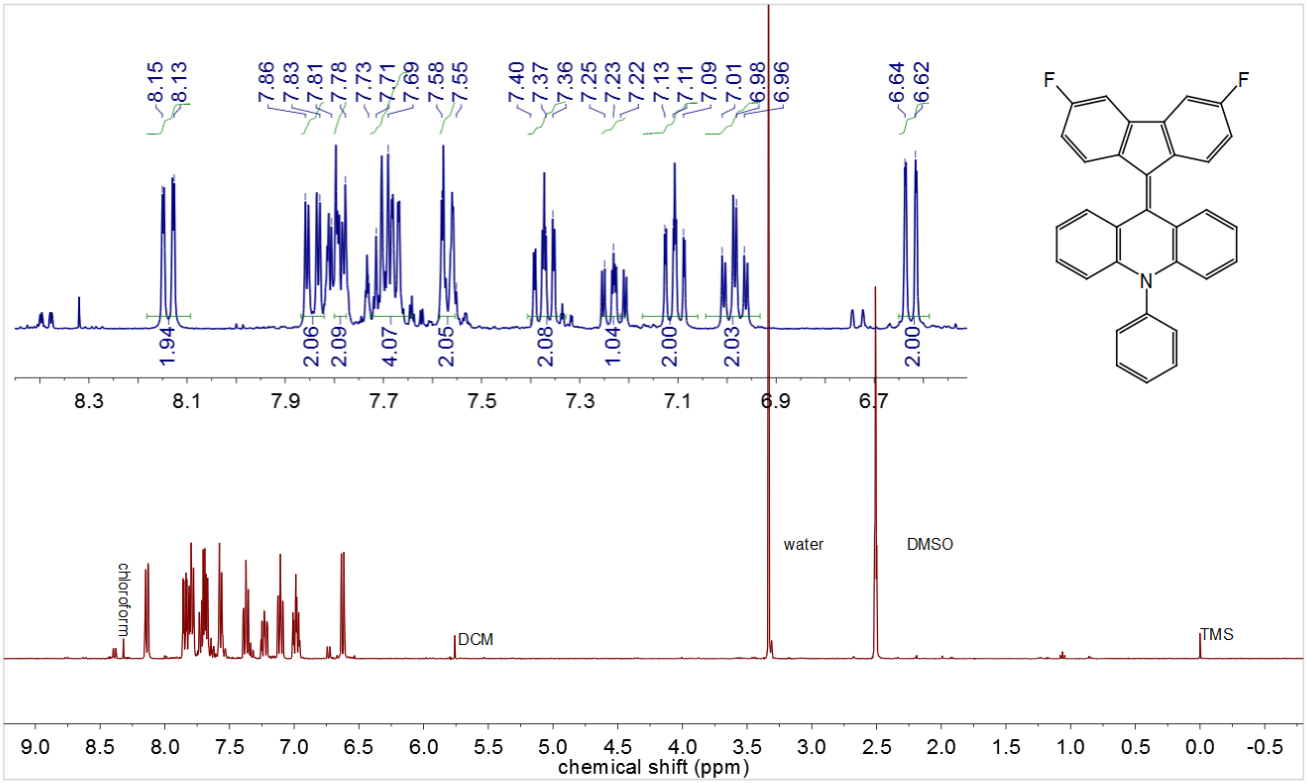

Supplementary Figure 33. ^{1}H NMR (400 MHz) of **5b** in DMSO-d_6.

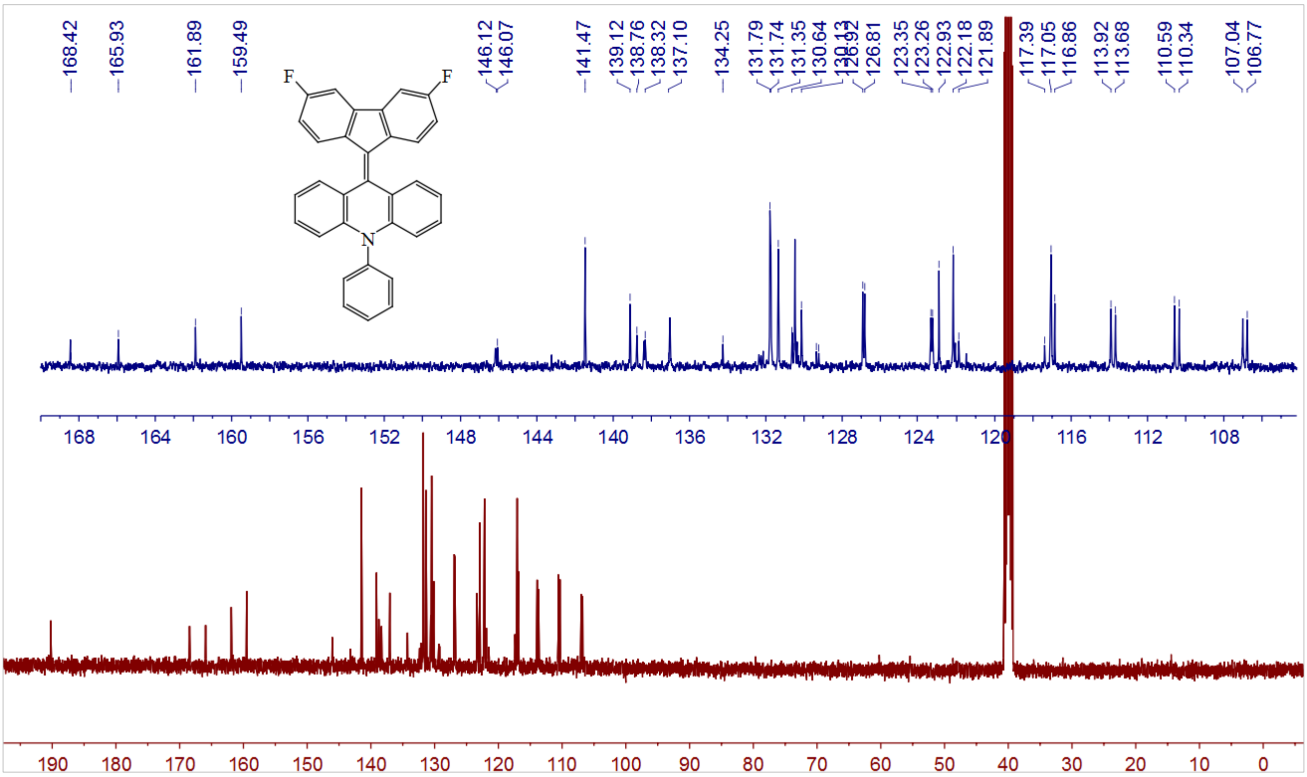

Supplementary Figure 34. ^{13}C NMR (100 MHz) of **5b** in DMSO-d_6.

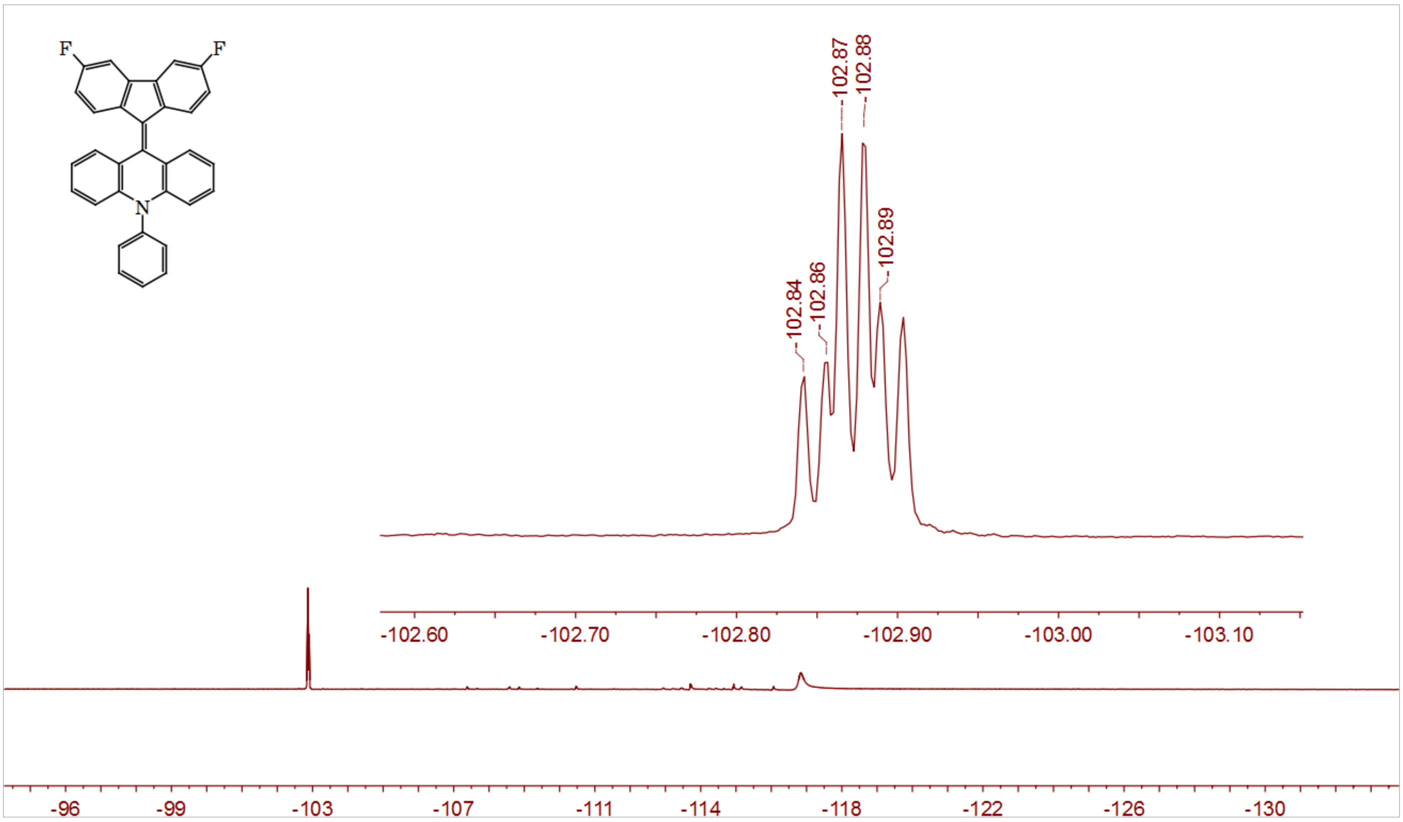

Supplementary Figure 35. ^{19}F NMR (400 MHz) of **5b** in DMSO-d_6.

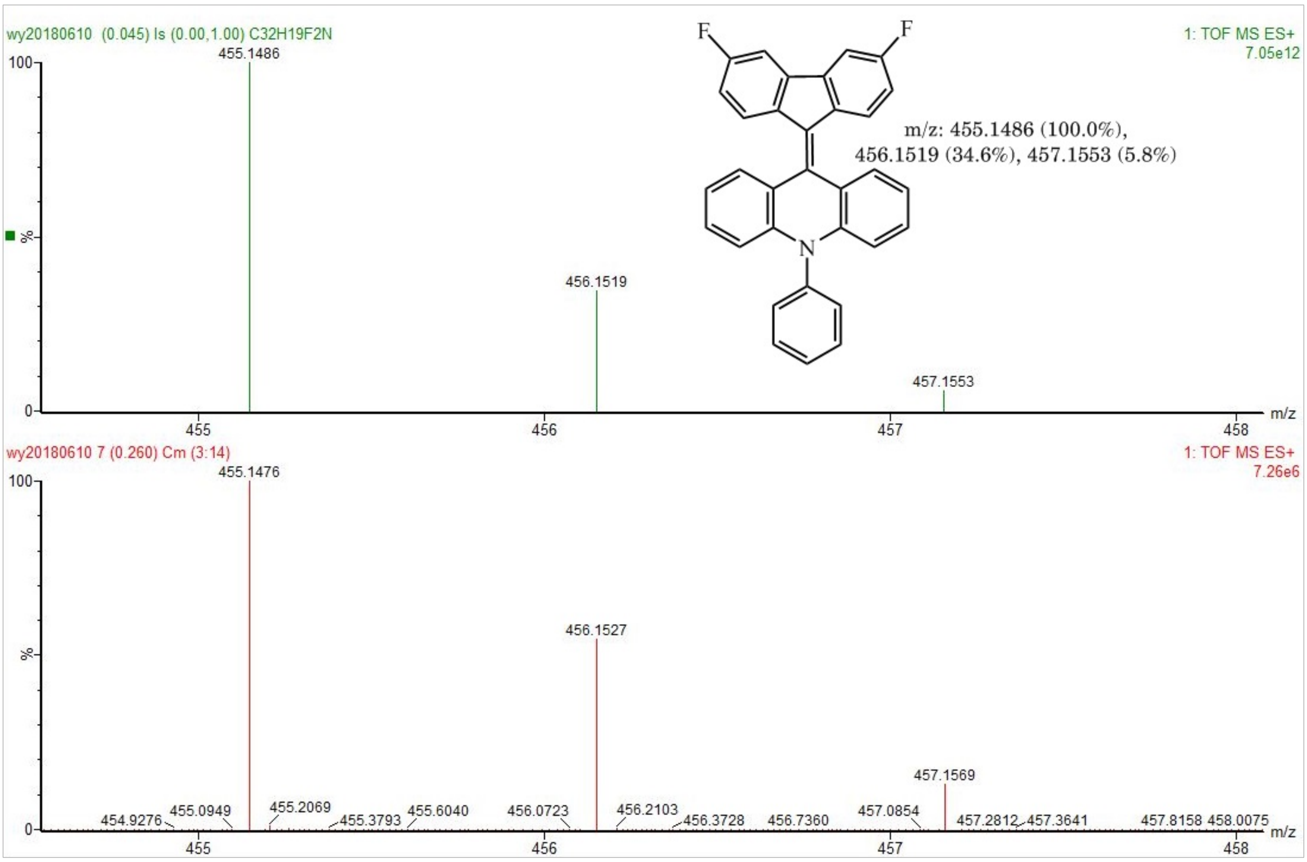

Supplementary Figure 36. HR ESI MS of **5b**. m/z = 455.1476.

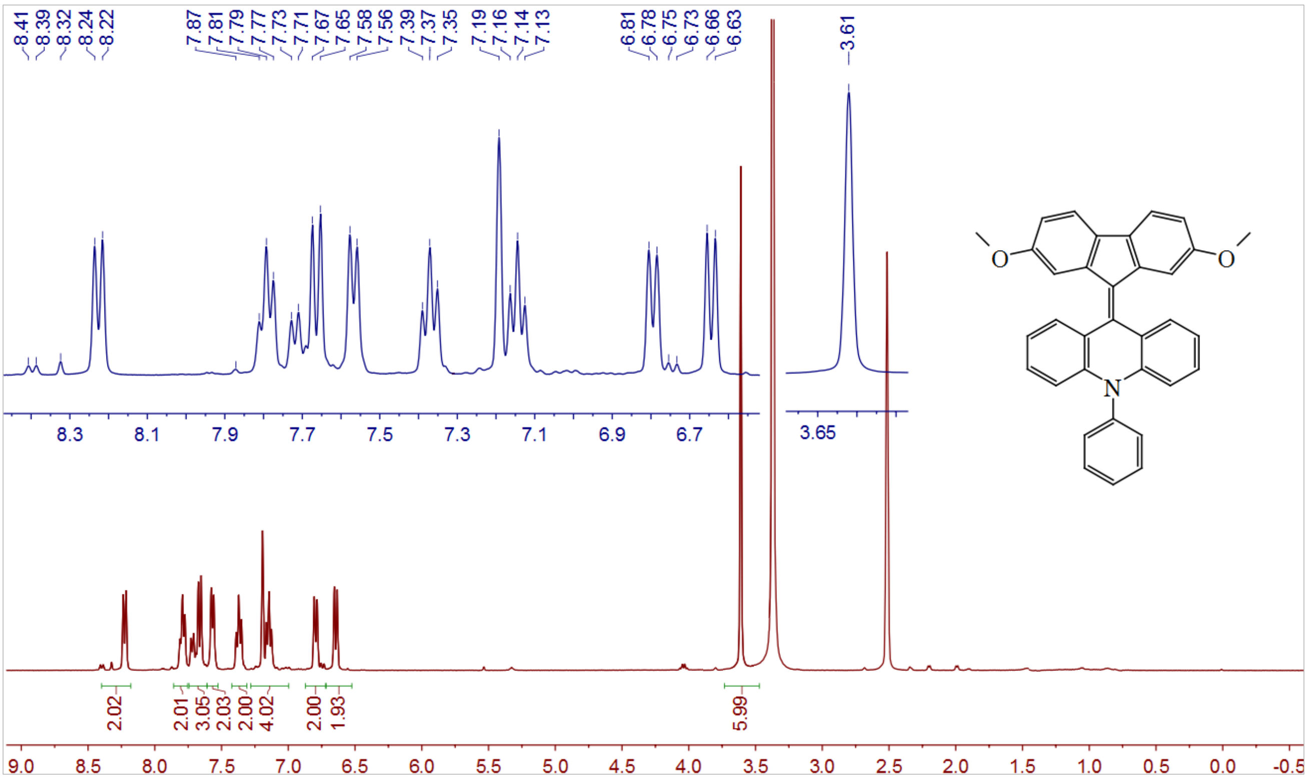

Supplementary Figure 37. ^{1}H NMR (400 MHz) of **5c** in DMSO-d_6.

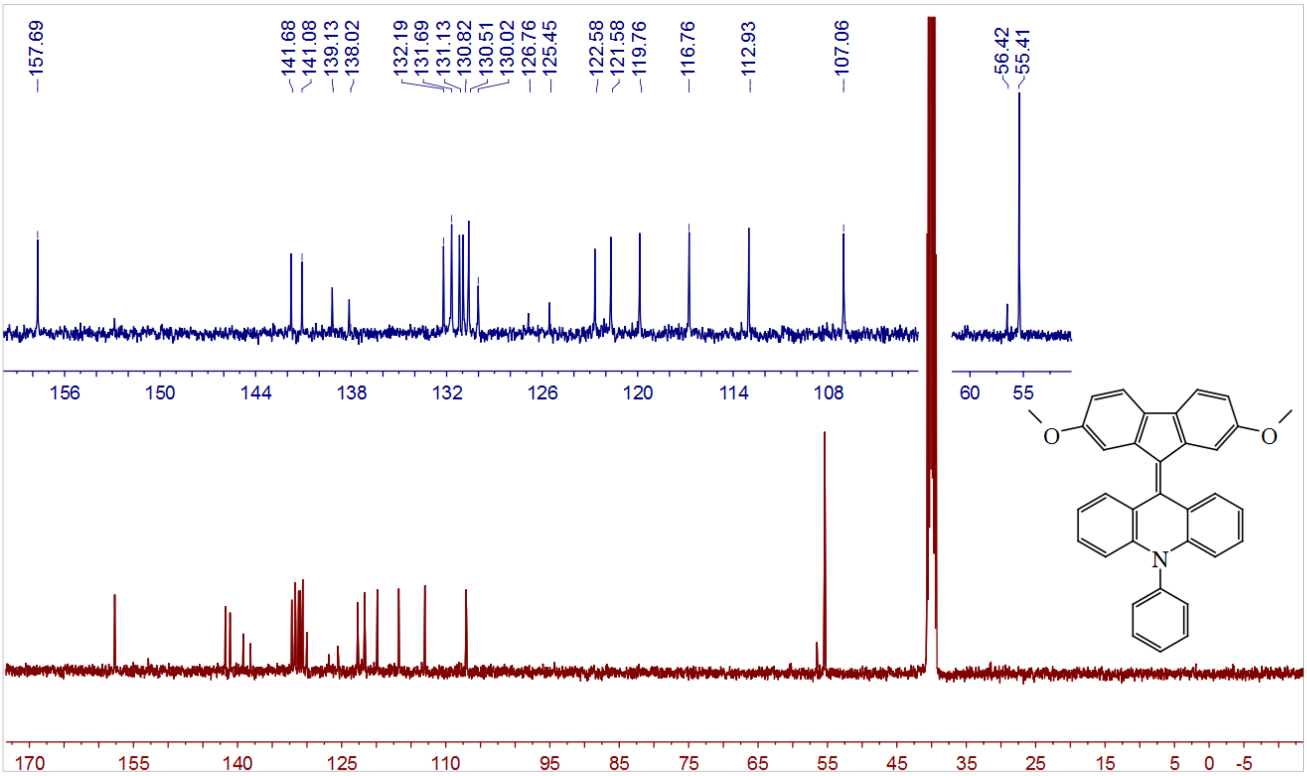

Supplementary Figure 38. ^{13}C NMR (100 MHz) of **5c** in DMSO-d_6.

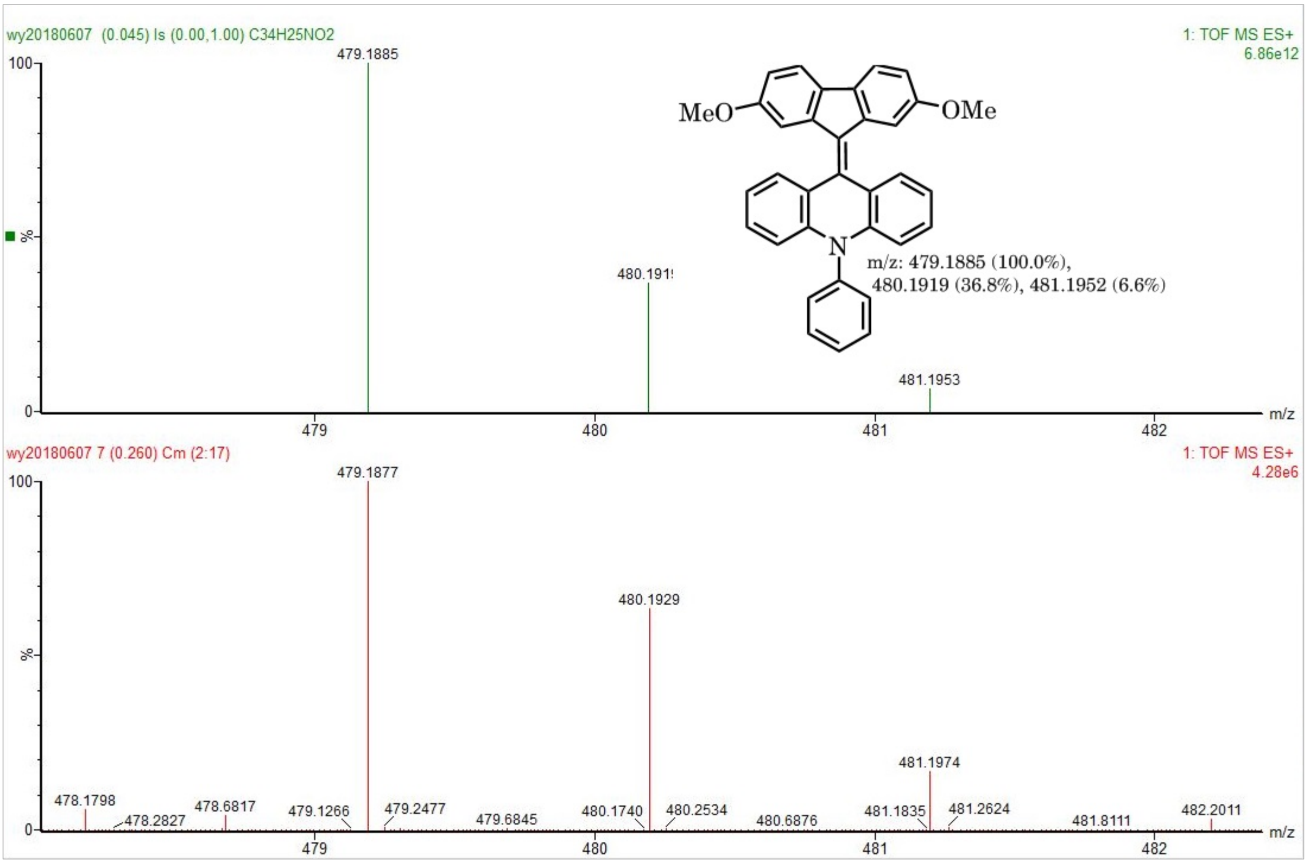

Supplementary Figure 39. HR ESI MS of **5c**. *m*/*z* = 479.1877.

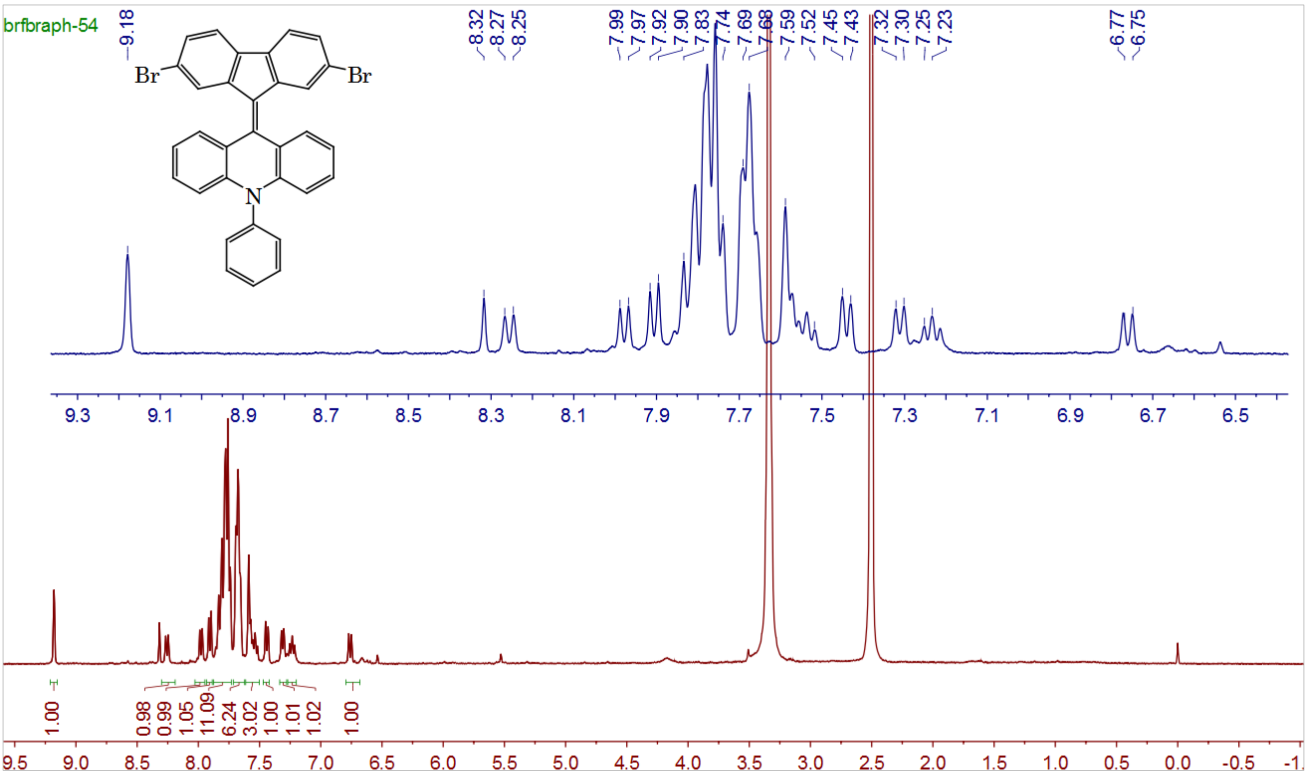

Supplementary Figure 40. ^{1}H NMR (400 MHz) of **5d** in DMSO-*d*$_6$.

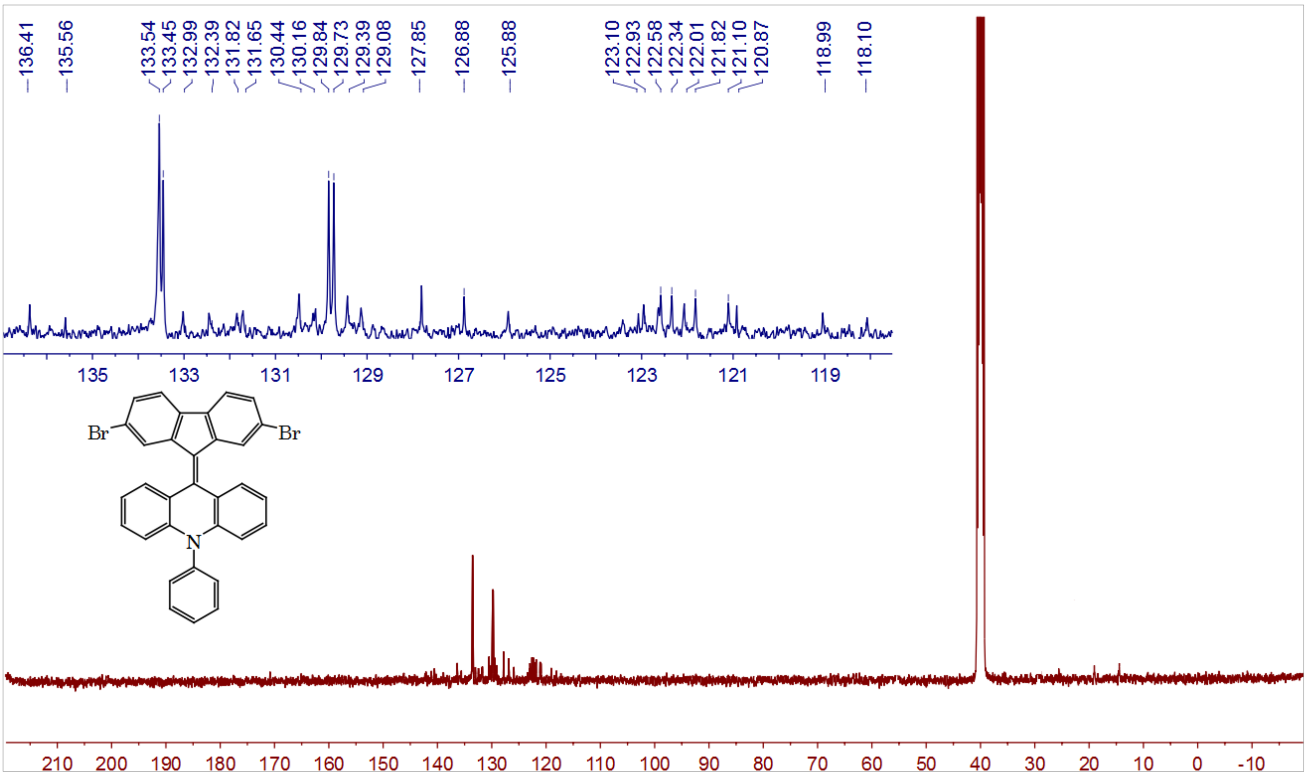

Supplementary Figure 41. ^{13}C NMR (100 MHz) of **5d** in DMSO-d_6.

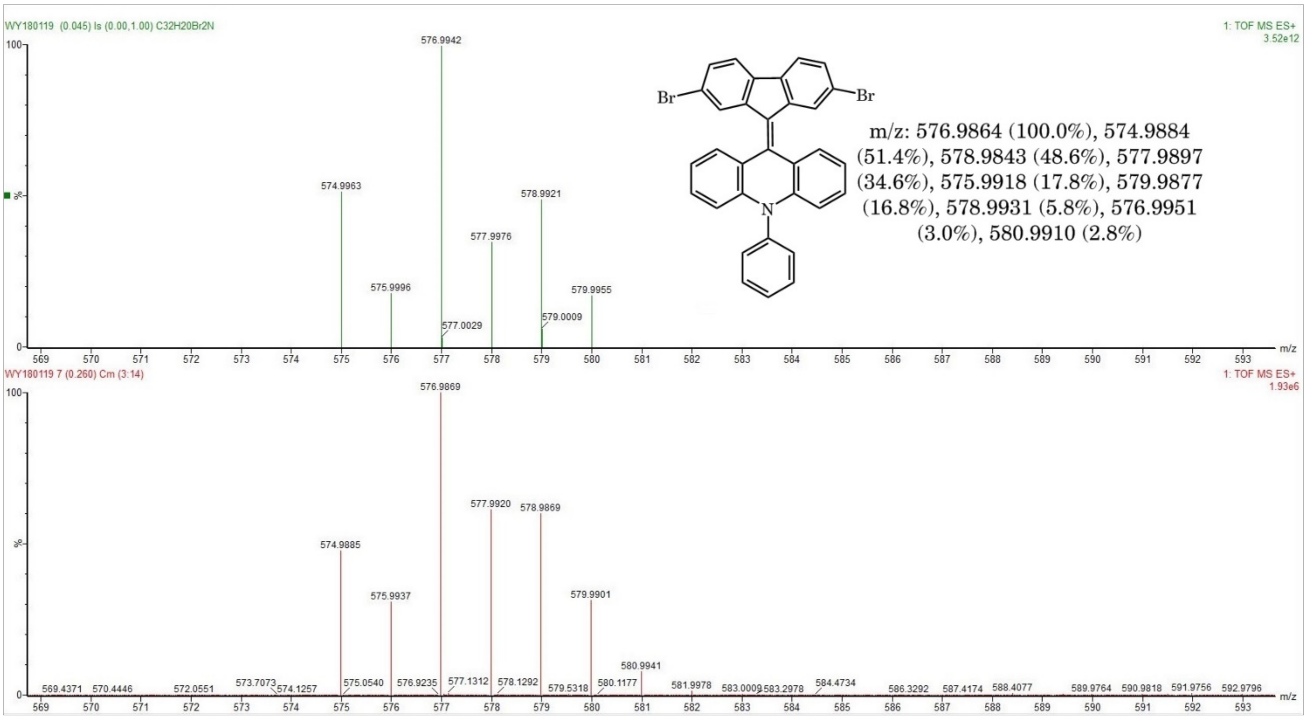

Supplementary Figure 42. HR ESI MS of **5d**. m/z = 576.9869.

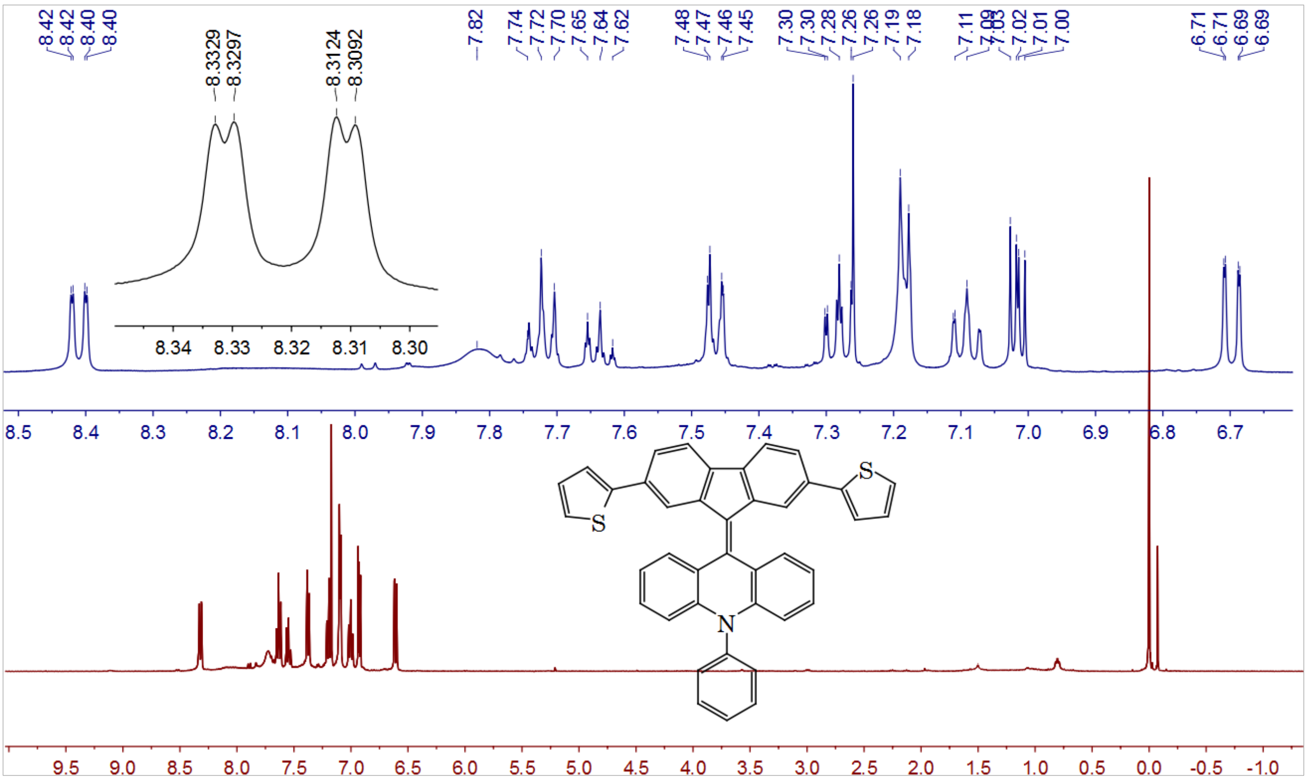

Supplementary Figure 43. ^{1}H NMR (400 MHz) of **5e** in $CDCl_3$.

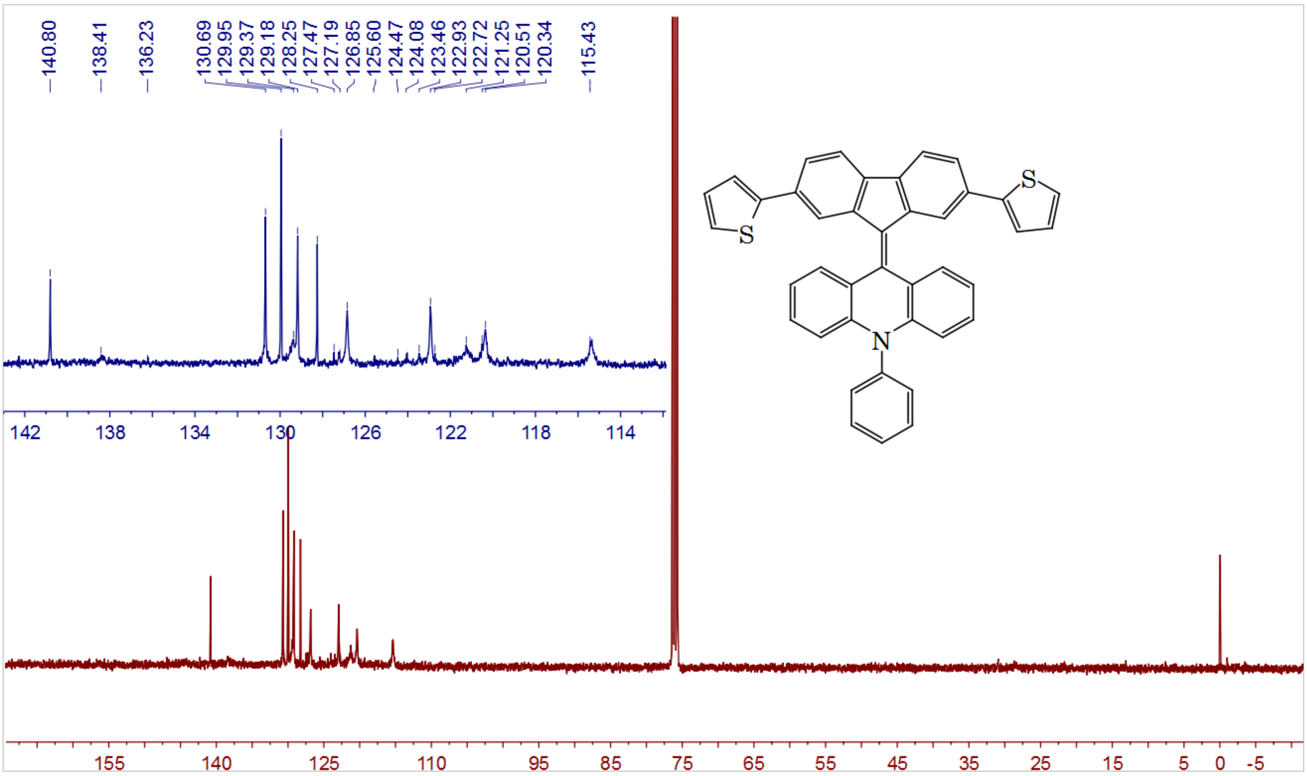

Supplementary Figure 44. ^{13}C NMR (100 MHz) of **5e** in $CDCl_3$.

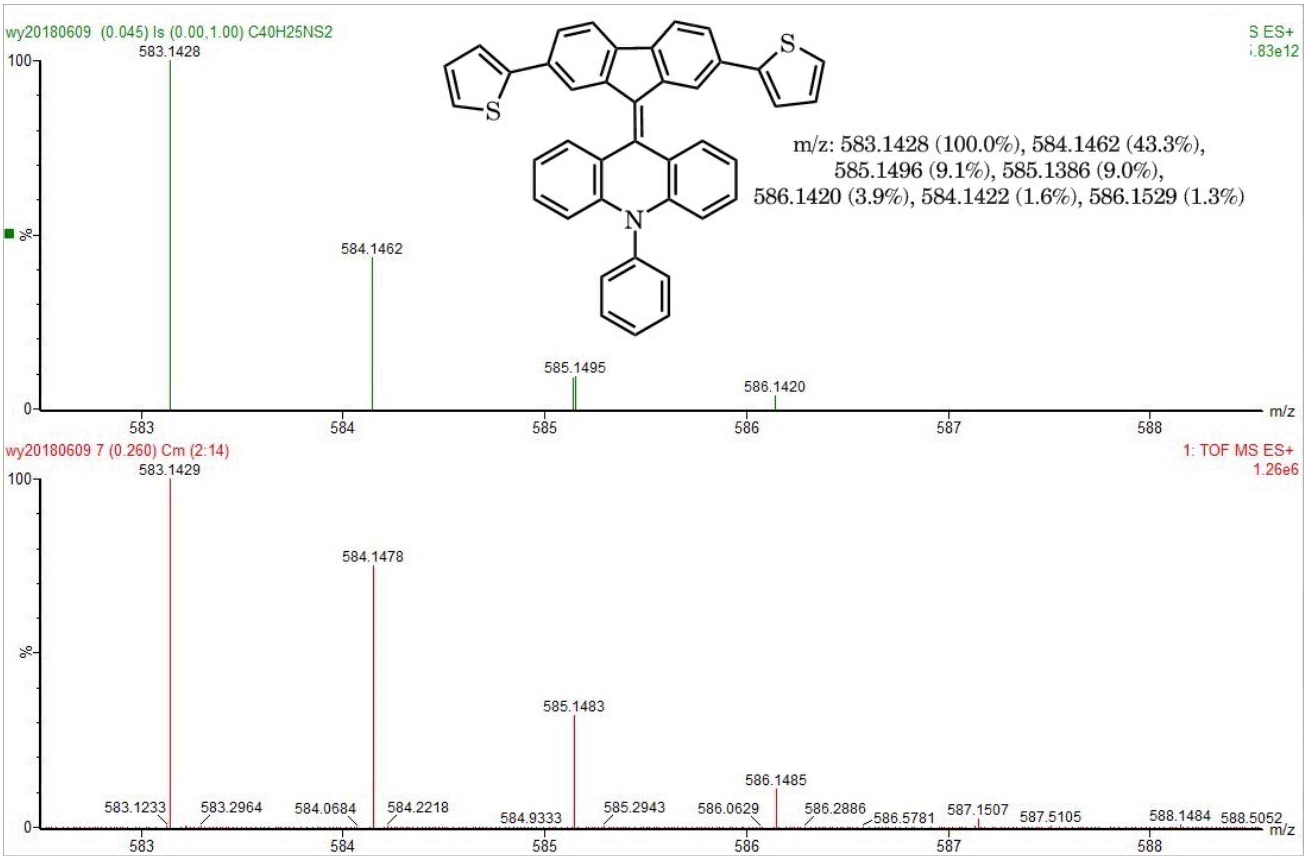

Supplementary Figure 45. HR ESI MS of **5e**. m/z = 583.1429.

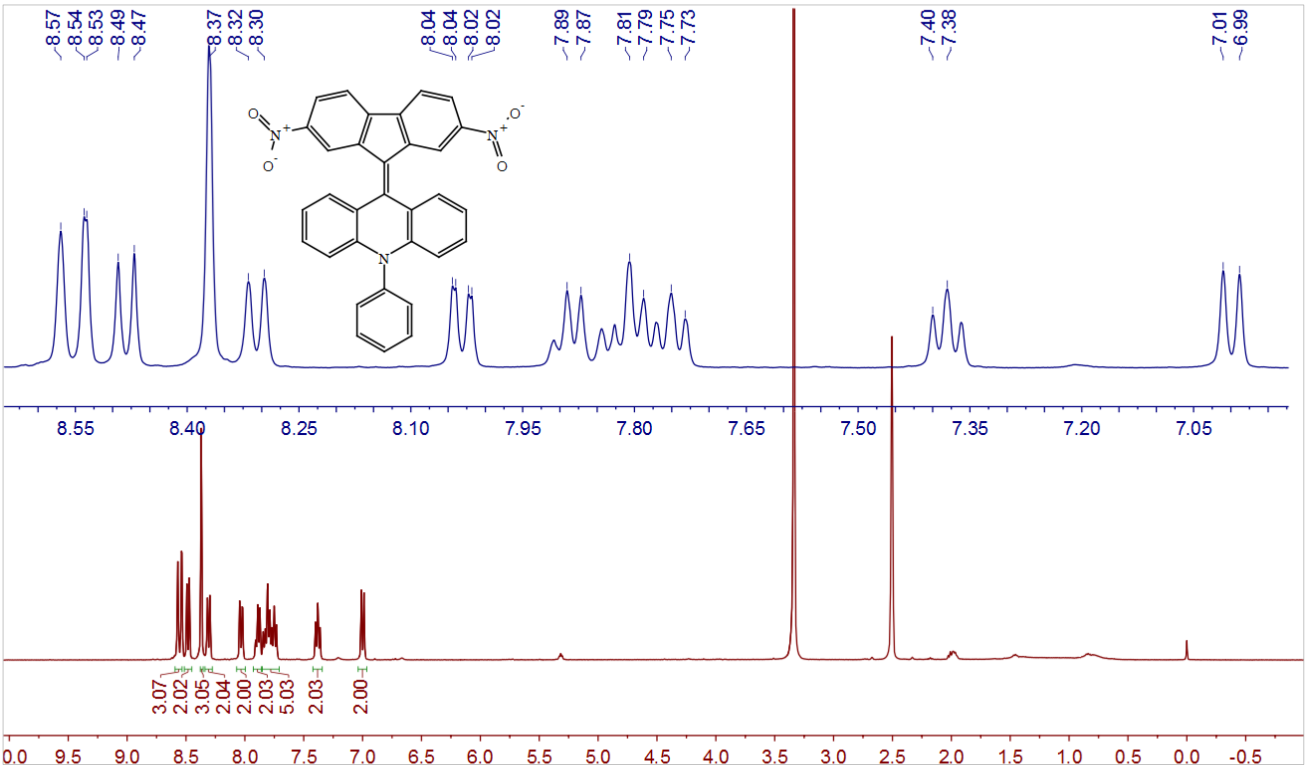

Supplementary Figure 46. ^{1}H NMR (400 MHz) of **5f** in DMSO-d_6.

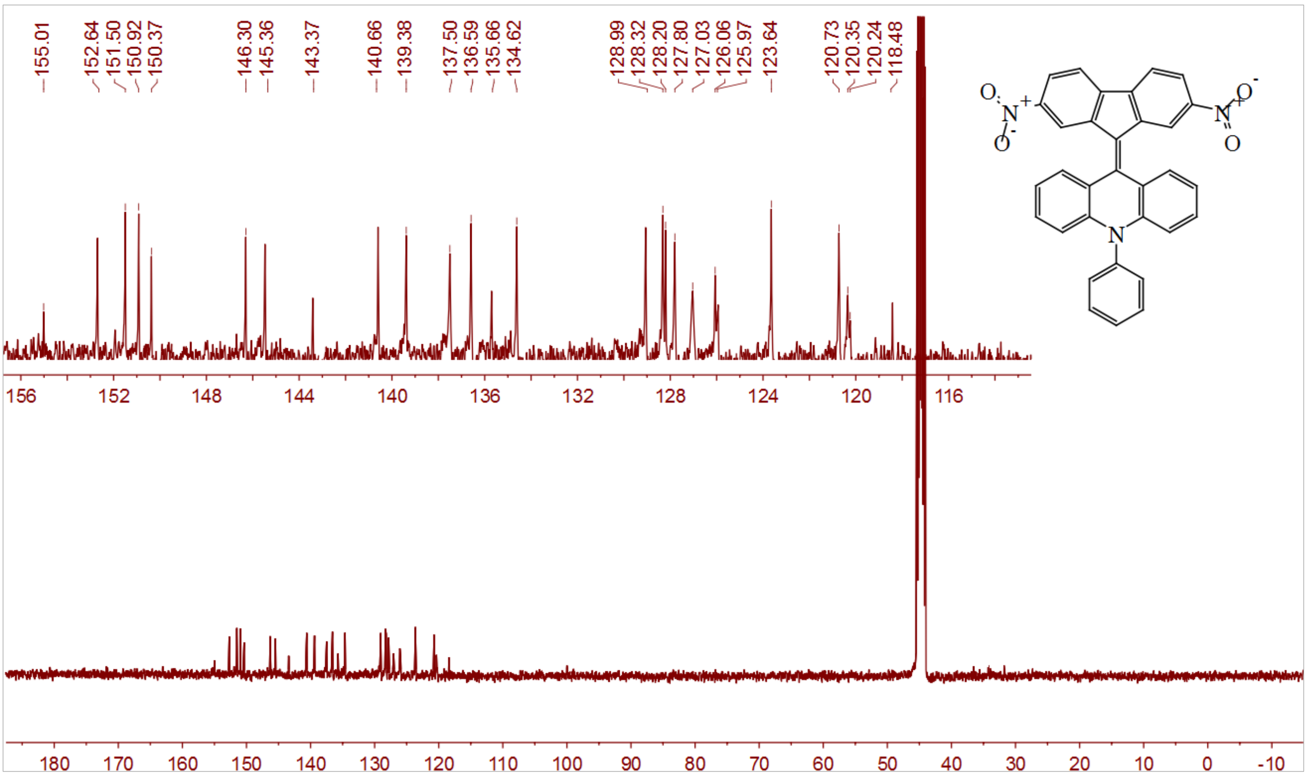

Supplementary Figure 47. ^{13}C NMR (100 MHz) of **5f** in DMSO-d_6.

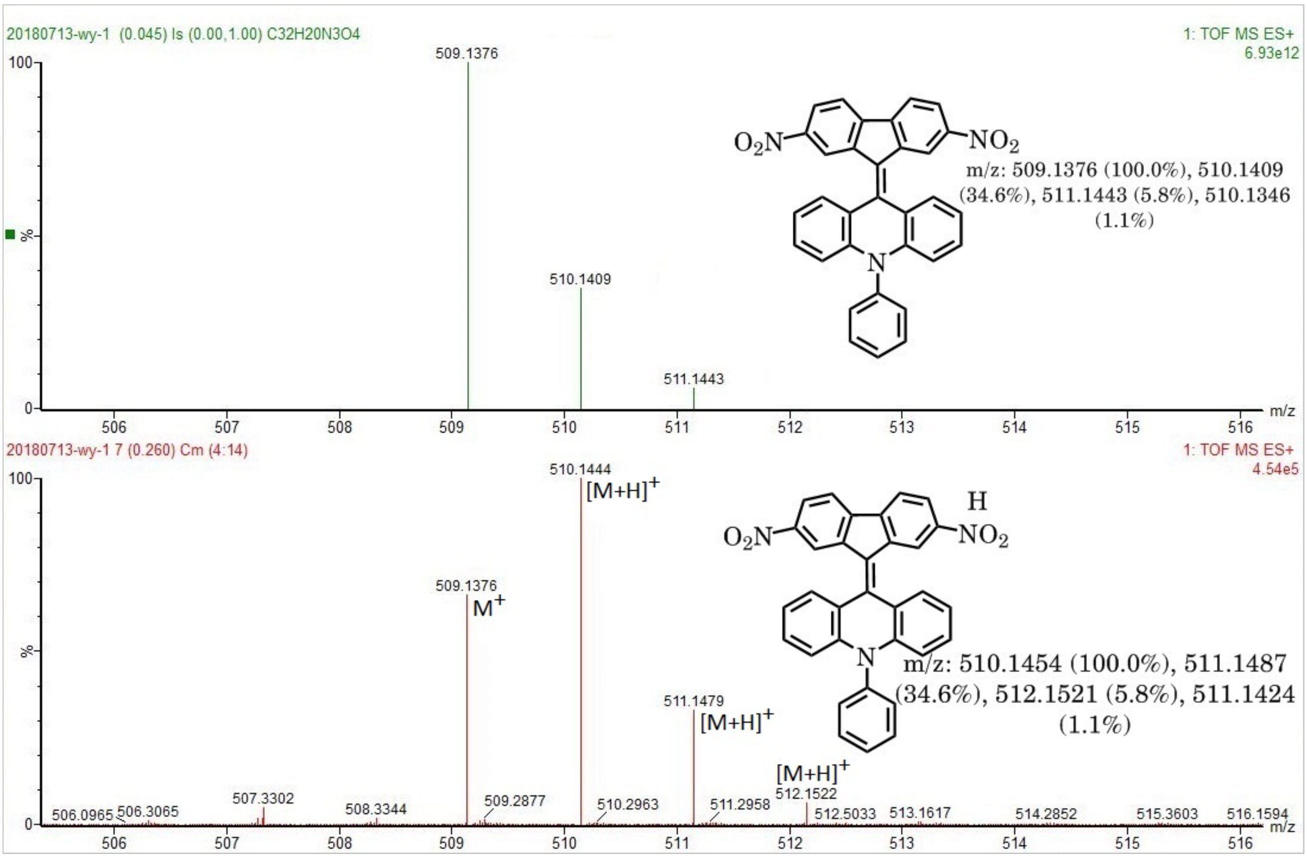

Supplementary Figure 48. HRMS (ESI/TOF) of **5f**. *m/z*: $[M+H]^+$ *m/z* = 510.1444.

3. Thermal gravimetric Analysis (TGA) and differential scanning calorimetry (DSC) data

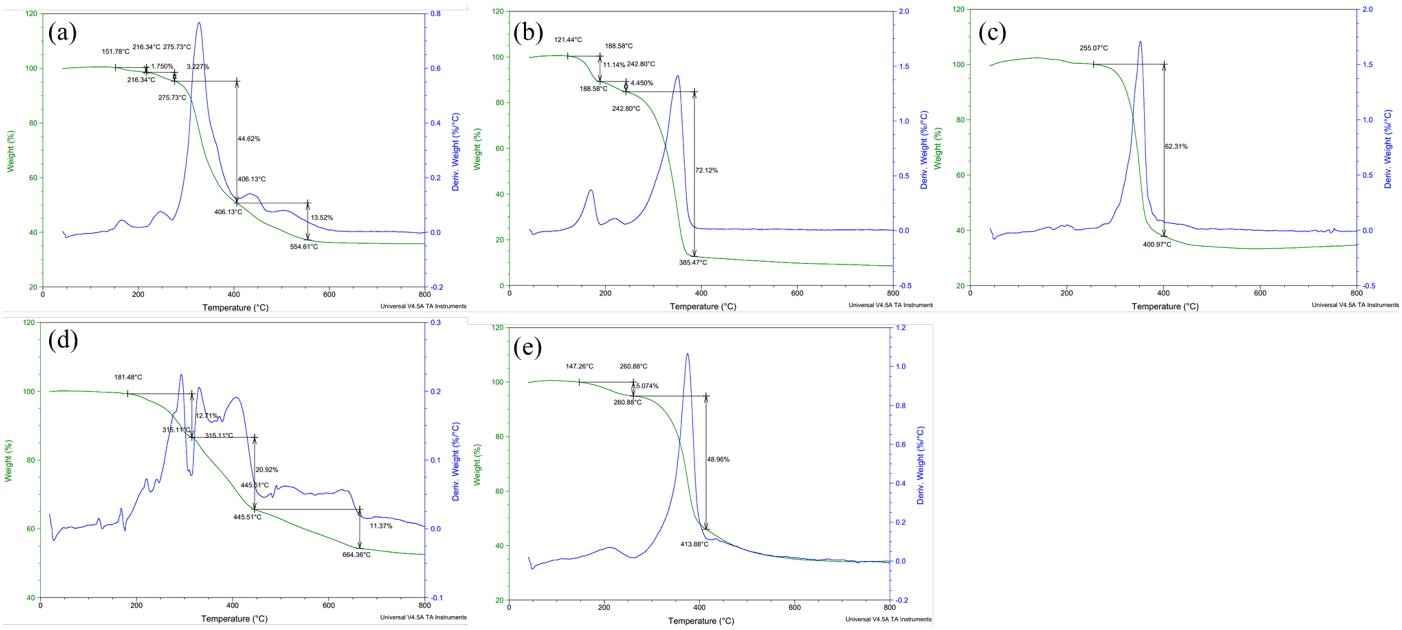

Supplementary Figure 49. Thermal gravimetric analysis (TGA) data of X_2FAPhs. TGA traces of compounds (a) **5a**, (b) **5b**, (c) **5c**, (d) **5d**, and (e) **5e**. Decomposition temperature of **3a**, **3b**, **3c**, **3d** and **3e** were 216.34 ºC, 242.80 ºC, 255.07 ºC, 315.11 ºC, and 260.88 ºC.

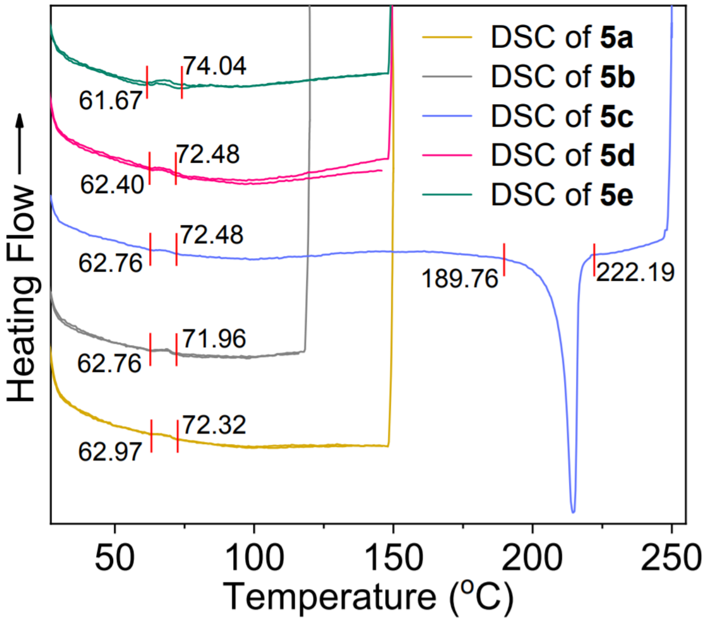

Supplementary Figure 50. Differential scanning calorimetry (DSC) data of FAs. Brown, gray, blue, red, and green lines stand for DSC curves of **5a, 5b**, **5c**, **5d**, and **5e**, respectively. The first scan of the heating and cooling processes are shown. The sharp endothermic peak found in **5c** indicates melting process. Glass transitions were observed around 62–74 ºC.

4. Light Absorption Properties

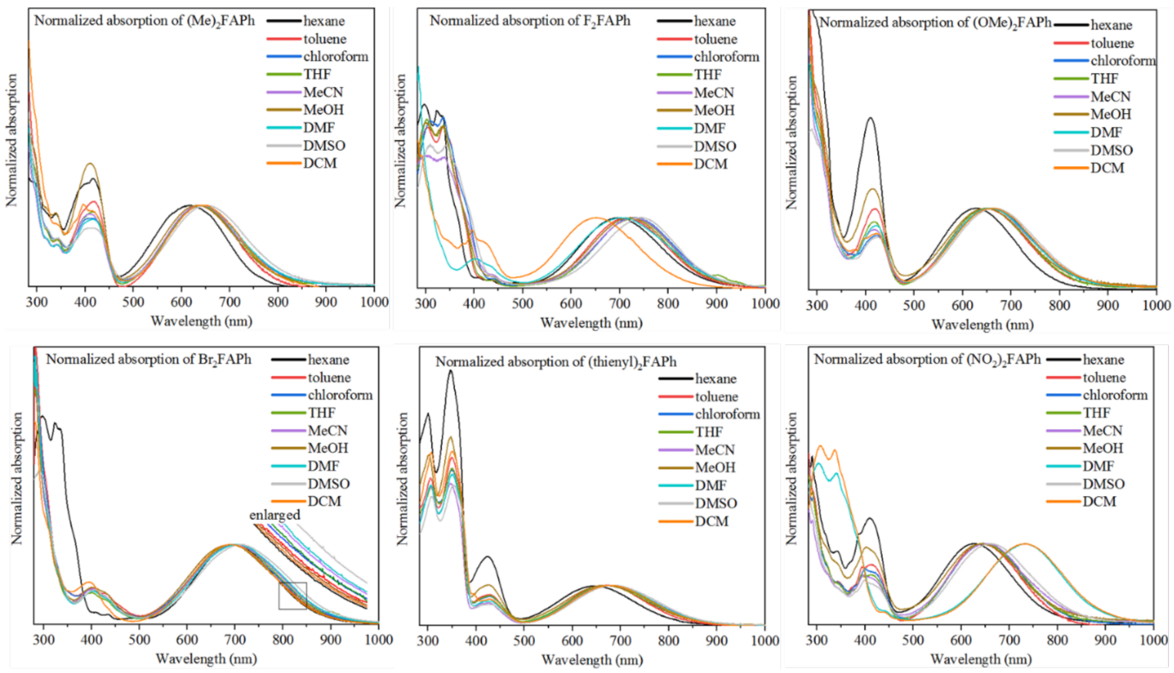

Supplementary Figure 51. Normalized UV-Vis absorption spectra in different solvent. (a) Me$_2$FAPh (**5a**), F$_2$FAPh, (b) (**5b**), (c) (OMe)$_2$FAPh (**5c**), (d) Br$_2$FAPh (**5d**), (e) (thienyl)$_2$FAPh (**5e**), (f) (NO$_2$)$_2$FAPh (**5f**). Solvent polarity influences on equilibrium constant between the folded conformer (yellow-brown, 300–450 nm) and the twisted conformer (green-blue, 500–800 nm). For all six compounds, the folded conformer contents became maximum in *n*-hexane.

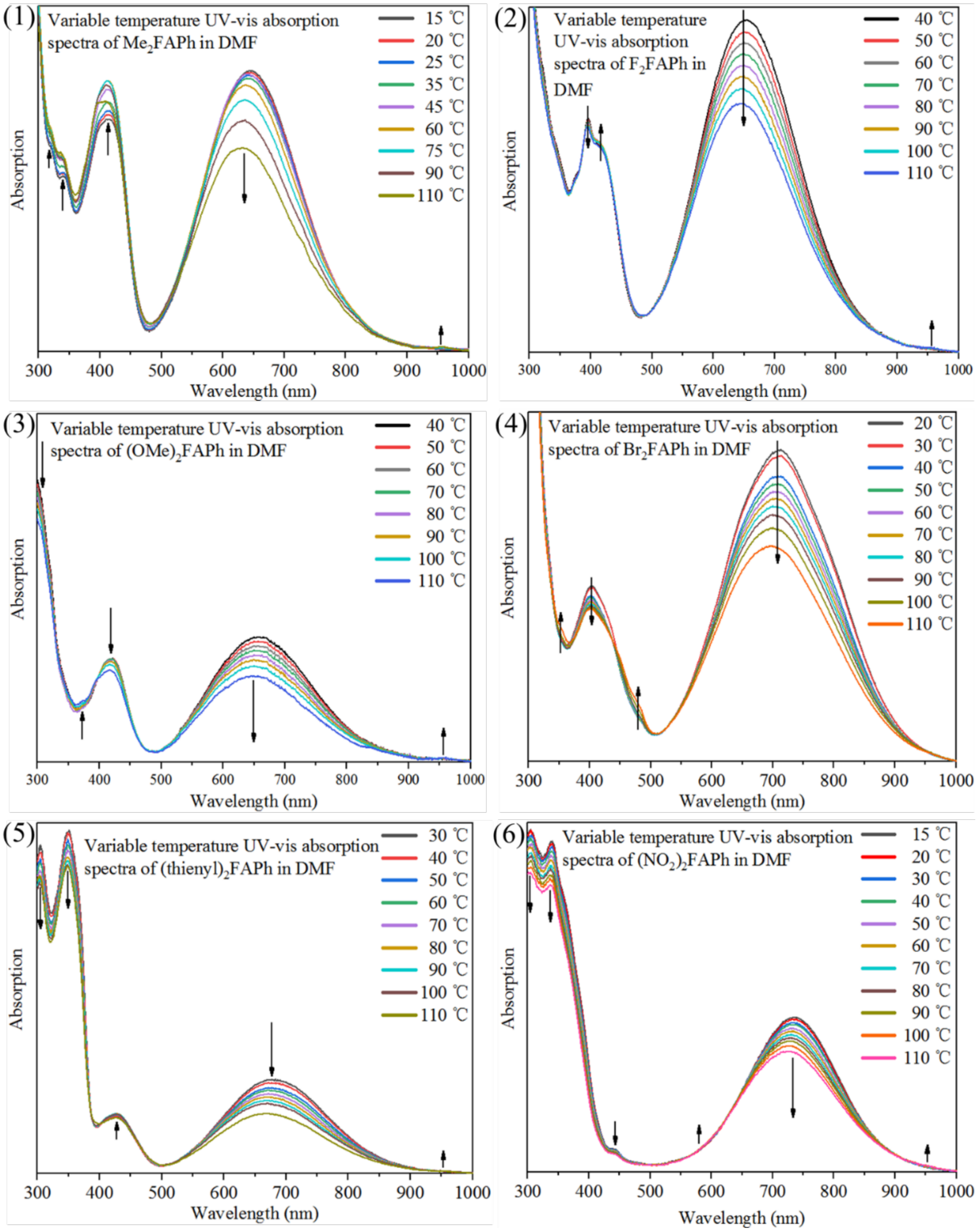

Supplementary Figure 52. Variable temperature UV-Vis absorption spectra in different temperature range. (1) Me_2FAPh (**5a**). F_2FAPh, (2) (**5b**), (3) $(OMe)_2FAPh$ (**5c**), (4) Br_2FAPh (**5d**), (5) $(thienyl)_2FAPh$ (**5e**), (6) $(NO_2)_2FAPh$ (**5f**). There was no difference between the absorption spectrum measured as soon as the sample had been heated to 100 °C and the one measured after heating for 1 hour at 100 °C, so we considered the system with the increased folded conformer concentration due to heating to be at equilibrium.

5. Fluorescence Properties

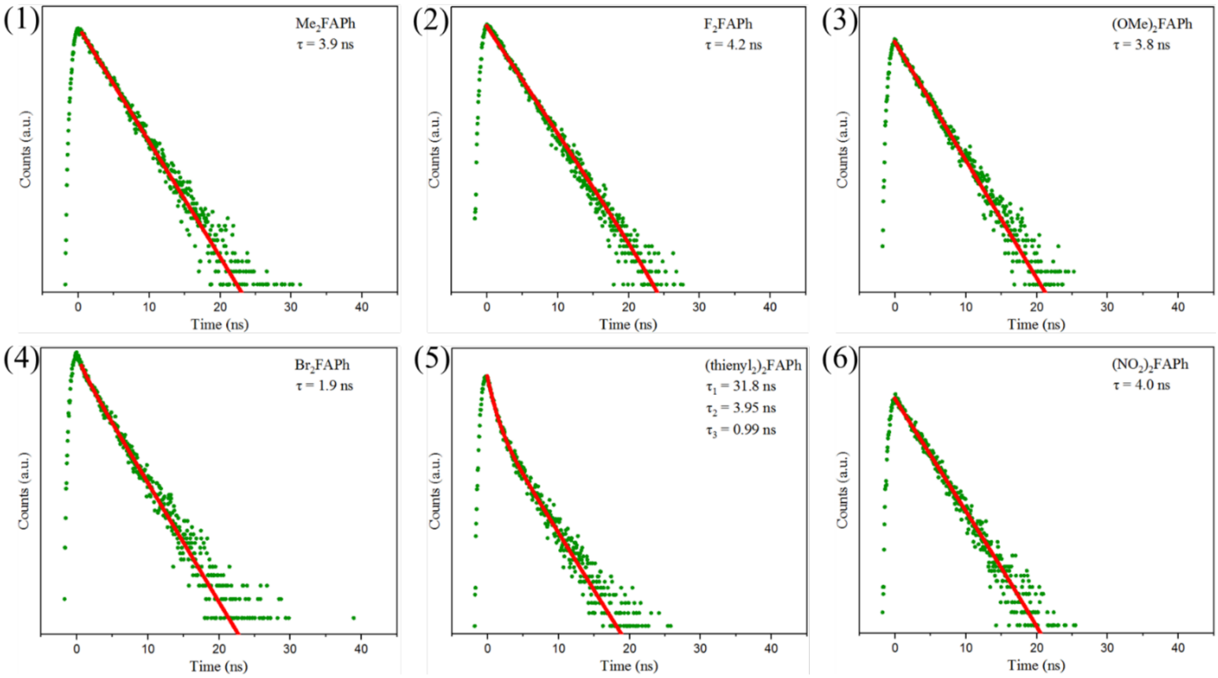

Supplementary Figure 53. Fluorescence lifetime of X_2FAPh in dichloromethane. (1) Me_2FAPh (**5a**), $\tau = 3.9$ ns, (2) F_2FASPh (**5b**), $\tau = 4.2$ ns, (3) $(OMe)_2$FASPh (**5c**), $\tau = 3.8$ ns, (4) Br_2FAPh (**5d**), $\tau = 1.9$ ns, (5) $(thienyl)_2$FAPh (**5e**), $\tau_1 = 31.8$ ns, $\tau_2 = 3.95$ ns, $\tau_3 = 0.99$ ns, (6) $(NO_2)_2$FAPh (**5f**), $\tau = 4.0$ ns.

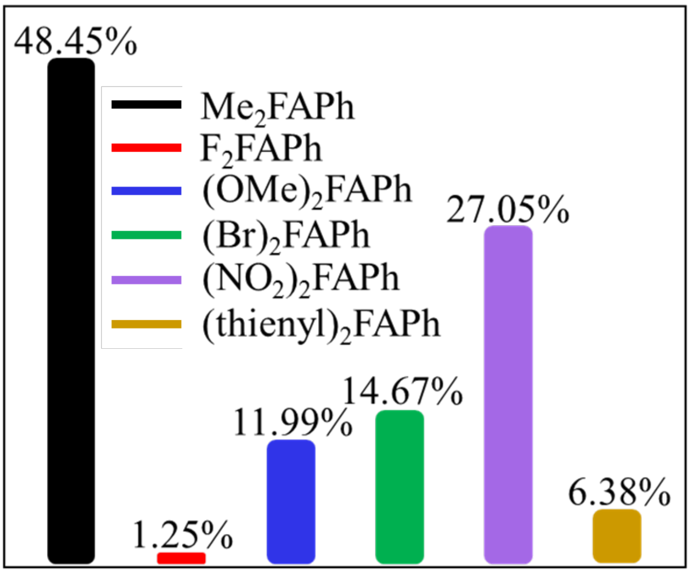

Supplementary Figure 54. Fluorescence quantum yields of **5a**, **5b**, **5c**, **5d**, **5e**, and, **5f** were 25.9, 13.7, 7.1, 2.0, 16.2, and 9.9%, respectively. (relative quantum yields were measured by using anthracene as standard)

6. X-ray Structure and Structure Analysis

6.1. X-ray single crystal structure of FAs (5a)

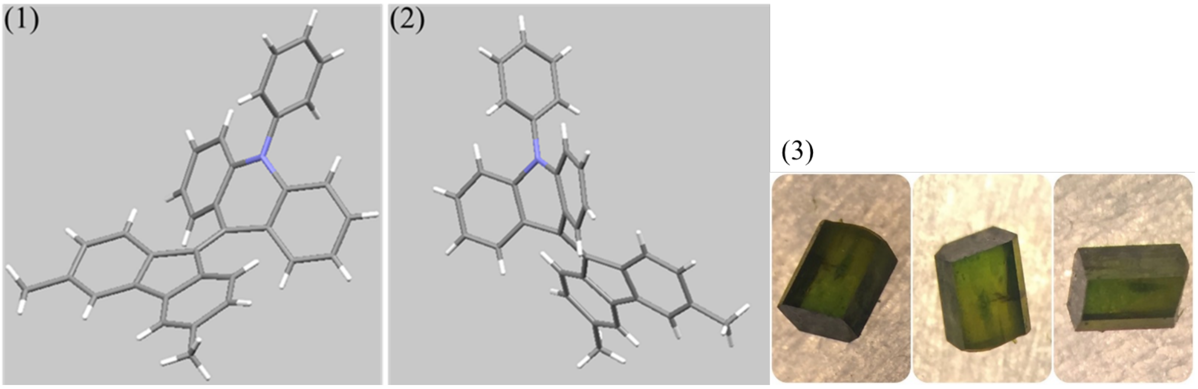

Supplementary Figure 55. Single crystal X-ray structures with capped sticks models of Me_2FAPh (**5a)** (folded conformer). (1) Top view of folded **5a**, (2) side view of folded **5a**, (3) microscopy photograph of folded **5a** crystal. Single crystals were obtained from slow evaporation of petroleum ether solution.

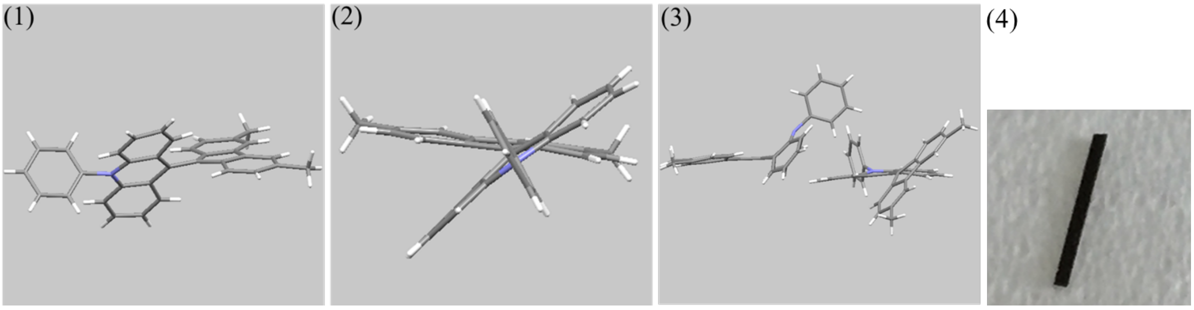

Supplementary Figure 56. Single crystal X-ray structures with capped sticks models of Me_2FAPh (**5a**) (mixture of the twisted and folded conformer). (1), Side view of twisted **5a**. (2), Top view of twisted **5a**. (3), Structure of both folded and twisted **5a**. (4), Photograph of **5a** crystal (mixture of folded and twisted 1:1). The folded structure in (1) and (2) is hidden for clarity. Single crystals were obtained from slow evaporation of a CH_2Cl_2/petroleum ether solution.

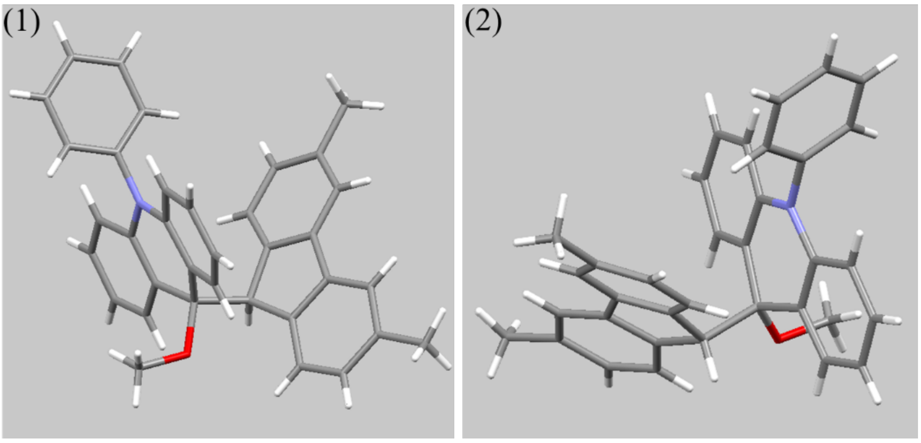

Supplementary Figure 57. Single crystal X-ray structures with capped sticks models of (**6a)**. Single crystals were obtained from slow evaporation of methanol solution.

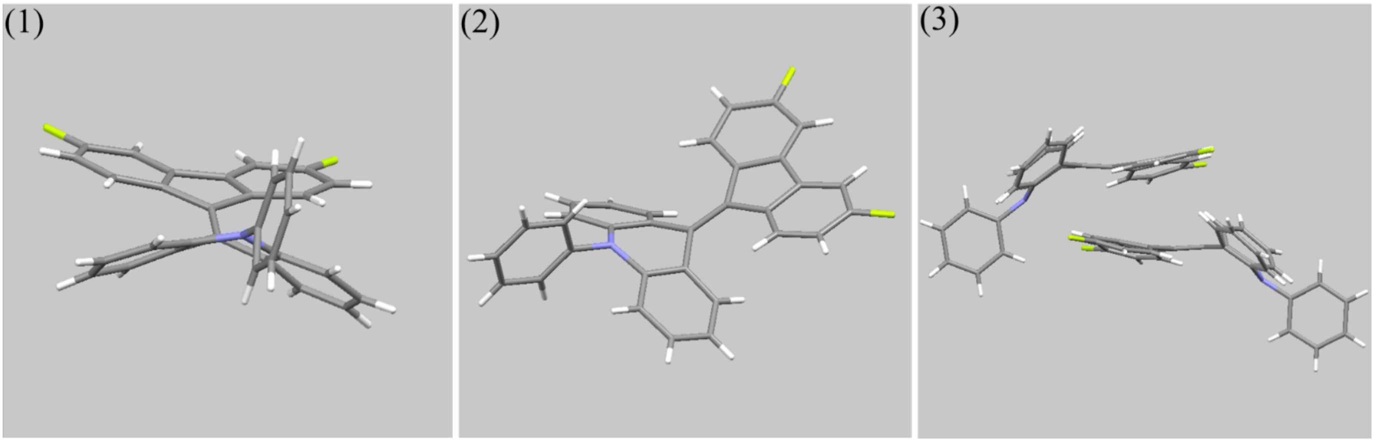

Supplementary Figure 58. Single crystal X-ray structures with capped sticks models of **5b** (folded conformer). Single crystals were obtained from slow evaporation of petroleum ether solution.

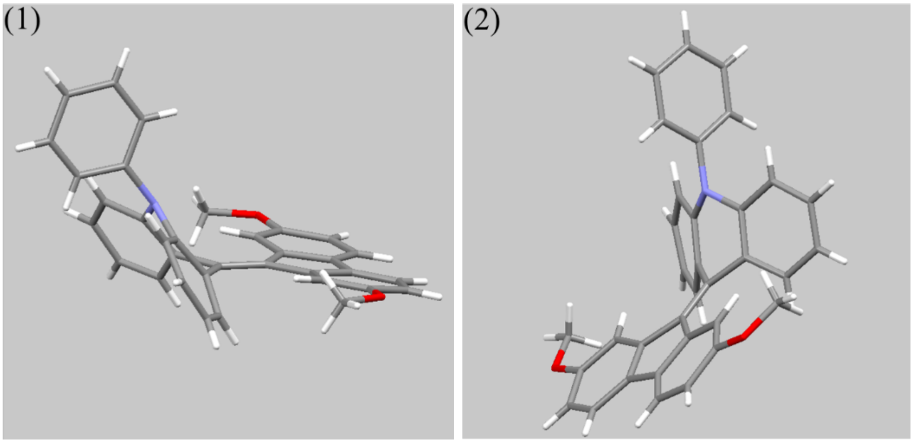

Supplementary Figure 59. Single crystal X-ray structures with capped sticks models of **5c** (folded conformer). Single crystals were obtained from slow evaporation of petroleum ether solution.

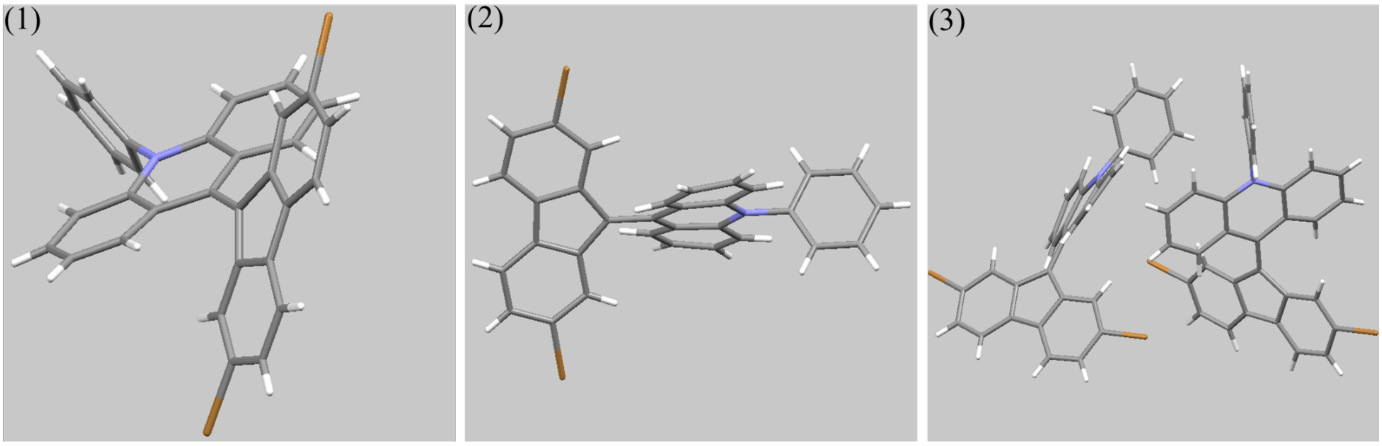

Supplementary Figure 60. Single crystal X-ray structures with capped sticks models of **5d** (twisted conformer). Single crystals were obtained from slow evaporation of CH_2Cl_2/petroleum ether solution.

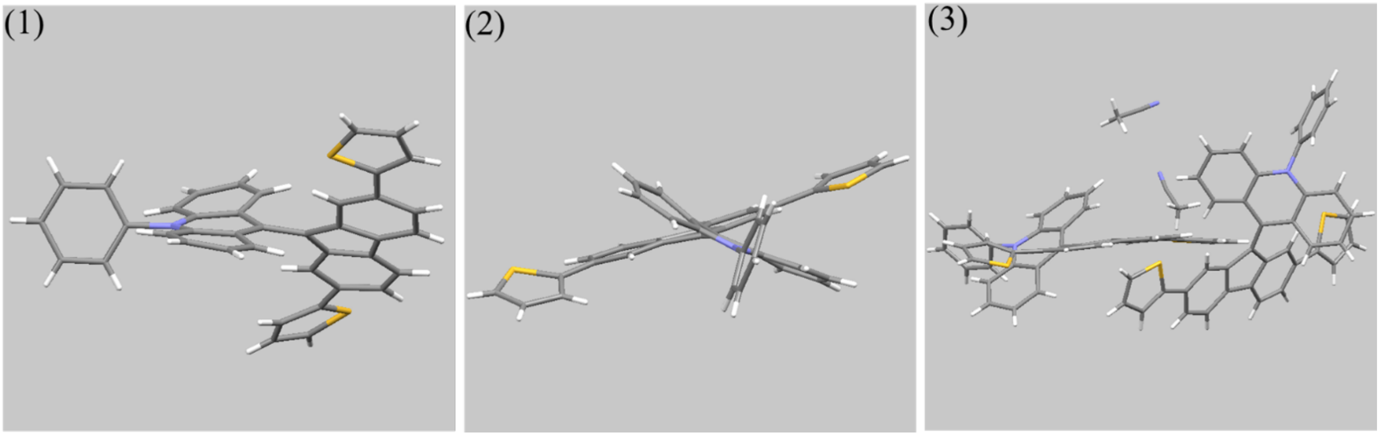

Supplementary Figure 61. Single crystal X-ray structures with capped sticks models of **5e** (twisted conformer). Single crystals were obtained from slow evaporation of a MeCN/petroleum ether solution.

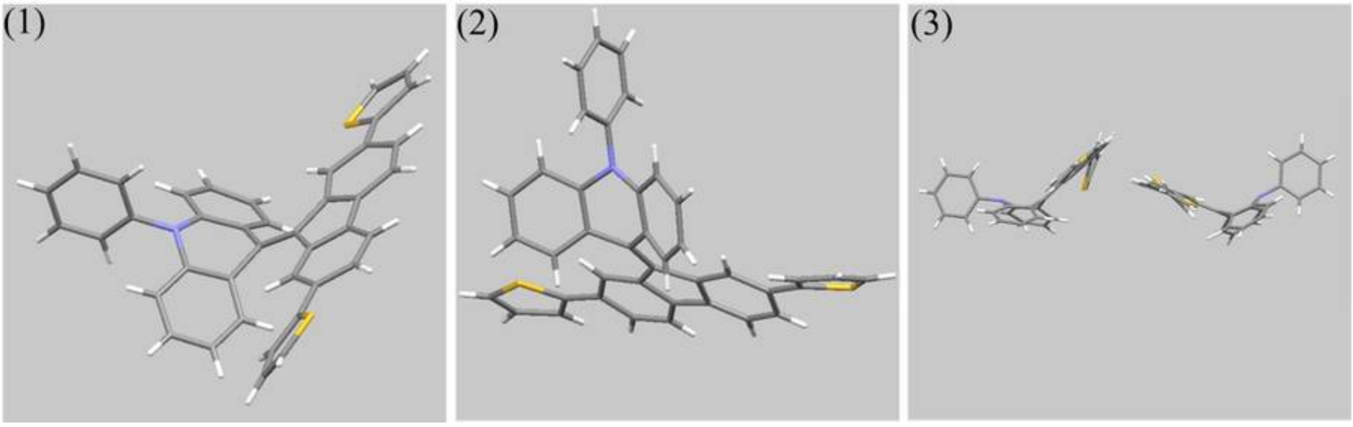

Supplementary Figure 62. Single crystal X-ray structures with capped sticks models of **5e** (folded conformer). Single crystals were obtained from slow evaporation of petroleum ether solution.

Supplementary Note 3

The absolute structures of the FAs, except for **5f**, were determined by single-crystal XRD. Crystal structures of both the folded and twisted conformers were obtained, providing structural information for the FAs. For **5a** and **5e** in particular, both the folded and twisted conformers were present in a unit cell in a 1:1 ratio. These are the first examples of BAEs containing both conformers in one crystal. The structure of methanol adduct **6a** was also unambiguously determined. The pure ethylenic twist angle (ω) around C9=C9' was defined as the average of the two torsion angles C9a–C9–C9'–C9a' and C8a–C9–C9'–C8a'. Folding dihedral angles of the tricyclic fluorene moiety (A–C) and the tricyclic acridane moiety (D–F) were defined as the dihedral angles between the least-square planes of the atoms in the terminal benzene rings of each tricyclic moiety. The bistricyclic dihedral angle between two tricyclic moieties (ABC–DEF) was defined as the folding dihedral angle between the least-square planes of all carbon and nitrogen atoms in ABC and those in DEF for the folded conformer. There are two well-known ways for overcrowded BAEs to alleviate steric hindrance in the fjord area. One way is twisting, and the other is folding. Twisting generates ω. As is shown in Supplementray Table 1, the two moieties on each side of the central double bond were twisted by ω = 40–57°. In the case of twisted **5e**, ω was high at 56.7°, showing considerable deformation from the classical planar ethylene structure. It is worth noting that both moieties in the twisted conformer had remarkable planarity and the conjugation was not broken. Folding generates dihedral angles included two types: (1) within the fluorene moiety (A–C) and the acridane moiety (D–F) and (2) between the fluorene and acridane moieties (ABC–DEF).

For the folded structures of **5c** and **5e**, the dihedral angles between D and F were high at 46.06° and 45.49°, respectively. In addition, the folding angle of A–C was typically bigger than that of D–F, implying that folding of the acridane moiety contributed more to alleviating steric hindrance at higher degrees of folding. This also means that the planar fluorene was relatively rigid. By folding of the acridane moiety, the steric hindrance in the fjord region is further alleviated.

7. Crystal Data Collection Parameters

Supplementary Table 1. Crystal data and structure refinement for Me_2FAPh (**5a**) (folded).

Empirical formula	$C_{34}H_{25}N$
Formula weight	447.55
Temperature	295.15 K
Wavelength	0.71073 Å
Crystal system	Monoclinic
Space group	$P1_{21}/c1$
a = 8.2932(9) Å	α= 90°.
b = 28.767(4) Å	β= 110.253(4)°.
c = 10.6831(14) Å	γ= 90°.
Volume	2391.1(5) Å3
Z	4
Density (calculated)	1.243 mg/m^3
Absorption coefficient	0.071 mm^{-1}
F(000)	944
Theta range for data collection	2.477 to 27.446°.
Index ranges	-10 <= h <= 10, -37 <= k <= 37, -10 <= l <= 13
Reflections collected	21201
Independent reflections	5413 [R(int) = 0.0332]
Completeness to theta = 25.242°	99.30%
Absorption correction	Semi-empirical from equivalents
Max. and min. transmission	0.7455 and 0.6454
Refinement method	Full-matrix least-squares on F^2
Data / restraints / parameters	5413 / 0 / 318
Goodness-of-fit on F^2	1.021
Final R indices [I>2sigma(I)]	R_1 = 0.0499, wR_2 = 0.1192
R indices (all data)	R_1 = 0.0792, wR_2 = 0.1324
Extinction coefficient	n/a
Largest diff. peak and hole	0.222 and -0.187 e.Å^{-3}

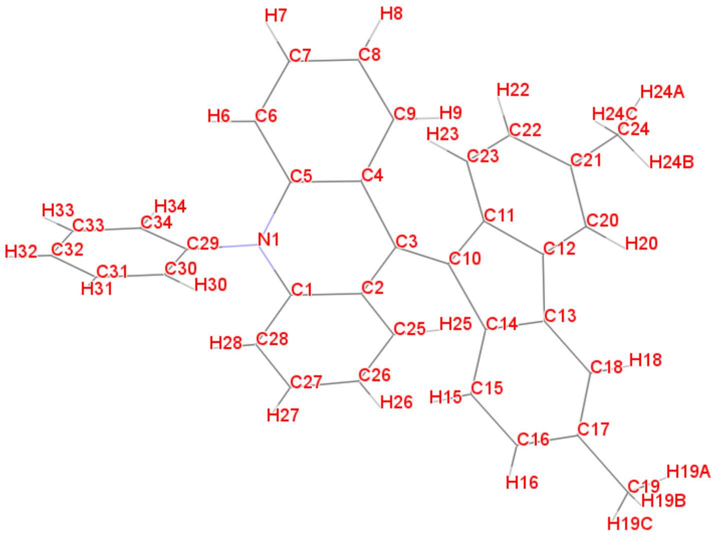

Supplementary Table 2. Bond lengths for Me_2FAPh (**5a**) (folded).

N(1)-C(1)	1.4091(19)	C(11)-C(23)	1.396(2)	C(23)-H(23)	0.93
N(1)-C(5)	1.4058(18)	C(12)-C(13)	1.4583(19)	C(24)-H(24A)	0.96
N(1)-C(29)	1.4336(18)	C(12)-C(20)	1.383(2)	C(24)-H(24B)	0.96
C(1)-C(2)	1.400(2)	C(13)-C(14)	1.404(2)	C(24)-H(24C)	0.96
C(1)-C(28)	1.390(2)	C(13)-C(18)	1.3868(19)	C(25)-H(25)	0.93
C(2)-C(3)	1.476(2)	C(14)-C(15)	1.391(2)	C(25)-C(26)	1.368(3)
C(2)-C(25)	1.393(2)	C(15)-H(15)	0.93	C(26)-H(26)	0.93
C(3)-C(4)	1.478(2)	C(15)-C(16)	1.377(2)	C(26)-C(27)	1.376(3)
C(3)-C(10)	1.360(2)	C(16)-H(16)	0.93	C(27)-H(27)	0.93
C(4)-C(5)	1.403(2)	C(16)-C(17)	1.388(2)	C(27)-C(28)	1.377(3)
C(4)-C(9)	1.392(2)	C(17)-C(18)	1.381(2)	C(28)-H(28)	0.93
C(5)-C(6)	1.396(2)	C(17)-C(19)	1.509(2)	C(29)-C(30)	1.372(2)
C(6)-H(6)	0.93	C(18)-H(18)	0.93	C(29)-C(34)	1.377(2)
C(6)-C(7)	1.372(2)	C(19)-H(19A)	0.96	C(30)-H(30)	0.93
C(7)-H(7)	0.93	C(19)-H(19B)	0.96	C(30)-C(31)	1.386(3)
C(7)-C(8)	1.378(3)	C(19)-H(19C)	0.96	C(31)-H(31)	0.93
C(8)-H(8)	0.93	C(20)-H(20)	0.93	C(31)-C(32)	1.353(3)
C(8)-C(9)	1.372(2)	C(20)-C(21)	1.382(2)	C(32)-H(32)	0.93
C(9)-H(9)	0.93	C(21)-C(22)	1.395(2)	C(32)-C(33)	1.362(3)
C(10)-C(11)	1.493(2)	C(21)-C(24)	1.504(2)	C(33)-H(33)	0.93
C(10)-C(14)	1.4969(19)	C(22)-H(22)	0.93	C(33)-C(34)	1.372(2)
C(11)-C(12)	1.4053(19)	C(22)-C(23)	1.382(2)	C(34)-H(34)	0.93

Symmetry transformations used to generate equivalent atoms.

Supplementary Table 3. Bond angles for Me_2FAPh (**5a**) (folded).

C(1)-N(1)-C(29)	120.1(1)	C(20)-C(12)-C(11)	121.5(1)	C(22)-C(23)-H(23)	120.1
C(5)-N(1)-C(1)	117.0(1)	C(20)-C(12)-C(13)	129.2(1)	C(21)-C(24)-H(24A)	109.5
C(5)-N(1)-C(29)	119.6(1)	C(14)-C(13)-C(12)	108.4(1)	C(21)-C(24)-H(24B)	109.5
C(2)-C(1)-N(1)	118.0(1)	C(18)-C(13)-C(12)	129.6(1)	C(21)-C(24)-H(24C)	109.5
C(28)-C(1)-N(1)	122.2(1)	C(18)-C(13)-C(14)	121.68(1)	H(24A)-C(24)-H(24B)	109.5
C(28)-C(1)-C(2)	119.6(1)	C(13)-C(14)-C(10)	108.8(1)	H(24A)-C(24)-H(24C)	109.5
C(1)-C(2)-C(3)	116.7(1)	C(15)-C(14)-C(10)	133.5(1)	H(24B)-C(24)-H(24C)	109.5
C(25)-C(2)-C(1)	118.2(1)	C(15)-C(14)-C(13)	117.4(1)	C(2)-C(25)-H(25)	119.4
C(25)-C(2)-C(3)	125.0(1)	C(14)-C(15)-H(15)	120	C(26)-C(25)-C(2)	121.2(1)
C(2)-C(3)-C(4)	109.5(1)	C(16)-C(15)-C(14)	119.9(1)	C(26)-C(25)-H(25)	119.4
C(10)-C(3)-C(2)	124.9(1)	C(16)-C(15)-H(15)	120	C(25)-C(26)-H(26)	120.3
C(10)-C(3)-C(4)	125.3(1)	C(15)-C(16)-H(16)	118.8	C(25)-C(26)-C(27)	119.4(1)
C(5)-C(4)-C(3)	117.0(1)	C(15)-C(16)-C(17)	122.3(1)	C(27)-C(26)-H(26)	120.3
C(9)-C(4)-C(3)	124.5(1)	C(17)-C(16)-H(16)	118.8	C(26)-C(27)-H(27)	119.6
C(9)-C(4)-C(5)	118.3(1)	C(16)-C(17)-C(19)	120.2(1)	C(26)-C(27)-C(28)	120.8(1)
C(4)-C(5)-N(1)	118.0(1)	C(18)-C(17)-C(16)	118.1(1)	C(28)-C(27)-H(27)	119.6
C(6)-C(5)-N(1)	122.3(1)	C(18)-C(17)-C(19)	121.6(1)	C(1)-C(28)-H(28)	120.1
C(6)-C(5)-C(4)	119.5(1)	C(13)-C(18)-H(18)	120	C(27)-C(28)-C(1)	119.7(1)
C(5)-C(6)-H(6)	120	C(17)-C(18)-C(13)	119.9(1)	C(27)-C(28)-H(28)	120.1
C(7)-C(6)-C(5)	119.9(1)	C(17)-C(18)-H(18)	120	C(30)-C(29)-N(1)	119.1(1)

C(7)-C(6)-H(6)	120	C(17)-C(19)-H(19A)	109.5	C(30)-C(29)-C(34)	119.0(1)
C(6)-C(7)-H(7)	119.6	C(17)-C(19)-H(19B)	109.5	C(34)-C(29)-N(1)	121.8(1)
C(6)-C(7)-C(8)	120.8(1)	C(17)-C(19)-H(19C)	109.5	C(29)-C(30)-H(30)	120.1
C(8)-C(7)-H(7)	119.6	H(19A)-C(19)-H(19B)	109.5	C(29)-C(30)-C(31)	119.7(1)
C(7)-C(8)-H(8)	120.3	H(19A)-C(19)-H(19C)	109.5	C(31)-C(30)-H(30)	120.1
C(9)-C(8)-C(7)	119.4(1)	H(19B)-C(19)-H(19C)	109.5	C(30)-C(31)-H(31)	119.7
C(9)-C(8)-H(8)	120.3	C(12)-C(20)-H(20)	119.7	C(32)-C(31)-C(30)	120.6(1)
C(4)-C(9)-H(9)	119.3	C(21)-C(20)-C(12)	120.5(1)	C(32)-C(31)-H(31)	119.7
C(8)-C(9)-C(4)	121.4(1)	C(21)-C(20)-H(20)	119.7	C(31)-C(32)-H(32)	120
C(8)-C(9)-H(9)	119.3	C(20)-C(21)-C(22)	117.8(1)	C(31)-C(32)-C(33)	119.9(1)
C(3)-C(10)-C(11)	128.3(1)	C(20)-C(21)-C(24)	120.8(1)	C(33)-C(32)-H(32)	120
C(3)-C(10)-C(14)	127.4(1)	C(22)-C(21)-C(24)	121.3(1)	C(32)-C(33)-H(33)	119.9
C(11)-C(10)-C(14)	104.1(1)	C(21)-C(22)-H(22)	118.8	C(32)-C(33)-C(34)	120.1(1)
C(12)-C(11)-C(10)	108.7(1)	C(23)-C(22)-C(21)	122.3(1)	C(34)-C(33)-H(33)	119.9
C(23)-C(11)-C(10)	133.2(1)	C(23)-C(22)-H(22)	118.8	C(29)-C(34)-H(34)	119.8
C(23)-C(11)-C(12)	117.6(1)	C(11)-C(23)-H(23)	120.1	C(33)-C(34)-C(29)	120.4(1)
C(11)-C(12)-C(13)	108.8(1)	C(22)-C(23)-C(11)	119.77(1)	C(33)-C(34)-H(34)	119.8

Symmetry transformations used to generate equivalent atoms.

Supplementary Table 4. Crystal data and structure refinement for Me_2FAPh (**5a**) (folded & twisted).

Empirical formula	$C_{34}H_{25}N$
Formula weight	447.55
Temperature	100.15 K
Wavelength	0.71073 Å
Crystal system	Triclinic
Space group	P^{-1}
a = 13.2599(13) Å	*α*= 102.706(3)°.
b = 14.2167(14) Å	*β*= 111.272(3)°.
c = 16.9297(15) Å	*γ*= 95.022(3)°.
Volume	2851.6(5) Å3
Z	4
Density (calculated)	1.042 mg/m^3
Absorption coefficient	0.060 mm^{-1}
F(000)	944
Theta range for data collection	1.341 to 27.572°.
Index ranges	-17 <= h <= 17, -18 <= k <= 18, -21 <= l < =21
Reflections collected	44479
Independent reflections	13031 [*R*(int) = 0.0566]
Completeness to theta = 25.242°	99.20%
Absorption correction	Semi-empirical from equivalents
Max. and min. transmission	0.7455 and 0.6903
Refinement method	Full-matrix least-squares on F^2
Data / restraints / parameters	13031 / 0 / 636

Goodness-of-fit on F^2	1.022
Final R indices [I>2sigma(I)]	R_1 = 0.0867, wR_2 = 0.2612
R indices (all data)	R_1 = 0.1383, wR_2 = 0.2996
Extinction coefficient	0.043(6)
Largest diff. peak and hole	0.591 and -0.387 e.Å^{-3}

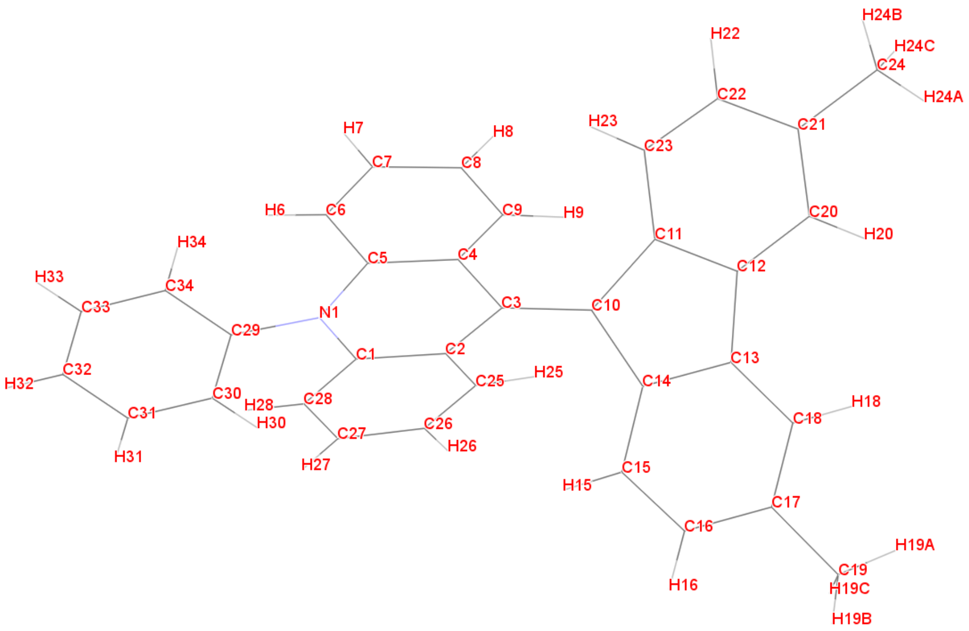

Supplementary Table 5. Bond lengths for Me_2FAPh (**5a**) (twisted).

N(1)-C(1)	1.385(3)	C(11)-C(23)	1.402(4)	C(23)-H(23)	0.95
N(1)-C(5)	1.395(3)	C(12)-C(13)	1.450(5)	C(24)-H(24A)	0.98
N(1)-C(29)	1.440(3)	C(12)-C(20)	1.410(5)	C(24)-H(24B)	0.98
C(1)-C(2)	1.408(3)	C(13)-C(14)	1.420(4)	C(24)-H(24C)	0.98
C(1)-C(28)	1.393(3)	C(13)-C(18)	1.370(4)	C(25)-H(25)	0.95
C(2)-C(3)	1.455(3)	C(14)-C(15)	1.383(4)	C(25)-C(26)	1.366(4)
C(2)-C(25)	1.410(3)	C(15)-H(15)	0.95	C(26)-H(26)	0.95
C(3)-C(4)	1.468(3)	C(15)-C(16)	1.364(4)	C(26)-C(27)	1.382(4)
C(3)-C(10)	1.397(3)	C(16)-H(16)	0.95	C(27)-H(27)	0.95
C(4)-C(5)	1.409(3)	C(16)-C(17)	1.404(5)	C(27)-C(28)	1.373(4)
C(4)-C(9)	1.405(3)	C(17)-C(18)	1.366(5)	C(28)-H(28)	0.95
C(5)-C(6)	1.398(3)	C(17)-C(19)	1.519(5)	C(29)-C(30)	1.376(3)
C(6)-H(6)	0.95	C(18)-H(18)	0.95	C(29)-C(34)	1.381(3)
C(6)-C(7)	1.367(4)	C(19)-H(19A)	0.98	C(30)-H(30)	0.95
C(7)-H(7)	0.95	C(19)-H(19B)	0.98	C(30)-C(31)	1.378(4)
C(7)-C(8)	1.378(4)	C(19)-H(19C)	0.98	C(31)-H(31)	0.95
C(8)-H(8)	0.95	C(20)-H(20)	0.95	C(31)-C(32)	1.369(4)
C(8)-C(9)	1.374(4)	C(20)-C(21)	1.387(6)	C(32)-H(32)	0.95
C(9)-H(9)	0.95	C(21)-C(22)	1.352(6)	C(32)-C(33)	1.357(4)
C(10)-C(11)	1.484(4)	C(21)-C(24)	1.538(6)	C(33)-H(33)	0.95
C(10)-C(14)	1.475(4)	C(22)-H(22)	0.95	C(33)-C(34)	1.381(4)
C(11)-C(12)	1.400(4)	C(22)-C(23)	1.395(5)	C(34)-H(34)	0.95

Supplementary Table 6. Bond angles for Me_2FAPh (**5a**) (twisted).

C(1)-N(1)-C(5)	121.51(18)	C(11)-C(12)-C(20)	121.3(4)	C(22)-C(23)-H(23)	121.1
C(1)-N(1)-C(29)	119.29(19)	C(20)-C(12)-C(13)	129.4(3)	C(21)-C(24)-H(24A)	109.5
C(5)-N(1)-C(29)	119.10(18)	C(14)-C(13)-C(12)	108.1(3)	C(21)-C(24)-H(24B)	109.5
N(1)-C(1)-C(2)	119.5(2)	C(18)-C(13)-C(12)	131.7(3)	C(21)-C(24)-H(24C)	109.5
N(1)-C(1)-C(28)	119.9(2)	C(18)-C(13)-C(14)	120.1(3)	H(24A)-C(24)-H(24B)	109.5
C(28)-C(1)-C(2)	120.5(2)	C(13)-C(14)-C(10)	108.7(3)	H(24A)-C(24)-H(24C)	109.5
C(1)-C(2)-C(3)	121.9(2)	C(15)-C(14)-C(10)	132.6(2)	H(24B)-C(24)-H(24C)	109.5
C(1)-C(2)-C(25)	115.8(2)	C(15)-C(14)-C(13)	118.4(3)	C(2)-C(25)-H(25)	118.4
C(25)-C(2)-C(3)	121.8(2)	C(14)-C(15)-H(15)	119.9	C(26)-C(25)-C(2)	123.1(2)
C(2)-C(3)-C(4)	115.33(19)	C(16)-C(15)-C(14)	120.2(3)	C(26)-C(25)-H(25)	118.4
C(10)-C(3)-C(2)	121.4(2)	C(16)-C(15)-H(15)	119.9	C(25)-C(26)-H(26)	120.4
C(10)-C(3)-C(4)	123.3(2)	C(15)-C(16)-H(16)	119.2	C(25)-C(26)-C(27)	119.3(2)
C(5)-C(4)-C(3)	120.7(2)	C(15)-C(16)-C(17)	121.6(3)	C(27)-C(26)-H(26)	120.4
C(9)-C(4)-C(3)	122.8(2)	C(17)-C(16)-H(16)	119.2	C(26)-C(27)-H(27)	120
C(9)-C(4)-C(5)	116.3(2)	C(16)-C(17)-C(19)	119.4(4)	C(28)-C(27)-C(26)	119.9(2)
N(1)-C(5)-C(4)	120.2(2)	C(18)-C(17)-C(16)	118.1(3)	C(28)-C(27)-H(27)	120
N(1)-C(5)-C(6)	119.5(2)	C(18)-C(17)-C(19)	122.5(3)	C(1)-C(28)-H(28)	119.6
C(6)-C(5)-C(4)	120.3(2)	C(13)-C(18)-H(18)	119.3	C(27)-C(28)-C(1)	120.8(2)
C(5)-C(6)-H(6)	119.7	C(17)-C(18)-C(13)	121.4(3)	C(27)-C(28)-H(28)	119.6
C(7)-C(6)-C(5)	120.6(2)	C(17)-C(18)-H(18)	119.3	C(30)-C(29)-N(1)	119.4(2)
C(7)-C(6)-H(6)	119.7	C(17)-C(19)-H(19A)	109.5	C(30)-C(29)-C(34)	120.2(2)
C(6)-C(7)-H(7)	119.9	C(17)-C(19)-H(19B)	109.5	C(34)-C(29)-N(1)	120.3(2)
C(6)-C(7)-C(8)	120.3(2)	C(17)-C(19)-H(19C)	109.5	C(29)-C(30)-H(30)	120
C(8)-C(7)-H(7)	119.9	H(19A)-C(19)-H(19B)	109.5	C(29)-C(30)-C(31)	120.1(2)
C(7)-C(8)-H(8)	120.3	H(19A)-C(19)-H(19C)	109.5	C(31)-C(30)-H(30)	120
C(9)-C(8)-C(7)	119.5(3)	H(19B)-C(19)-H(19C)	109.5	C(30)-C(31)-H(31)	120.2
C(9)-C(8)-H(8)	120.3	C(12)-C(20)-H(20)	120.6	C(32)-C(31)-C(30)	119.5(2)
C(4)-C(9)-H(9)	118.8	C(21)-C(20)-C(12)	118.9(4)	C(32)-C(31)-H(31)	120.2
C(8)-C(9)-C(4)	122.5(3)	C(21)-C(20)-H(20)	120.6	C(31)-C(32)-H(32)	119.7
C(8)-C(9)-H(9)	118.8	C(20)-C(21)-C(24)	118.0(5)	C(33)-C(32)-C(31)	120.6(2)
C(3)-C(10)-C(11)	129.0(3)	C(22)-C(21)-C(20)	119.1(4)	C(33)-C(32)-H(32)	119.7
C(3)-C(10)-C(14)	125.6(2)	C(22)-C(21)-C(24)	122.6(5)	C(32)-C(33)-H(33)	119.6
C(14)-C(10)-C(11)	105.3(2)	C(21)-C(22)-H(22)	118	C(32)-C(33)-C(34)	120.8(3)
C(12)-C(11)-C(10)	108.6(3)	C(21)-C(22)-C(23)	124.0(4)	C(34)-C(33)-H(33)	119.6
C(12)-C(11)-C(23)	118.8(3)	C(23)-C(22)-H(22)	118	C(29)-C(34)-H(34)	120.6
C(23)-C(11)-C(10)	131.7(3)	C(11)-C(23)-H(23)	121.1	C(33)-C(34)-C(29)	118.8(3)
C(11)-C(12)-C(13)	109.2(2)	C(22)-C(23)-C(11)	117.8(3)	C(33)-C(34)-H(34)	120.6

Supplementary Table 7. Crystal data and structure refinement for F_2FAPh (**5b**) (folded).

Empirical formula	$C_{32}H_{19}F_2N$
Formula weight	455.48
Temperature	273.15 K
Wavelength	0.71073 Å
Crystal system	Monoclinic
Space group	$P12_1/c1$

a = 9.0332(5) Å	α = 90°.
b = 16.9454(10) Å	β = 90°.
c = 29.1518(16) Å	γ = 90°.
Volume	4462.3(4) $Å^3$
Z	8
Density (calculated)	1.356 mg/m^3
Absorption coefficient	0.090 mm^{-1}
F(000)	1888
Theta range for data collection	0.698 to 27.644°.
Index ranges	-11 <= h <= 9, -22 <= k <= 21, -36 <= l <= 37
Reflections collected	47027
Independent reflections	10248 [R(int) = 0.0544]
Completeness to theta = 25.242°	99.90%
Absorption correction	Semi-empirical from equivalents
Max. and min. transmission	0.7456 and 0.7011
Refinement method	Full-matrix least-squares on F^2
Data / restraints / parameters	10248 / 0 / 633
Goodness-of-fit on F^2	0.992
Final R indices [I>2sigma(I)]	R_1 = 0.0517, wR_2 = 0.1165
R indices (all data)	R_1 = 0.1040, wR_2 = 0.1343
Extinction coefficient	0.0079(8)
Largest diff. peak and hole	0.217 and -0.155 e.$Å^{-3}$

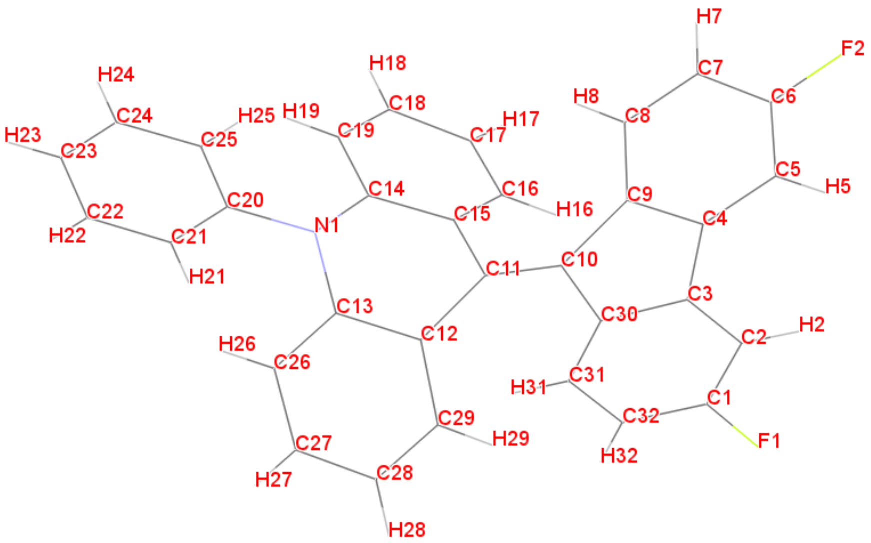

Supplementary Table 8. Bond lengths for F_2FAPh (**5b**) (folded).

F(1)-C(1)	1.357(2)	C(5)-C(4)	1.384(3)	C(16)-H(16)	0.93
F(2)-C(6)	1.362(3)	C(5)-C(6)	1.368(3)	C(2)-H(2)	0.93
N(1)-C(20)	1.442(2)	C(15)-C(14)	1.403(3)	C(21)-H(21)	0.93
N(1)-C(14)	1.404(3)	C(15)-C(16)	1.394(3)	C(21)-C(22)	1.377(3)
N(1)-C(13)	1.406(2)	C(20)-C(21)	1.382(3)	C(7)-H(7)	0.93
C(17)-H(17)	0.93	C(20)-C(25)	1.370(3)	C(7)-C(6)	1.365(3)
C(17)-C(16)	1.370(3)	C(31)-H(31)	0.93	C(28)-H(28)	0.93

C(17)-C(18)	1.382(3)	C(31)-C(32)	1.390(3)	C(28)-C(27)	1.365(3)
C(12)-C(11)	1.481(3)	C(14)-C(19)	1.398(3)	C(18)-H(18)	0.93
C(12)-C(13)	1.406(3)	C(3)-C(4)	1.452(3)	C(25)-H(25)	0.93
C(12)-C(29)	1.389(3)	C(3)-C(2)	1.391(3)	C(25)-C(24)	1.388(3)
C(11)-C(10)	1.367(3)	C(13)-C(26)	1.402(3)	C(32)-H(32)	0.93
C(11)-C(15)	1.480(3)	C(1)-C(2)	1.369(3)	C(26)-H(26)	0.93
C(10)-C(30)	1.499(3)	C(1)-C(32)	1.368(3)	C(26)-C(27)	1.372(3)
C(10)-C(9)	1.495(3)	C(19)-H(19)	0.93	C(22)-H(22)	0.93
C(30)-C(31)	1.390(3)	C(19)-C(18)	1.372(3)	C(22)-C(23)	1.368(4)
C(30)-C(3)	1.413(3)	C(29)-H(29)	0.93	C(27)-H(27)	0.93
C(9)-C(4)	1.407(3)	C(29)-C(28)	1.376(3)	C(24)-H(24)	0.93
C(9)-C(8)	1.385(3)	C(8)-H(8)	0.93	C(24)-C(23)	1.358(4)
C(5)-H(5)	0.93	C(8)-C(7)	1.388(3)	C(23)-H(23)	0.93

Supplementary Table 9. Bond angles for F_2FAPh (**5b**) (folded).

C(14)-N(1)-C(20)	118.18(16)	C(15)-C(14)-N(1)	119.05(17)	C(8)-C(7)-H(7)	120.2
C(14)-N(1)-C(13)	117.47(16)	C(19)-C(14)-N(1)	121.15(18)	C(6)-C(7)-C(8)	119.6(2)
C(13)-N(1)-C(20)	118.54(16)	C(19)-C(14)-C(15)	119.79(19)	C(6)-C(7)-H(7)	120.2
C(16)-C(17)-H(17)	120.2	C(30)-C(3)-C(4)	108.47(17)	C(29)-C(28)-H(28)	120.3
C(16)-C(17)-C(18)	119.6(2)	C(2)-C(3)-C(30)	121.7(2)	C(27)-C(28)-C(29)	119.4(2)
C(18)-C(17)-H(17)	120.2	C(2)-C(3)-C(4)	129.53(19)	C(27)-C(28)-H(28)	120.3
C(13)-C(12)-C(11)	117.29(17)	N(1)-C(13)-C(12)	119.25(17)	C(17)-C(18)-H(18)	119.8
C(29)-C(12)-C(11)	124.67(18)	C(26)-C(13)-N(1)	121.55(18)	C(19)-C(18)-C(17)	120.4(2)
C(29)-C(12)-C(13)	118.02(18)	C(26)-C(13)-C(12)	119.19(18)	C(19)-C(18)-H(18)	119.8
C(10)-C(11)-C(12)	125.25(18)	C(9)-C(4)-C(3)	109.02(17)	C(20)-C(25)-H(25)	120.4
C(10)-C(11)-C(15)	124.70(18)	C(5)-C(4)-C(9)	121.8(2)	C(20)-C(25)-C(24)	119.3(2)
C(15)-C(11)-C(12)	109.98(15)	C(5)-C(4)-C(3)	128.96(19)	C(24)-C(25)-H(25)	120.4
C(11)-C(10)-C(30)	128.61(18)	F(1)-C(1)-C(2)	118.3(2)	C(31)-C(32)-H(32)	120.4
C(11)-C(10)-C(9)	127.26(18)	F(1)-C(1)-C(32)	118.6(2)	C(1)-C(32)-C(31)	119.2(2)
C(9)-C(10)-C(30)	103.96(15)	C(32)-C(1)-C(2)	123.0(2)	C(1)-C(32)-H(32)	120.4
C(31)-C(30)-C(10)	133.57(19)	C(14)-C(19)-H(19)	119.9	C(13)-C(26)-H(26)	120.1
C(31)-C(30)-C(3)	117.72(18)	C(18)-C(19)-C(14)	120.1(2)	C(27)-C(26)-C(13)	119.8(2)
C(3)-C(30)-C(10)	108.57(18)	C(18)-C(19)-H(19)	119.9	C(27)-C(26)-H(26)	120.1
C(4)-C(9)-C(10)	108.78(18)	C(12)-C(29)-H(29)	119.2	F(2)-C(6)-C(5)	118.6(2)
C(8)-C(9)-C(10)	132.65(18)	C(28)-C(29)-C(12)	121.5(2)	F(2)-C(6)-C(7)	118.4(2)
C(8)-C(9)-C(4)	118.33(19)	C(28)-C(29)-H(29)	119.2	C(7)-C(6)-C(5)	123.0(2)
C(4)-C(5)-H(5)	121.4	C(9)-C(8)-H(8)	120.1	C(21)-C(22)-H(22)	119.9
C(6)-C(5)-H(5)	121.4	C(9)-C(8)-C(7)	119.8(2)	C(23)-C(22)-C(21)	120.1(2)
C(6)-C(5)-C(4)	117.2(2)	C(7)-C(8)-H(8)	120.1	C(23)-C(22)-H(22)	119.9
C(14)-C(15)-C(11)	117.87(17)	C(17)-C(16)-C(15)	121.7(2)	C(28)-C(27)-C(26)	121.1(2)
C(16)-C(15)-C(11)	124.30(18)	C(17)-C(16)-H(16)	119.1	C(28)-C(27)-H(27)	119.4
C(16)-C(15)-C(14)	117.72(19)	C(15)-C(16)-H(16)	119.1	C(26)-C(27)-H(27)	119.4
C(21)-C(20)-N(1)	120.21(19)	C(3)-C(2)-H(2)	121.3	C(25)-C(24)-H(24)	119.9
C(25)-C(20)-N(1)	119.37(18)	C(1)-C(2)-C(3)	117.4(2)	C(23)-C(24)-C(25)	120.3(2)
C(25)-C(20)-C(21)	120.4(2)	C(1)-C(2)-H(2)	121.3	C(23)-C(24)-H(24)	119.9

C(30)-C(31)-H(31)	119.7	C(20)-C(21)-H(21)	120.3	C(22)-C(23)-H(23)	119.7
C(32)-C(31)-C(30)	120.6(2)	C(22)-C(21)-C(20)	119.4(2)	C(24)-C(23)-C(22)	120.5(2)
C(32)-C(31)-H(31)	119.7	C(22)-C(21)-H(21)	120.3	C(24)-C(23)-H(23)	119.7

Supplementary Table 10. Crystal data and structure refinement for $(OMe)_2$FAPh (**5c**) (folded).

Empirical formula	$C_{34}H_{25}NO_2$
Formula weight	479.55
Temperature	273.15 K
Wavelength	0.71073 Å
Crystal system	Orthorhombic
Space group	Pmn21
a = 14.5574(10) Å	α = 90°.
b = 10.7889(6) Å	β = 90°.
c = 8.0288(5) Å	γ = 90°.
Volume	1260.99(14) Å^3
Z	2
Density (calculated)	1.263 mg/m^3
Absorption coefficient	0.078 mm^{-1}
F(000)	504
Theta range for data collection	2.350 to 27.504°.
Index ranges	-18 <= h <= 14, -13 <= k <= 14, -10 <= l <= 10
Reflections collected	12012
Independent reflections	2972 [R(int) = 0.0424]
Completeness to theta = 25.242°	99.10%
Absorption correction	Semi-empirical from equivalents
Max. and min. transmission	0.7456 and 0.5638
Refinement method	Full-matrix least-squares on F^2
Data / restraints / parameters	2972 / 1 / 183
Goodness-of-fit on F^2	1.01
Final R indices [I>2sigma(I)]	R_1 = 0.0629, wR_2 = 0.1536
R indices (all data)	R_1 = 0.0902, wR_2 = 0.1705
Absolute structure parameter	-1(5)
Extinction coefficient	*n/a*
Largest diff. peak and hole	0.386 and -0.223 e.Å^{-3}

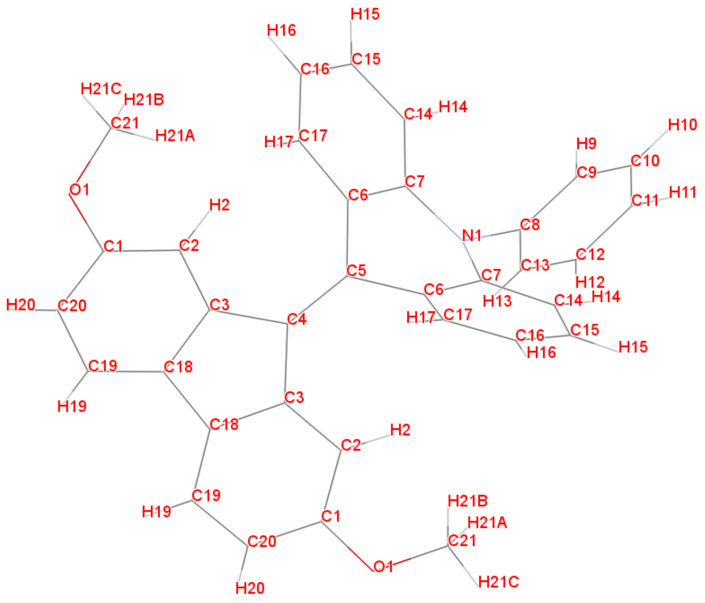

Supplementary Table 11. Bond lengths for (OMe)$_2$FAPh (**5c**) (folded).

O(1)-C(1)	1.370(4)	C(10)-H(10)	0.93
O(1)-C(21)	1.413(6)	C(10)-C(11)	1.357(11)
N(1)-C(7)#1	1.411(4)	C(11)-H(11)	0.93
N(1)-C(7)	1.411(4)	C(11)-C(12)	1.397(11)
N(1)-C(8)	1.441(6)	C(12)-H(12)	0.93
C(1)-C(2)	1.387(5)	C(12)-C(13)	1.378(10)
C(1)-C(20)	1.375(6)	C(13)-H(13)	0.93
C(2)-H(2)	0.93	C(14)-H(14)	0.93
C(2)-C(3)	1.387(4)	C(14)-C(15)	1.390(6)
C(3)-C(4)	1.497(3)	C(15)-H(15)	0.93
C(3)-C(18)	1.406(4)	C(15)-C(16)	1.364(6)
C(4)-C(3)#1	1.497(3)	C(16)-H(16)	0.93
C(4)-C(5)	1.353(5)	C(16)-C(17)	1.366(5)
C(5)-C(6)	1.481(3)	C(17)-H(17)	0.93
C(5)-C(6)#1	1.481(3)	C(18)-C(18)#1	1.463(6)
C(6)-C(7)	1.388(4)	C(18)-C(19)	1.380(4)
C(6)-C(17)	1.399(5)	C(19)-H(19)	0.93
C(7)-C(14)	1.394(4)	C(19)-C(20)	1.380(5)
C(8)-C(9)	1.381(7)	C(20)-H(20)	0.93
C(8)-C(13)	1.365(8)	C(21)-H(21A)	0.96
C(9)-H(9)	0.93	C(21)-H(21B)	0.96
C(9)-C(10)	1.344(8)	C(21)-H(21C)	0.96

Supplementary Table 12. Bond angles for (OMe)$_2$FAPh (**5c**) (folded).

C(1)-O(1)-C(21)	118.2(3)	C(10)-C(11)-C(12)	120.0(6)

C(7)#1-N(1)-C(7)	116.1(4)	C(12)-C(11)-H(11)	120
C(7)#1-N(1)-C(8)	118.49(19)	C(11)-C(12)-H(12)	120.8
C(7)-N(1)-C(8)	118.48(19)	C(13)-C(12)-C(11)	118.5(6)
O(1)-C(1)-C(2)	122.6(4)	C(13)-C(12)-H(12)	120.8
O(1)-C(1)-C(20)	115.9(3)	C(8)-C(13)-C(12)	121.1(6)
C(20)-C(1)-C(2)	121.5(3)	C(8)-C(13)-H(13)	119.4
C(1)-C(2)-H(2)	120.5	C(12)-C(13)-H(13)	119.4
C(1)-C(2)-C(3)	118.9(3)	C(7)-C(14)-H(14)	120.3
C(3)-C(2)-H(2)	120.5	C(15)-C(14)-C(7)	119.4(4)
C(2)-C(3)-C(4)	131.4(3)	C(15)-C(14)-H(14)	120.3
C(2)-C(3)-C(18)	119.4(3)	C(14)-C(15)-H(15)	119.6
C(18)-C(3)-C(4)	109.1(2)	C(16)-C(15)-C(14)	120.8(3)
C(3)-C(4)-C(3)#1	104.2(3)	C(16)-C(15)-H(15)	119.6
C(5)-C(4)-C(3)#1	127.92(15)	C(15)-C(16)-H(16)	120
C(5)-C(4)-C(3)	127.92(15)	C(15)-C(16)-C(17)	120.0(3)
C(4)-C(5)-C(6)	125.58(15)	C(17)-C(16)-H(16)	120
C(4)-C(5)-C(6)#1	125.58(15)	C(6)-C(17)-H(17)	119.7
C(6)#1-C(5)-C(6)	108.5(3)	C(16)-C(17)-C(6)	120.5(3)
C(7)-C(6)-C(5)	115.8(3)	C(16)-C(17)-H(17)	119.7
C(7)-C(6)-C(17)	119.1(3)	C(3)-C(18)-C(18)#1	108.64(16)
C(17)-C(6)-C(5)	125.0(3)	C(19)-C(18)-C(3)	120.3(3)
C(6)-C(7)-N(1)	117.8(3)	C(19)-C(18)-C(18)#1	131.01(19)
C(6)-C(7)-C(14)	119.2(3)	C(18)-C(19)-H(19)	120.1
C(14)-C(7)-N(1)	123.1(3)	C(20)-C(19)-C(18)	119.8(3)
C(9)-C(8)-N(1)	120.2(4)	C(20)-C(19)-H(19)	120.1
C(13)-C(8)-N(1)	121.3(5)	C(1)-C(20)-C(19)	119.8(3)
C(13)-C(8)-C(9)	118.5(5)	C(1)-C(20)-H(20)	120.1
C(8)-C(9)-H(9)	119.3	C(19)-C(20)-H(20)	120.1
C(10)-C(9)-C(8)	121.4(6)	O(1)-C(21)-H(21A)	109.5
C(10)-C(9)-H(9)	119.3	O(1)-C(21)-H(21B)	109.5
C(9)-C(10)-H(10)	119.8	O(1)-C(21)-H(21C)	109.5
C(9)-C(10)-C(11)	120.4(6)	H(21A)-C(21)-H(21B)	109.5
C(11)-C(10)-H(10)	119.8	H(21A)-C(21)-H(21C)	109.5
C(10)-C(11)-H(11)	120	H(21B)-C(21)-H(21C)	109.5

Supplementary Table 13. Crystal data and structure refinement for Br_2FAPh (**5d**) (twisted).

Empirical formula	$C_{32}H_{19}Br_2N$
Formula weight	577.30
Temperature/K	291(2)
Crystal system	triclinic
Space group	P-1
a/Å	11.9941(2)
b/Å	15.1105(4)
c/Å	15.6234(4)
α/°	114.137(2)

β/°	94.783(2)
γ/°	105.031(2)
Volume/Å^3	2437.24(11)
Z	4
ρ_{calc}g/cm^3	1.573
μ/mm^{-1}	4.376
F(000)	1152.0
Crystal size/mm^3	0.250 × 0.220 × 0.210
Radiation	CuKα (λ = 1.54184)
2Θ range for data collection/°	6.772 to 142.722
Index ranges	$-14 \le h \le 9$, $-17 \le k \le 18$, $-18 \le l \le 19$
Reflections collected	16527
Independent reflections	9183 [R_{int} = 0.0261, R_{sigma} = 0.0306]
Data/restraints/parameters	9183/0/631
Goodness-of-fit on F^2	1.048
Final R indexes [I >= 2σ (I)]	R_1 = 0.0449, wR_2 = 0.1202
Final R indexes [all data]	R_1 = 0.0500, wR_2 = 0.1257
Largest diff. peak/hole / e Å^{-3}	0.88/-1.04

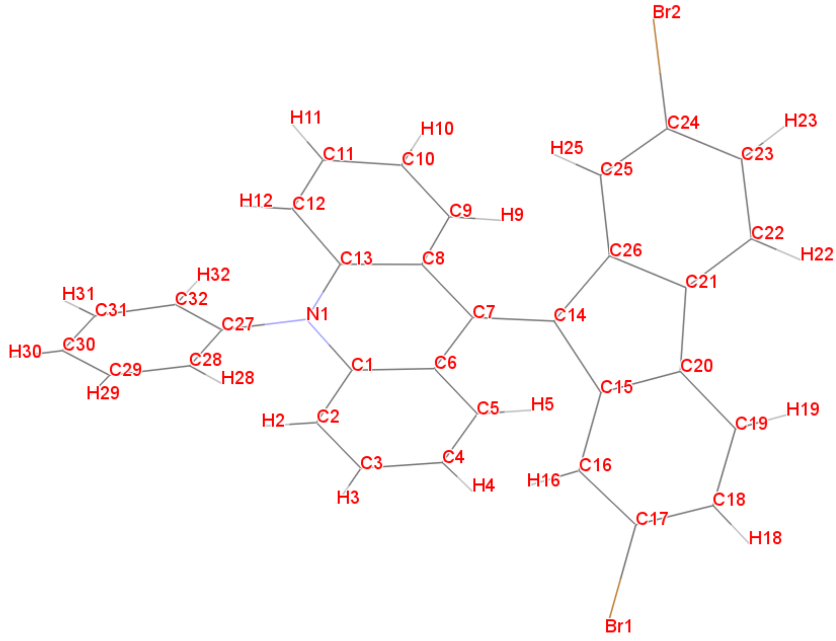

Supplementary Table 14. Bond lengths for Br_2FAPh (**5d**) (twisted).

Atom	Atom	Length/Å	Atom	Atom	Length/Å	Atom	Atom	Length/Å
Br4	C56	1.909(3)	C34	C35	1.366(4)	C46	C47	1.469(4)
Br3	C49	1.908(3)	C16	C17	1.380(4)	C14	C15	1.466(4)
Br1	C17	1.912(3)	C4	C3	1.397(4)	C14	C26	1.471(4)
Br2	C24	1.904(4)	C51	C50	1.379(5)	C40	C41	1.411(4)
N2	C33	1.382(3)	C2	C3	1.367(5)	C40	C45	1.417(3)
N2	C45	1.387(4)	C9	C10	1.375(4)	C58	C57	1.401(4)
N2	C59	1.448(3)	C20	C19	1.397(4)	C52	C51	1.391(4)

N1	C1	1.379(4)	C20	C21	1.437(5)	C52	C47	1.417(3)
N1	C13	1.388(4)	C21	C22	1.397(4)	C54	C55	1.384(4)
N1	C27	1.458(3)	C37	C36	1.374(4)	C26	C25	1.387(4)
C53	C54	1.390(4)	C25	C24	1.398(4)	C26	C21	1.422(4)
C53	C58	1.420(3)	C43	C42	1.396(4)	C45	C44	1.405(4)
C53	C52	1.442(4)	C50	C49	1.390(4)	C13	C12	1.405(4)
C33	C34	1.409(4)	C28	C29	1.384(4)	C47	C48	1.398(4)
C33	C38	1.421(3)	C17	C18	1.378(5)	C59	C64	1.377(4)
C38	C37	1.406(4)	C64	C63	1.391(4)	C59	C60	1.381(4)
C38	C39	1.452(4)	C61	C62	1.371(5)	C1	C2	1.408(4)
C39	C46	1.414(3)	C61	C60	1.387(4)	C15	C16	1.391(4)
C39	C40	1.450(4)	C12	C11	1.372(5)	C15	C20	1.423(4)
C8	C13	1.413(4)	C36	C35	1.402(4)	C5	C4	1.367(4)
C8	C9	1.418(4)	C19	C18	1.370(5)	C41	C42	1.363(4)
C8	C7	1.448(4)	C29	C30	1.373(5)	C57	C56	1.379(4)
C7	C14	1.407(3)	C32	C31	1.394(4)	C44	C43	1.369(4)
C7	C6	1.457(4)	C22	C23	1.367(6)	C27	C28	1.375(4)
C6	C5	1.413(4)	C62	C63	1.380(5)	C27	C32	1.381(4)
C6	C1	1.413(3)	C30	C31	1.374(5)	C55	C56	1.389(4)
C46	C58	1.464(4)	C10	C11	1.390(5)	C48	C49	1.375(4)
C24	C23	1.386(6)						

Supplementary Table 15. Bond angles for Br_2FAPh (**5d**) (twisted).

Atom	Atom	Atom	Angle/°	Atom	Atom	Atom	Angle/°	Atom	Atom	Atom	Angle/°
C33	N2	C45	121.4(2)	C47	C52	C53	108.5(2)	C19	C20	C15	120.2(3)
C33	N2	C59	119.4(2)	C55	C54	C53	119.8(2)	C19	C20	C21	131.4(3)
C45	N2	C59	119.2(2)	C25	C26	C21	119.2(3)	C15	C20	C21	108.1(3)
C1	N1	C13	121.1(2)	C25	C26	C14	132.2(3)	C22	C21	C26	120.9(3)
C1	N1	C27	119.9(2)	C21	C26	C14	108.4(3)	C22	C21	C20	130.3(3)
C13	N1	C27	118.5(2)	N2	C45	C44	119.8(2)	C26	C21	C20	108.8(2)
C54	C53	C58	121.2(3)	N2	C45	C40	120.2(2)	C36	C37	C38	122.8(3)
C54	C53	C52	130.6(2)	C44	C45	C40	120.0(3)	C26	C25	C24	118.0(3)
C58	C53	C52	108.0(2)	N1	C13	C12	119.5(2)	C44	C43	C42	120.2(3)
N2	C33	C34	120.1(2)	N1	C13	C8	120.5(2)	C51	C50	C49	118.8(3)
N2	C33	C38	120.2(2)	C12	C13	C8	120.0(3)	C27	C28	C29	119.3(3)
C34	C33	C38	119.6(2)	C48	C47	C52	118.8(2)	C48	C49	C50	123.3(3)
C37	C38	C33	116.7(2)	C48	C47	C46	132.1(2)	C48	C49	Br3	119.0(2)
C37	C38	C39	122.3(2)	C52	C47	C46	108.7(2)	C50	C49	Br3	117.7(2)
C33	C38	C39	120.7(2)	C64	C59	C60	120.9(3)	C41	C42	C43	119.5(3)
C46	C39	C40	121.8(2)	C64	C59	N2	119.4(2)	C2	C3	C4	120.5(3)
C46	C39	C38	121.9(2)	C60	C59	N2	119.8(2)	C18	C17	C16	123.4(3)
C40	C39	C38	116.4(2)	N1	C1	C2	120.1(2)	C18	C17	Br1	117.9(2)
C13	C8	C9	116.6(3)	N1	C1	C6	120.2(2)	C16	C17	Br1	118.7(3)
C13	C8	C7	120.9(2)	C2	C1	C6	119.7(3)	C59	C64	C63	119.2(3)
C9	C8	C7	122.2(2)	C16	C15	C20	119.0(3)	C62	C61	C60	120.2(3)
C14	C7	C8	123.0(2)	C16	C15	C14	131.2(2)	C59	C60	C61	119.4(3)

C14	C7	C6	121.3(2)	C20	C15	C14	108.9(3)	C11	C12	C13	120.7(3)
C8	C7	C6	115.7(2)	C4	C5	C6	122.7(2)	C37	C36	C35	118.8(3)
C5	C6	C1	116.9(2)	C42	C41	C40	122.6(3)	C34	C35	C36	120.6(3)
C5	C6	C7	121.7(2)	C56	C57	C58	118.6(2)	C18	C19	C20	120.1(3)
C1	C6	C7	121.2(2)	C43	C44	C45	120.6(3)	C30	C29	C28	120.3(3)
C39	C46	C58	127.6(2)	C28	C27	C32	121.3(3)	C19	C18	C17	118.9(3)
C39	C46	C47	126.9(2)	C28	C27	N1	118.3(3)	C27	C32	C31	118.6(3)
C58	C46	C47	105.5(2)	C32	C27	N1	120.4(3)	C23	C22	C21	119.4(3)
C7	C14	C15	126.1(2)	C54	C55	C56	118.6(3)	C61	C62	C63	120.3(3)
C7	C14	C26	128.2(3)	C49	C48	C47	118.4(3)	C29	C30	C31	120.2(3)
C15	C14	C26	105.6(2)	C35	C34	C33	120.7(3)	C9	C10	C11	119.5(3)
C41	C40	C45	116.5(2)	C17	C16	C15	118.4(3)	C12	C11	C10	120.3(3)
C41	C40	C39	122.5(2)	C5	C4	C3	119.1(3)	C30	C31	C32	120.3(3)
C45	C40	C39	120.8(2)	C50	C51	C52	119.6(3)	C23	C24	C25	122.7(4)
C57	C58	C53	118.5(2)	C57	C56	C55	123.3(3)	C23	C24	Br2	119.6(3)
C57	C58	C46	131.8(2)	C57	C56	Br4	119.0(2)	C25	C24	Br2	117.7(3)
C53	C58	C46	109.1(2)	C55	C56	Br4	117.7(2)	C62	C63	C64	120.0(3)
C51	C52	C47	121.1(3)	C3	C2	C1	120.7(3)	C22	C23	C24	119.7(3)
C51	C52	C53	130.4(3)	C10	C9	C8	122.2(3)				

Supplementary Table 16. Crystal data and structure refinement for $(thienyl)_2FAPh$ (**5e**) (folded).

Empirical formula	$C_{40}H_{25}NS_2$
Formula weight	583.73
Temperature	150.0 K
Wavelength	0.71073 Å
Crystal system	Triclinic
Space group	P-1
a = 8.2204(6) Å	α = 92.129(2)°.
b = 10.0096(7) Å	β = 95.467(2)°.
c = 35.337(3) Å	γ = 97.955(2)°.
Volume	2862.8(4) Å3
Z	4
Density (calculated)	1.354 mg/m^3
Absorption coefficient	0.218 mm+
F(000)	1216
Theta range for data collection	2.319 to 26.021°.
Index ranges	-10 <= h <= 10, -12 <= k <= 12, -43 <= l <= 43
Reflections collected	88682
Independent reflections	11237 [R(int) = 0.0635]
Completeness to theta = 25.242°	99.60%
Absorption correction	Semi-empirical from equivalents
Max. and min. transmission	0.7454 and 0.6993
Refinement method	Full-matrix least-squares on F^2
Data / restraints / parameters	11237 / 203 / 879
Goodness-of-fit on F^2	1.069

Final R indices [$I > 2$ sigma(I)]	$R_1 = 0.0630$, $wR_2 = 0.1434$
R indices (all data)	$R_1 = 0.0916$, $wR_2 = 0.1564$
Extinction coefficient	0.0100(7)
Largest diff. peak and hole	0.409 and -0.343 e.Å^{-3}

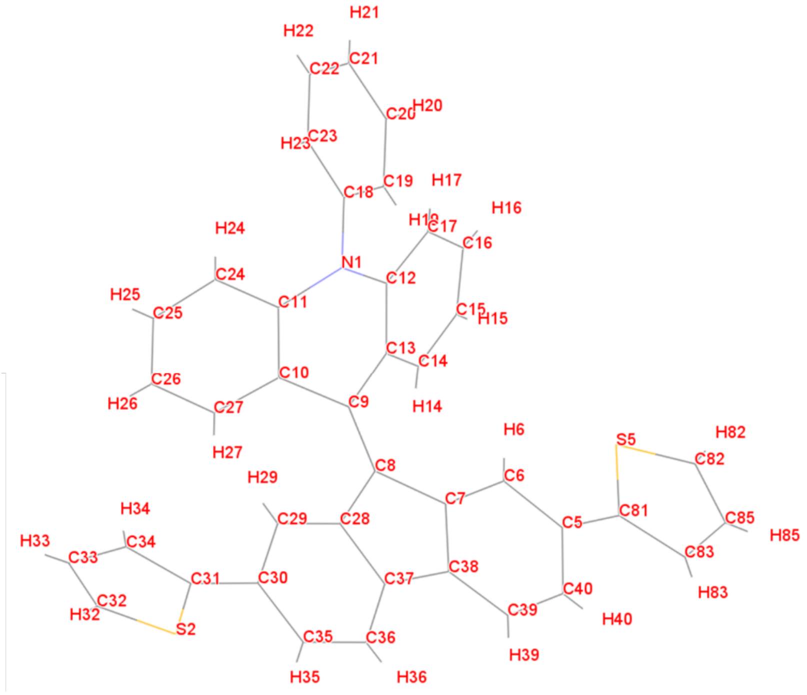

Supplementary Table 17. Bond lengths for (thienyl)$_2$FAPh (**5e**) (folded).

C(32)-S(2)-C(31)	93.8(3)	C(14)-C(13)-C(9)	124.8(3)	C(17)-C(16)-H(16)	119.4
C(12)-N(1)-C(11)	116.5(2)	C(14)-C(13)-C(12)	118.4(3)	C(17)-C(16)-C(15)	121.3(3)
C(12)-N(1)-C(18)	119.1(2)	C(28)-C(29)-H(29)	119.6	C(15)-C(16)-H(16)	119.4
C(11)-N(1)-C(18)	118.7(2)	C(28)-C(29)-C(30)	120.9(3)	C(30)-C(35)-H(35)	119.6
C(31)-C(34)-H(34)	122.6	C(30)-C(29)-H(29)	119.6	C(36)-C(35)-C(30)	120.8(3)
C(31)-C(34)-C(33)	114.9(12)	C(12)-C(17)-H(17)	120.2	C(36)-C(35)-H(35)	119.6
C(33)-C(34)-H(34)	122.6	C(16)-C(17)-C(12)	119.6(3)	C(37)-C(36)-H(36)	120.2
C(11)-C(10)-C(9)	116.8(2)	C(16)-C(17)-H(17)	120.2	C(35)-C(36)-C(37)	119.6(3)
C(27)-C(10)-C(9)	124.5(3)	C(18)-C(23)-H(23)	120	C(35)-C(36)-H(36)	120.2
C(27)-C(10)-C(11)	118.5(3)	C(18)-C(23)-C(22)	120.0(3)	C(14)-C(15)-C(16)	119.1(3)
C(8)-C(9)-C(10)	124.8(3)	C(22)-C(23)-H(23)	120	C(14)-C(15)-H(15)	120.4
C(8)-C(9)-C(13)	126.5(3)	C(29)-C(30)-C(31)	120.2(3)	C(16)-C(15)-H(15)	120.4
C(13)-C(9)-C(10)	108.4(2)	C(35)-C(30)-C(29)	119.3(3)	C(6)-C(5)-C(81A)	103.4(5)
N(1)-C(12)-C(13)	118.0(3)	C(35)-C(30)-C(31)	120.5(3)	C(40)-C(5)-C(6)	120.2(4)
C(17)-C(12)-N(1)	122.4(3)	C(22)-C(21)-H(21)	119.8	C(40)-C(5)-C(81A)	136.1(5)
C(17)-C(12)-C(13)	119.5(3)	C(22)-C(21)-C(20)	120.4(3)	C(81)-C(5)-C(6)	126.2(6)
C(10)-C(11)-N(1)	117.8(2)	C(20)-C(21)-H(21)	119.8	C(81)-C(5)-C(40)	113.6(5)
C(24)-C(11)-N(1)	122.9(2)	C(23)-C(22)-H(22)	120.1	C(38)-C(39)-H(39)	120.1
C(24)-C(11)-C(10)	119.3(3)	C(21)-C(22)-C(23)	119.9(3)	C(40)-C(39)-C(38)	119.9(5)
C(19)-C(18)-N(1)	119.0(3)	C(21)-C(22)-H(22)	120.1	C(40)-C(39)-H(39)	120.1
C(19)-C(18)-C(23)	120.2(3)	C(6)-C(7)-C(8)	133.3(3)	S(2)-C(32)-H(32)	120.1
C(23)-C(18)-N(1)	120.8(3)	C(6)-C(7)-C(38)	118.2(3)	C(33)-C(32)-S(2)	119.8(4)

C(10)-C(27)-H(27)	119.3	C(38)-C(7)-C(8)	108.4(3)	C(33)-C(32)-H(32)	120.1
C(26)-C(27)-C(10)	121.4(3)	C(19)-C(20)-H(20)	120.1	C(33)-C(32)-H(32A)	126.9
C(26)-C(27)-H(27)	119.3	C(21)-C(20)-C(19)	119.8(3)	C(33)-C(32)-C(34A)	106.1(6)
C(11)-C(24)-H(24)	120	C(21)-C(20)-H(20)	120.1	C(34A)-C(32)-H(32A)	126.9
C(25)-C(24)-C(11)	119.9(3)	C(34)-C(31)-S(2)	105.9(8)	C(34)-C(33)-H(33)	127.4
C(25)-C(24)-H(24)	120	C(34)-C(31)-C(30)	130.8(8)	C(32)-C(33)-C(34)	105.2(8)
C(29)-C(28)-C(8)	133.9(3)	C(30)-C(31)-S(2)	123.2(3)	C(32)-C(33)-H(33)	127.4
C(29)-C(28)-C(37)	117.6(3)	C(30)-C(31)-S(2A)	120.6(3)	C(32)-C(33)-H(33A)	121
C(37)-C(28)-C(8)	108.4(3)	C(34A)-C(31)-C(30)	126.5(7)	C(32)-C(33)-S(2A)	117.9(4)
C(18)-C(19)-H(19)	120.1	C(34A)-C(31)-S(2A)	112.9(7)	S(2A)-C(33)-H(33A)	121
C(18)-C(19)-C(20)	119.7(3)	C(7)-C(6)-H(6)	120.3	C(5)-C(40)-H(40)	119.6
C(20)-C(19)-H(19)	120.1	C(7)-C(6)-C(5)	119.4(4)	C(39)-C(40)-C(5)	120.8(4)
C(9)-C(8)-C(28)	127.4(3)	C(5)-C(6)-H(6)	120.3	C(39)-C(40)-H(40)	119.6
C(9)-C(8)-C(7)	128.1(3)	C(13)-C(14)-H(14)	119.4	C(41)-C(42A)-C(44)	115.9(11)
C(28)-C(8)-C(7)	104.5(3)	C(15)-C(14)-C(13)	121.3(3)	C(41)-C(42A)-H(42A)	122.1
C(27)-C(26)-H(26)	120.6	C(15)-C(14)-H(14)	119.4	C(44)-C(42A)-H(42A)	122.1
C(27)-C(26)-C(25)	118.9(3)	C(28)-C(37)-C(38)	109.1(3)	C(43)-S(3A)-C(41)	92.5(2)
C(25)-C(26)-H(26)	120.6	C(36)-C(37)-C(28)	121.8(4)	C(31)-C(34A)-C(32)	110.8(9)
C(24)-C(25)-C(26)	121.1(3)	C(36)-C(37)-C(38)	129.0(3)	C(31)-C(34A)-H(34A)	124.6
C(24)-C(25)-H(25)	119.5	C(7)-C(38)-C(37)	109.4(3)	C(32)-C(34A)-H(34A)	124.6
C(26)-C(25)-H(25)	119.5	C(39)-C(38)-C(7)	121.4(4)	C(33)-S(2A)-C(31)	92.0(3)
C(12)-C(13)-C(9)	116.7(3)	C(39)-C(38)-C(37)	129.2(4)		

Supplementary Table 18. Bond angles for (thienyl)$_2$FAPh (**5e**) (folded).

C(32)-S(2)-C(31)	93.8(3)	C(14)-C(13)-C(9)	124.8(3)	C(17)-C(16)-H(16)	119.4
C(12)-N(1)-C(11)	116.5(2)	C(14)-C(13)-C(12)	118.4(3)	C(17)-C(16)-C(15)	121.3(3)
C(12)-N(1)-C(18)	119.1(2)	C(28)-C(29)-H(29)	119.6	C(15)-C(16)-H(16)	119.4
C(11)-N(1)-C(18)	118.7(2)	C(28)-C(29)-C(30)	120.9(3)	C(30)-C(35)-H(35)	119.6
C(31)-C(34)-H(34)	122.6	C(30)-C(29)-H(29)	119.6	C(36)-C(35)-C(30)	120.8(3)
C(31)-C(34)-C(33)	114.9(12)	C(12)-C(17)-H(17)	120.2	C(36)-C(35)-H(35)	119.6
C(33)-C(34)-H(34)	122.6	C(16)-C(17)-C(12)	119.6(3)	C(37)-C(36)-H(36)	120.2
C(11)-C(10)-C(9)	116.8(2)	C(16)-C(17)-H(17)	120.2	C(35)-C(36)-C(37)	119.6(3)
C(27)-C(10)-C(9)	124.5(3)	C(18)-C(23)-H(23)	120	C(35)-C(36)-H(36)	120.2
C(27)-C(10)-C(11)	118.5(3)	C(18)-C(23)-C(22)	120.0(3)	C(14)-C(15)-C(16)	119.1(3)
C(8)-C(9)-C(10)	124.8(3)	C(22)-C(23)-H(23)	120	C(14)-C(15)-H(15)	120.4
C(8)-C(9)-C(13)	126.5(3)	C(29)-C(30)-C(31)	120.2(3)	C(16)-C(15)-H(15)	120.4
C(13)-C(9)-C(10)	108.4(2)	C(35)-C(30)-C(29)	119.3(3)	C(6)-C(5)-C(81A)	103.4(5)
N(1)-C(12)-C(13)	118.0(3)	C(35)-C(30)-C(31)	120.5(3)	C(40)-C(5)-C(6)	120.2(4)
C(17)-C(12)-N(1)	122.4(3)	C(22)-C(21)-H(21)	119.8	C(40)-C(5)-C(81A)	136.1(5)
C(17)-C(12)-C(13)	119.5(3)	C(22)-C(21)-C(20)	120.4(3)	C(81)-C(5)-C(6)	126.2(6)
C(10)-C(11)-N(1)	117.8(2)	C(20)-C(21)-H(21)	119.8	C(81)-C(5)-C(40)	113.6(5)
C(24)-C(11)-N(1)	122.9(2)	C(23)-C(22)-H(22)	120.1	C(38)-C(39)-H(39)	120.1
C(24)-C(11)-C(10)	119.3(3)	C(21)-C(22)-C(23)	119.9(3)	C(40)-C(39)-C(38)	119.9(5)
C(19)-C(18)-N(1)	119.0(3)	C(21)-C(22)-H(22)	120.1	C(40)-C(39)-H(39)	120.1
C(19)-C(18)-C(23)	120.2(3)	C(6)-C(7)-C(8)	133.3(3)	S(2)-C(32)-H(32)	120.1
C(23)-C(18)-N(1)	120.8(3)	C(6)-C(7)-C(38)	118.2(3)	C(33)-C(32)-S(2)	119.8(4)

C(10)-C(27)-H(27)	119.3	C(38)-C(7)-C(8)	108.4(3)	C(33)-C(32)-H(32)	120.1
C(26)-C(27)-C(10)	121.4(3)	C(19)-C(20)-H(20)	120.1	C(33)-C(32)-H(32A)	126.9
C(26)-C(27)-H(27)	119.3	C(21)-C(20)-C(19)	119.8(3)	C(33)-C(32)-C(34A)	106.1(6)
C(11)-C(24)-H(24)	120	C(21)-C(20)-H(20)	120.1	C(34A)-C(32)-H(32A)	126.9
C(25)-C(24)-C(11)	119.9(3)	C(34)-C(31)-S(2)	105.9(8)	C(34)-C(33)-H(33)	127.4
C(25)-C(24)-H(24)	120	C(34)-C(31)-C(30)	130.8(8)	C(32)-C(33)-C(34)	105.2(8)
C(29)-C(28)-C(8)	133.9(3)	C(30)-C(31)-S(2)	123.2(3)	C(32)-C(33)-H(33)	127.4
C(29)-C(28)-C(37)	117.6(3)	C(30)-C(31)-S(2A)	120.6(3)	C(32)-C(33)-H(33A)	121
C(37)-C(28)-C(8)	108.4(3)	C(34A)-C(31)-C(30)	126.5(7)	C(32)-C(33)-S(2A)	117.9(4)
C(18)-C(19)-H(19)	120.1	C(34A)-C(31)-S(2A)	112.9(7)	S(2A)-C(33)-H(33A)	121
C(18)-C(19)-C(20)	119.7(3)	C(7)-C(6)-H(6)	120.3	C(5)-C(40)-H(40)	119.6
C(20)-C(19)-H(19)	120.1	C(7)-C(6)-C(5)	119.4(4)	C(39)-C(40)-C(5)	120.8(4)
C(9)-C(8)-C(28)	127.4(3)	C(5)-C(6)-H(6)	120.3	C(39)-C(40)-H(40)	119.6
C(9)-C(8)-C(7)	128.1(3)	C(13)-C(14)-H(14)	119.4	C(41)-C(42A)-C(44)	115.9(11)
C(28)-C(8)-C(7)	104.5(3)	C(15)-C(14)-C(13)	121.3(3)	C(41)-C(42A)-H(42A)	122.1
C(27)-C(26)-H(26)	120.6	C(15)-C(14)-H(14)	119.4	C(44)-C(42A)-H(42A)	122.1
C(27)-C(26)-C(25)	118.9(3)	C(28)-C(37)-C(38)	109.1(3)	C(43)-S(3A)-C(41)	92.5(2)
C(25)-C(26)-H(26)	120.6	C(36)-C(37)-C(28)	121.8(4)	C(31)-C(34A)-C(32)	110.8(9)
C(24)-C(25)-C(26)	121.1(3)	C(36)-C(37)-C(38)	129.0(3)	C(31)-C(34A)-H(34A)	124.6
C(24)-C(25)-H(25)	119.5	C(7)-C(38)-C(37)	109.4(3)	C(32)-C(34A)-H(34A)	124.6
C(26)-C(25)-H(25)	119.5	C(39)-C(38)-C(7)	121.4(4)	C(33)-S(2A)-C(31)	92.0(3)
C(12)-C(13)-C(9)	116.7(3)	C(39)-C(38)-C(37)	129.2(4)		

Supplementary Table 19. Crystal data and structure refinement for (thienyl)$_2$FAPh (**5e**) (twisted).

Empirical formula	$C_{40}H_{25}NS_2$
Formula weight	583.73
Temperature/K	273.15
Crystal system	triclinic
Space group	P-1
a/Å	13.6564(11)
b/Å	16.3958(12)
c/Å	16.4632(13)
α/°	87.101(2)
β/°	68.196(2)
γ/°	67.785(2)
Volume/Å^3	3151.1(4)
Z	2
ρ_{calc} g/cm^3	1.277
μ/mm^{-1}	0.201
F(000)	1266
Radiation	MoKα (λ = 0.71073)
2Θ range for data collection/°	2.68 to 55.1
Index ranges	$-17 \leqslant h \leqslant 17, -21 \leqslant k \leqslant 21, -21 \leqslant l \leqslant 21$
Reflections collected	49726
Independent reflections	14483 [Rint = 0.0367, Rsigma = 0.0442]

Data/restraints/parameters	14483/3/370
Goodness-of-fit on F^2	2.163
Final R indexes [$I >= 2\sigma(I)$]	$R_1 = 0.1580$, $wR_2 = 0.4729$
Final R indexes [all data]	$R_1 = 0.1907$, $wR_2 = 0.5034$
Largest diff. peak/hole / e Å^{-3}	4.32/-2.72

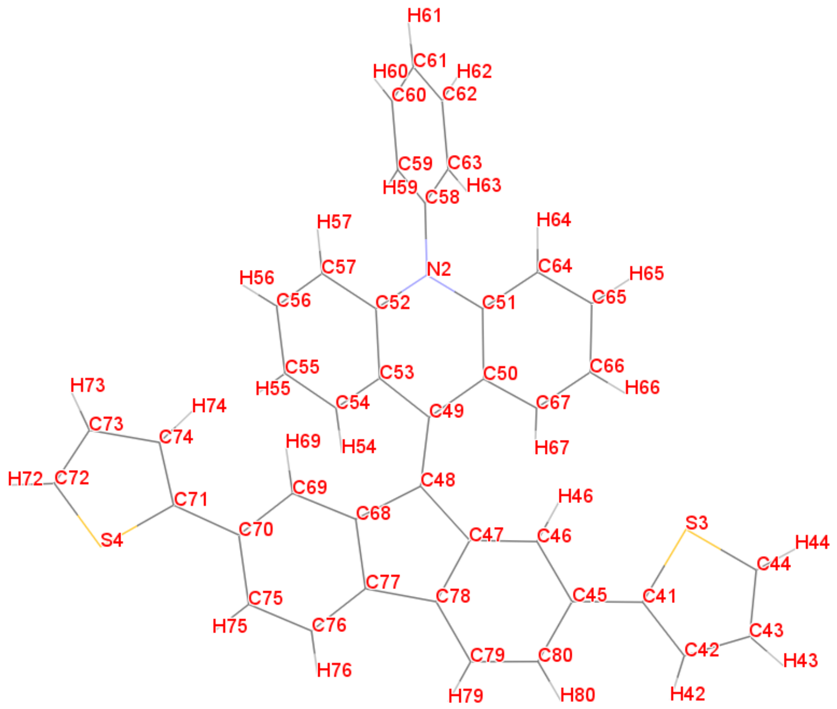

Supplementary Table 20. Bond lengths for (thienyl)$_2$FAPh (**5e**) (twisted).

Atom	Atom	Length/Å	Atom	Atom	Length/Å	Atom	Atom	Length/Å
S001	C069	1.729(6)	C019	C028	1.401(6)	C040	C047	1.448(7)
S001	C089	1.696(8)	C020	C051	1.364(7)	C041	C063	1.391(8)
S002	C067	1.719(6)	C021	C022	1.416(7)	C041	C072	1.386(7)
S002	C088	1.711(8)	C021	C023	1.374(7)	C043	C049	1.390(7)
S003	C040	1.701(6)	C022	C032	1.424(7)	C044	C056	1.472(7)
S003	C082	1.662(7)	C022	C067	1.465(7)	C044	C058	1.485(7)
S004	C066	1.707(6)	C023	C028	1.482(7)	C045	C048	1.395(7)
S004	C087	1.662(8)	C023	C034	1.426(7)	C045	C057	1.433(8)
N005	C012	1.403(6)	C024	C051	1.413(7)	C045	C066	1.474(8)
N005	C017	1.376(6)	C024	C062	1.367(7)	C046	C056	1.386(7)
N005	C041	1.431(6)	C025	C066	1.541(7)	C047	C049	1.388(7)
C006	N007	1.370(6)	C025	C084	1.544(9)	C047	C070	1.429(7)
C006	C018	1.409(6)	C026	C054	1.374(7)	C048	C058	1.403(7)
C006	C062	1.414(7)	C027	C055	1.406(7)	C050	C079	1.369(8)
N007	C015	1.395(6)	C028	C043	1.476(7)	C052	C070	1.375(7)
N007	C037	1.446(6)	C029	C034	1.457(7)	C056	C061	1.422(7)
C008	C009	1.438(6)	C029	C043	1.403(7)	C057	C078	1.382(8)
C008	C015	1.421(6)	C029	C052	1.398(7)	C059	C068	1.400(8)
C008	C026	1.421(7)	C030	C055	1.373(7)	C061	C064	1.373(7)

C009	C018	1.462(7)	C031	C046	1.399(7)	C063	C077	1.386(8)
C009	C044	1.410(7)	C031	C069	1.477(8)	C064	C076	1.401(8)
C010	C016	1.416(7)	C031	C076	1.405(8)	C065	C067	1.446(8)
C010	C017	1.411(7)	C032	C053	1.362(7)	C065	C080	1.463(9)
C010	C019	1.461(6)	C033	C042	1.412(7)	C068	C071	1.363(8)
C011	C015	1.416(6)	C033	C060	1.363(7)	C069	C081	1.370(10)
C011	C038	1.378(7)	C034	C053	1.398(7)	C071	C079	1.395(8)
C012	C013	1.412(6)	C035	C042	1.378(7)	C072	C073	1.373(8)
C012	C035	1.413(7)	C036	C058	1.415(7)	C074	N075	1.097(8)
C013	C019	1.466(7)	C036	C061	1.460(7)	C074	C085	1.432(11)
C013	C060	1.414(7)	C036	C078	1.391(8)	C080	C088	1.345(10)
C014	C040	1.528(7)	C037	C050	1.391(7)	C081	C086	1.411(12)
C014	C083	1.486(8)	C037	C059	1.377(7)	C082	C083	1.379(10)
C016	C027	1.359(7)	C038	C054	1.412(7)	C084	C087	1.326(10)
C017	C030	1.416(7)	C039	C073	1.409(8)	C086	C089	1.357(11)
C018	C020	1.421(7)	C039	C077	1.366(8)			

Supplementary Table 21. Bond angles for $(thienyl)_2FAPh$ (**5e**) (twisted).

Atom	Atom	Atom	Angle/°	Atom	Atom	Atom	Angle/°	Atom	Atom	Atom	Angle/°
C016	C010	C019	122.5(4)	C042	C035	C012	119.6(5)	C077	C063	C041	119.9(5)
C017	C010	C016	117.7(4)	C058	C036	C061	108.4(4)	C061	C064	C076	118.7(5)
C017	C010	C019	119.7(4)	C078	C036	C058	120.1(5)	C067	C065	C080	108.0(5)
C038	C011	C015	120.7(4)	C078	C036	C061	131.5(5)	C025	C066	S004	114.1(4)
N005	C012	C013	119.3(4)	C050	C037	N007	118.3(4)	C045	C066	S004	121.7(4)
N005	C012	C035	120.0(4)	C059	C037	N007	120.0(4)	C045	C066	C025	124.2(5)
C013	C012	C035	120.6(4)	C059	C037	C050	121.5(5)	C022	C067	S002	121.0(4)
C012	C013	C019	121.2(4)	C011	C038	C054	119.4(5)	C065	C067	S002	112.3(4)
C060	C013	C012	116.8(4)	C077	C039	C073	120.2(5)	C065	C067	C022	126.7(5)
C060	C013	C019	121.9(4)	C014	C040	S003	112.7(4)	C071	C068	C059	120.1(5)
C083	C014	C040	104.4(4)	C047	C040	S003	123.3(4)	C031	C069	S001	120.9(4)
N007	C015	C008	120.7(4)	C047	C040	C014	124.1(4)	C081	C069	S001	109.9(5)
N007	C015	C011	118.5(4)	C063	C041	N005	119.9(5)	C081	C069	C031	129.2(6)
C011	C015	C008	120.7(4)	C072	C041	N005	119.7(4)	C052	C070	C047	120.3(5)
C027	C016	C010	121.7(5)	C072	C041	C063	120.3(5)	C068	C071	C079	120.5(6)
N005	C017	C010	121.9(4)	C035	C042	C033	119.9(5)	C073	C072	C041	119.7(5)
N005	C017	C030	118.6(4)	C029	C043	C028	109.7(4)	C072	C073	C039	119.9(5)
C010	C017	C030	119.4(4)	C049	C043	C028	130.9(4)	N075	C074	C085	176.8(8)
C006	C018	C009	121.0(4)	C049	C043	C029	119.0(4)	C064	C076	C031	120.7(5)
C006	C018	C020	118.1(4)	C009	C044	C056	130.7(4)	C039	C077	C063	120.0(5)
C020	C018	C009	120.8(4)	C009	C044	C058	123.8(4)	C057	C078	C036	120.4(5)
C010	C019	C013	115.1(4)	C056	C044	C058	105.6(4)	C050	C079	C071	120.2(5)
C028	C019	C010	126.1(4)	C048	C045	C057	120.0(5)	C088	C080	C065	114.5(6)
C028	C019	C013	118.8(4)	C048	C045	C066	120.3(5)	C069	C081	C086	112.3(7)
C051	C020	C018	121.8(5)	C057	C045	C066	119.6(5)	C083	C082	S003	114.0(5)
C023	C021	C022	121.4(5)	C056	C046	C031	120.5(5)	C082	C083	C014	115.0(6)
C021	C022	C067	120.8(5)	C049	C047	C040	119.7(4)	C087	C084	C025	117.0(6)

C032	C022	C021	117.6(5)	C049	C047	C070	119.3(5)	C089	C086	C081	114.2(8)
C032	C022	C067	121.5(5)	C070	C047	C040	121.0(5)	C084	C087	S004	114.7(6)
C021	C023	C028	131.9(5)	C045	C048	C058	119.5(5)	C080	C088	S002	113.0(6)
C021	C023	C034	119.2(5)	C043	C049	C047	120.7(5)	C086	C089	S001	110.3(7)
C034	C023	C028	107.9(4)	C079	C050	C037	118.9(5)	C089	S001	C069	93.2(3)
C062	C024	C051	120.4(5)	C020	C051	C024	119.1(5)	C088	S002	C067	92.2(3)
C084	C025	C066	100.7(4)	C070	C052	C029	119.3(5)	C082	S003	C040	93.9(3)
C054	C026	C008	122.5(4)	C032	C053	C034	120.3(5)	C087	S004	C066	93.5(3)
C016	C027	C055	120.2(5)	C026	C054	C038	120.1(5)	C012	N005	C041	118.1(4)
C019	C028	C023	130.2(4)	C030	C055	C027	119.5(5)	C017	N005	C012	120.1(4)
C019	C028	C043	124.3(4)	C046	C056	C044	132.0(5)	C017	N005	C041	121.0(4)
C043	C028	C023	105.5(4)	C046	C056	C061	118.5(4)	N007	C006	C018	120.3(4)
C043	C029	C034	108.0(4)	C061	C056	C044	108.8(4)	N007	C006	C062	121.0(4)
C052	C029	C034	130.9(5)	C078	C057	C045	119.8(5)	C018	C006	C062	118.7(4)
C052	C029	C043	121.1(5)	C036	C058	C044	108.8(4)	C006	N007	C015	120.4(4)
C055	C030	C017	120.8(5)	C048	C058	C036	120.0(5)	C006	N007	C037	119.9(4)
C046	C031	C069	121.8(5)	C048	C058	C044	130.4(4)	C015	N007	C037	119.4(4)
C046	C031	C076	119.4(5)	C037	C059	C068	118.6(5)	C015	C008	C009	120.2(4)
C076	C031	C069	118.8(5)	C033	C060	C013	121.9(5)	C026	C008	C009	122.9(4)
C053	C032	C022	121.4(5)	C056	C061	C036	108.4(4)	C026	C008	C015	116.4(4)
C060	C033	C042	119.9(5)	C064	C061	C036	129.8(5)	C008	C009	C018	115.4(4)
C023	C034	C029	108.9(4)	C064	C061	C056	121.8(5)	C044	C009	C008	126.6(4)
C053	C034	C023	119.7(5)	C024	C062	C006	121.0(5)	C044	C009	C018	117.8(4)
C053	C034	C029	131.2(5)								

8. Various Chromic Behavior

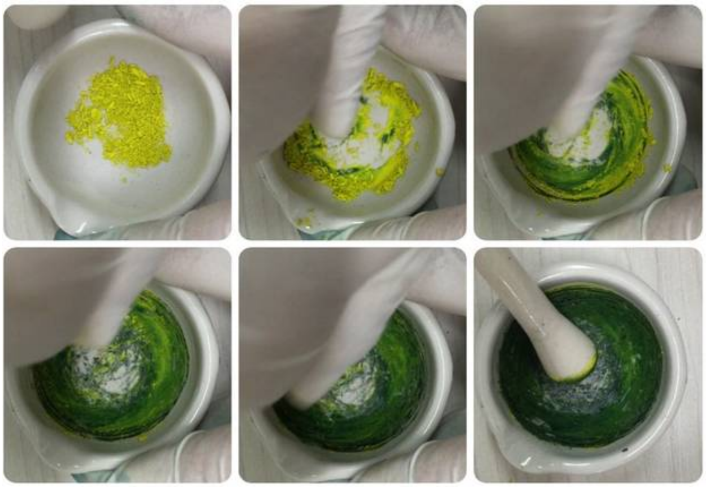

Supplementary Figure 63. Mechanical chromism of $(OMe)_2$FAPh (**5c**).

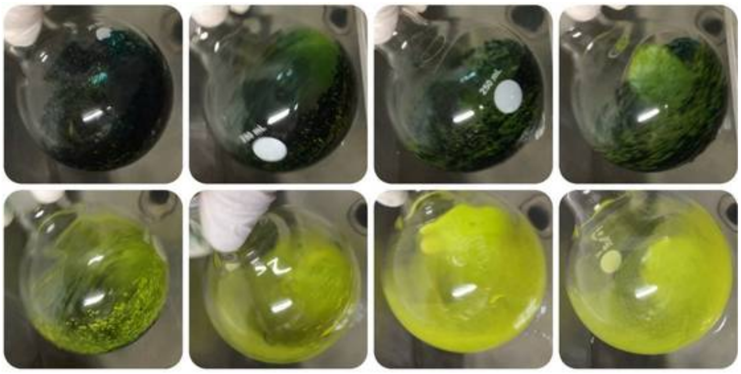

Supplementary Figure 64. Ultra sound induced chromism of $(OMe)_2$FAPh. Solid of compound **5c** formed in the round-bottom flask after evaporation of dichloromethane, followed by addition of methanol, in which **5c** almost have no solubility. This treatment immediately gave a suspension of a yellow powder of **5c**. The recovered powder was checked by NMR to confirm that **5c** had not decomposed. This was related to the crystallization of **5c** into the folded crystals.

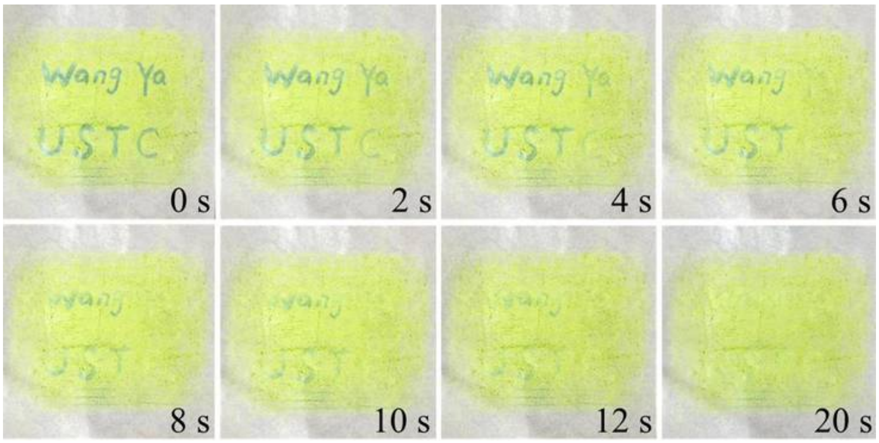

Supplementary Figure 65. Thermal induced chromism of **5e**. Heat a paper with folded **5e** powder (with ground **5e** draws) in a heating plate, color of the drawing changed from green to yellow gradually after 100 s (In other words, drawing disappeared). Working temperature is around 70 °C.

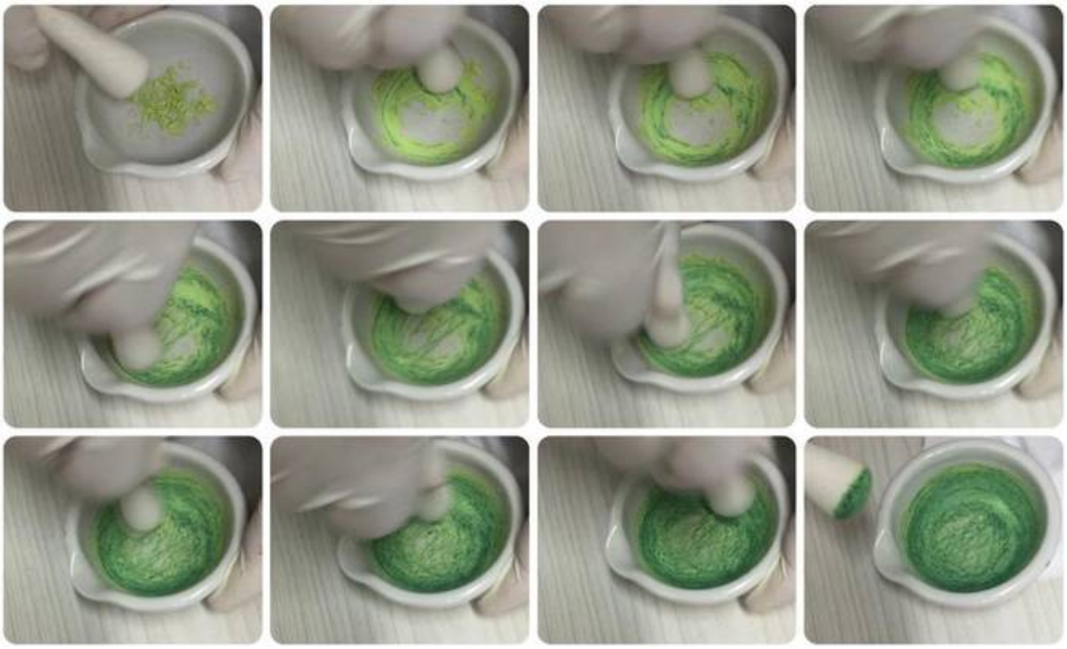

Supplementary Figure 66. Mechanical chromism of (thienly)$_2$FAPh (**5e**).

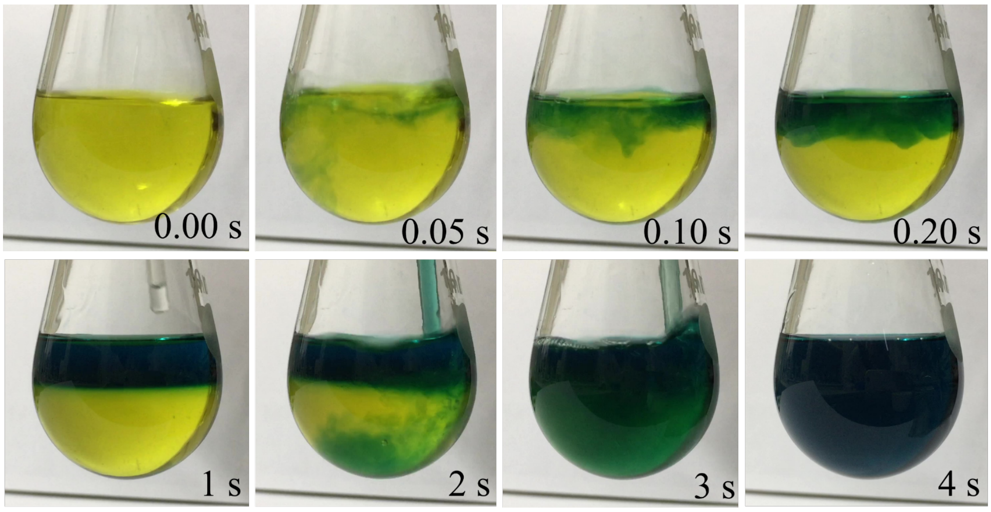

Supplementary Figure 67. Reversible protonation of **5e**. After additional of 10 equiv of glacial acetic acid, **5e** solution got yellow. That process could be reversed after additional of 10 equiv of organic base TEA.

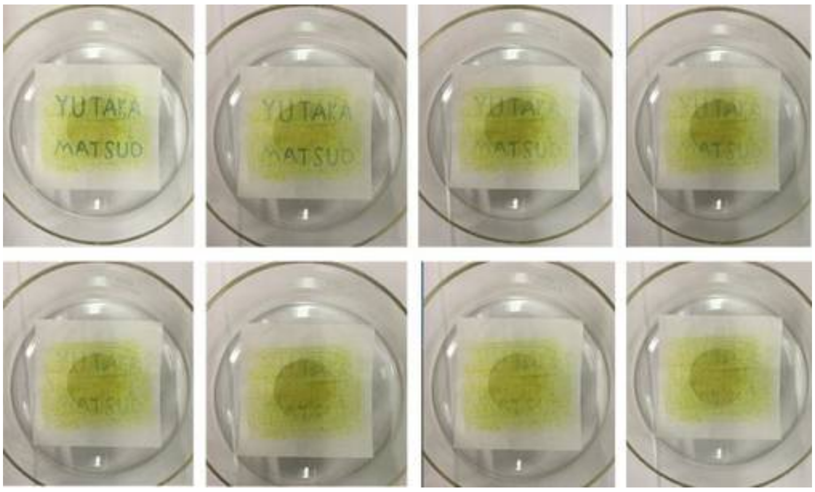

Supplementary Figure 68. Vapor-induced chromism of **5b** in DCM vapor in r.t..

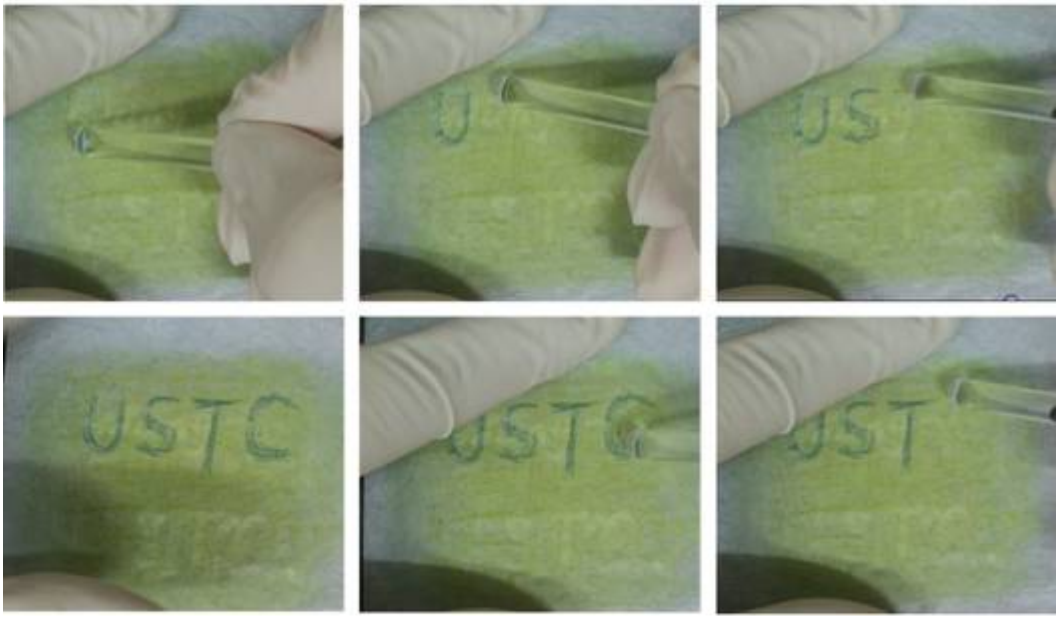

Supplementary Figure 69. Mechanical chromism of **5b**.

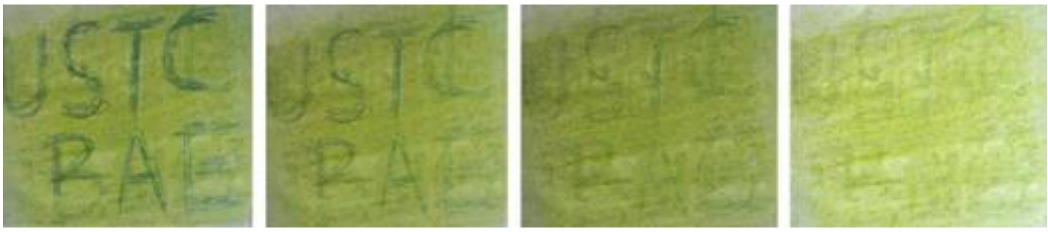

Supplementary Figure 70. Thermal-induced chromism of **5b**. Heat a paper with folded **5b** powder (with ground **5b** draws) in a heating plate, color of the drawing changed from green to yellow gradually after 100 s (In other words, drawing disappeared). Working temperature is around 70 ^{o}C.

9. Cyclic Voltammograms

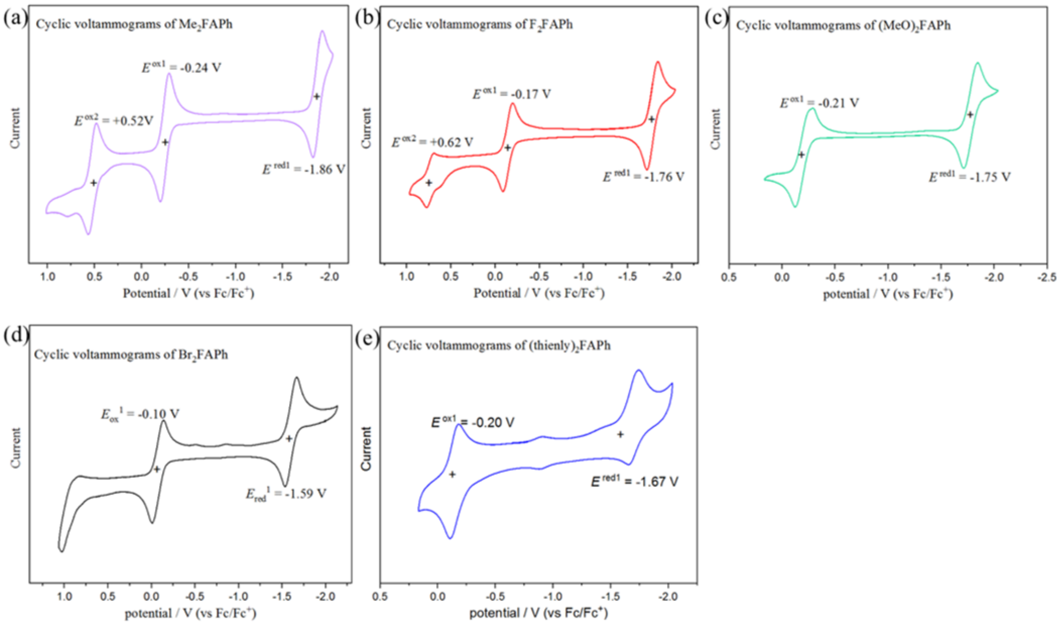

Supplementary Figure 71. Cyclic voltammograms of selected compound in dichloromethane containing *n*-$Bu_4N^+PF_6^-$ as a supporting electrolyte. Scan rate = 100 mV/s. Pt working and counter electrodes were used. The reference electrode is Ag/Ag^+. (a) **5a**, (b) **5b**, (c) **5c**, (d) **5d**, and (e) **5e**, respectively.

10. DFT Studies

Supplementary Note 4

Free energy differences between the two conformers of **5a** and those of **5e** were calculated to be 2.8 and 5.7 kJ·mol^{-1}, respectively, indicating the twisted conformers were then thermodynamically slightly stable than the folded conformers on the B3LYP calculation. This is because the M06 method can estimate weak intermolecular interaction with evaluating non-covalent bonds. We considered, however, the energy differences of conformers were still small, confirming both conformers have almost the same energy. According to the free energy diagram (Supplementary Figure 72a), the relative rotation of the two tricyclic aromatic units about the C9=C9' bond for conversion between the anti-folded and twisted conformation is the major isomerization process. For **5e**, the energy barrier between local minima with an anti-folded conformation and global minima with a twisted conformation was 26.1 kJ·mol^{-1}, which was low, indicating that interconversion between these two conformers can easily occur. The energy barrier for conversion between the two global minima with twisted conformers was 42.8 kJ·mol^{-1}.

Cartesian coordinates are included in Supplementray Data 1–4. For **5e** twisted, total electronic energy = –2390.9874191, thermal corrections to Gibbs free energy = 0.459899. For **5e** folded, total electronic energy = –2390.9843144, thermal corrections to Gibbs free energy = 0.458967. For TS1, total electronic energy = –2390.9776912, thermal corrections to Gibbs free energy = 0.460091. For TS2, total electronic energy = –2390.968902, thermal corrections to Gibbs free energy = 0.457663.

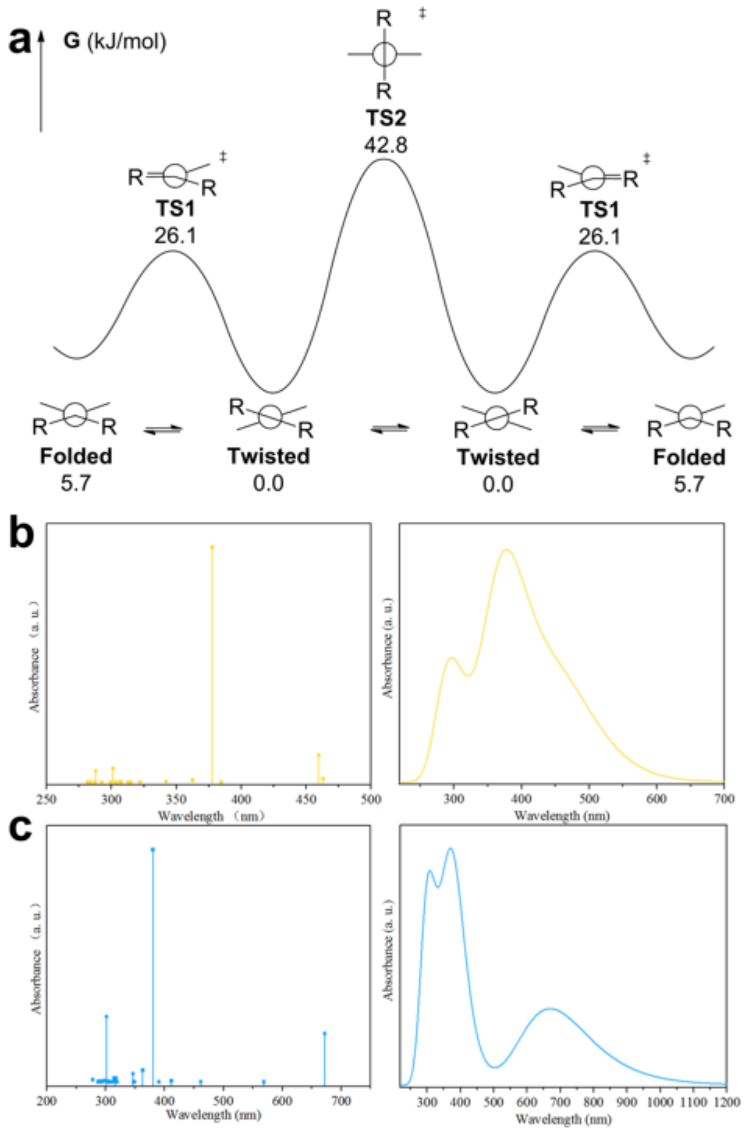

Supplementary Figure 72. Computational calculation for conformational isomerization and UV-Vis spectra of **5e**. (a) The energy diagram in conformational isomerism, (b) Calculated absorption for the folded **5e** with an oscillator strength higher than 0.1 and an absorption spectrum fitting. Peaks: 463 nm, 459 nm, 377 nm, and 301 nm. Calculated absorption for the twisted **5e** with an oscillator strength higher than 0.1 and an absorption spectrum fitting. Peaks: 671 nm, 381 nm, and 301 nm. Among them, 671 nm absorption can be attributed to HOMO->LUMO transition.

Molecular orbitals

(a) **5a**

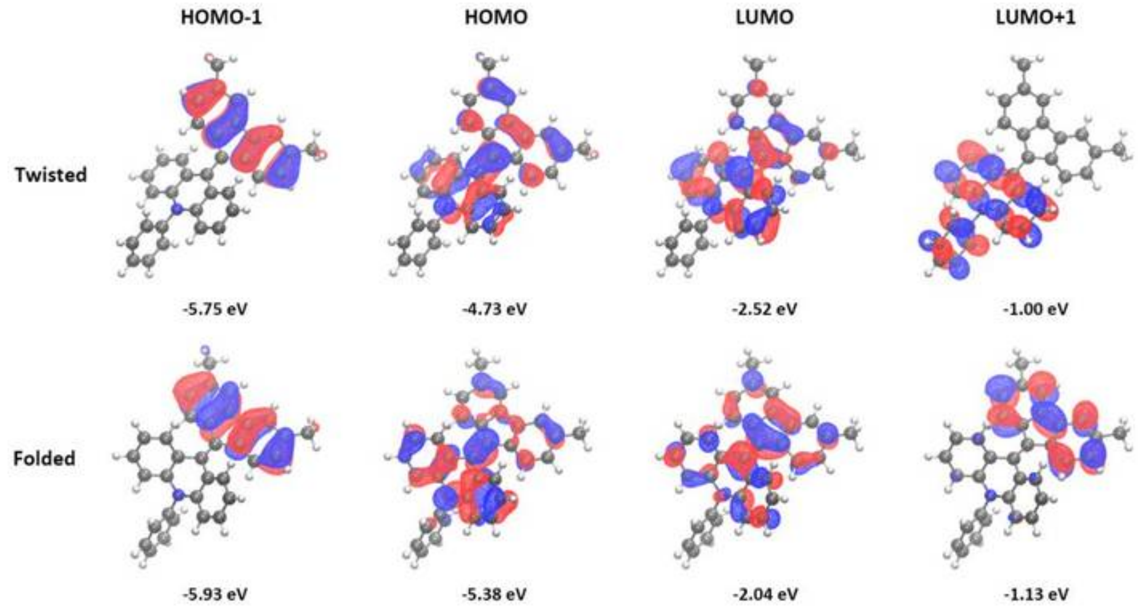

(b) **5b**

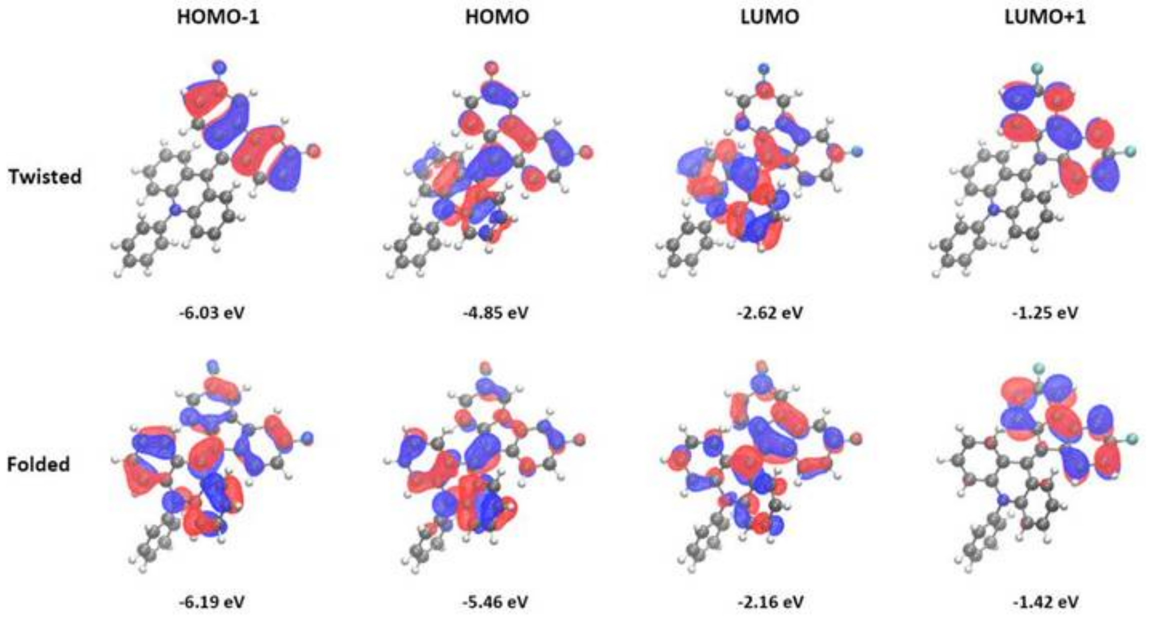

(c) **5c**

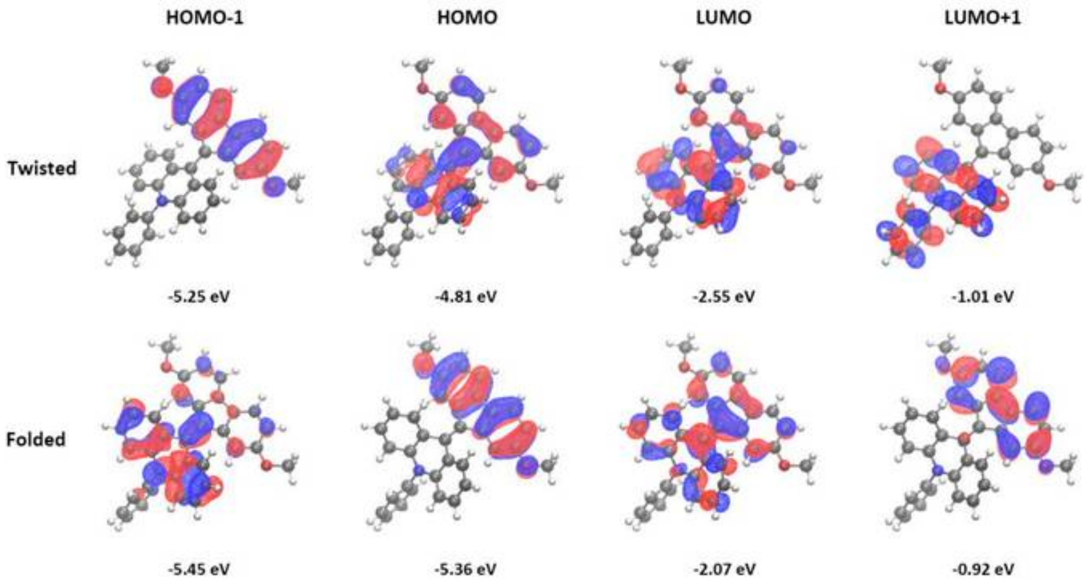
HOMO-1
HOMO
LUMO
LUMO+1
Twisted
-5.25 eV
-4.81 eV
-2.55 eV
-1.01 eV
Folded
-5.45 eV
-5.36 eV
-2.07 eV
-0.92 eV

(d) **5d**

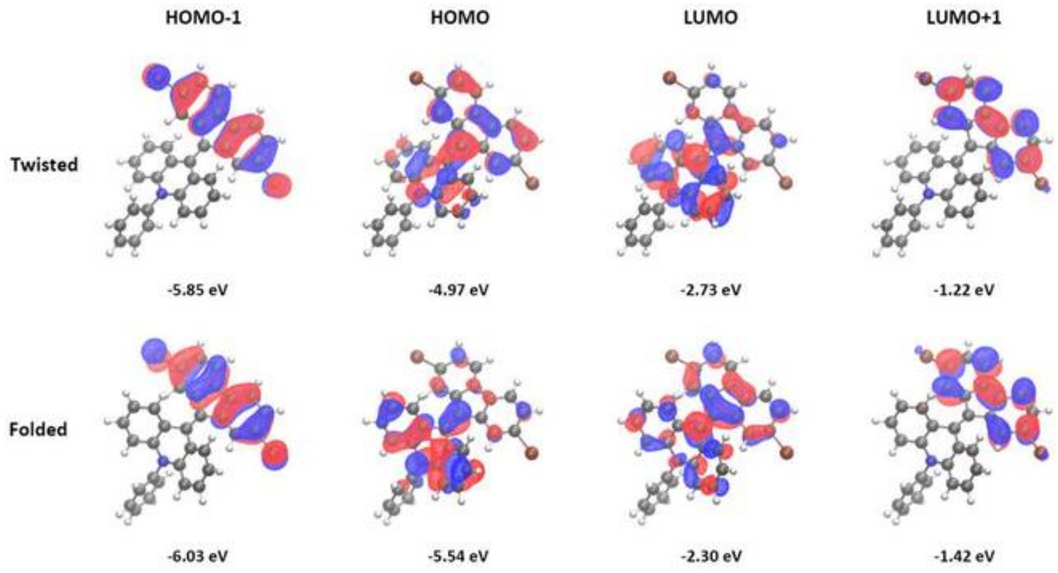
HOMO-1
HOMO
LUMO
LUMO+1
Twisted
-5.85 eV
-4.97 eV
-2.73 eV
-1.22 eV
Folded
-6.03 eV
-5.54 eV
-2.30 eV
-1.42 eV

(e) **5e**

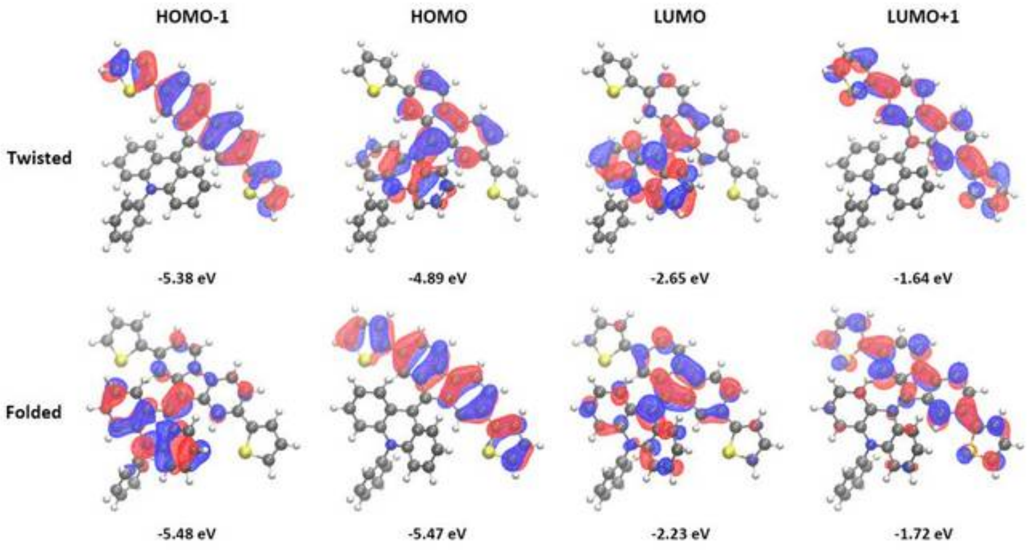
HOMO-1
HOMO
LUMO
LUMO+1
Twisted
-5.38 eV
-4.89 eV
-2.65 eV
-1.64 eV
Folded
-5.48 eV
-5.47 eV
-2.23 eV
-1.72 eV

(f) **5f**

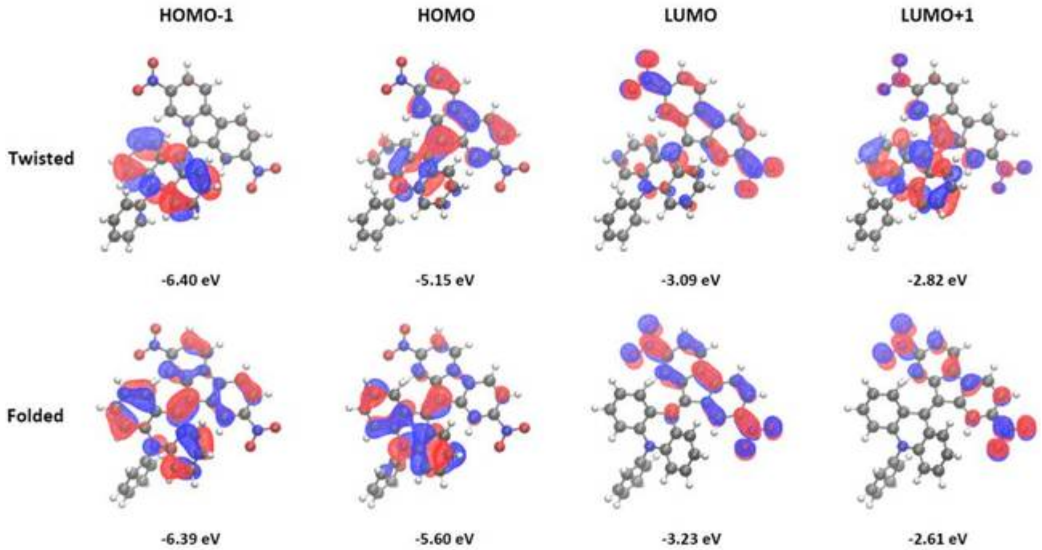

Supplementary Figure 73. Molecular orbitals, LUMO+1, LUMO, HOMO and HOMO-1 of (a) **5a**, (b) **5b**, (c) **5c**, (d) **5d** ,(e) **5e**, and (f) **5f**.

Supplementary Table 22. Energy of the species in eV.

	energy levels (eV)				
	HOMO-1	HOMO	LUMO	LUMO+1	E_g[a]
**5a** folded	-5.93	-5.38	-2.04	-1.13	3.34
**5a** twisted	-5.75	-4.73	-2.52	-1.00	2.21
**5b** folded	-6.19	-5.46	-2.16	-1.42	3.3
**5b** twisted	-6.03	-4.85	-2.62	-1.25	2.23
**5c** folded	-5.45	-5.36	-2.07	-0.92	3.29
**5c** twisted	-5.25	-4.81	-2.55	-1.01	2.26
**5d** folded	-6.03	-5.54	-2.30	-1.42	3.24
**5d** twisted	-5.85	-4.97	-2.73	-1.22	2.24
**5e** folded	-5.48	-5.47	-2.23	-1.72	3.24
**5e** twisted	-5.38	-4.89	-2.65	-1.64	2.24
**5f** folded	-6.39	-5.60	-3.23	-2.61	2.37
**5f** twisted	-6.40	-5.15	-3.09	-2.82	2.06

[a] $E_{LUMO} - E_{HOMO}$. E_{LUMO} and E_{HOMO} are calculated energy levels for the LUMO and HOMO, respectively.

Supplementary Note 5

We calculated the distribution of MOs, HOMO–1, HOMO, LUMO, and LUMO+1 (Supplementary Figure 73), energy levels, and bandgaps (E_g = HOMO–LUMO) for both folded and twisted conformers (Supplementary Table 22) at the B3LYP method. In analyzing the distributions of MOs, spatial separation of the HOMO and LUMO was not clearly seen except in a few cases. The folded conformers of **5c** and **5e** were two exceptions, with HOMOs in the fluorene group because of the electron-donating methoxy and thienyl groups, respectively. Another exception was **5d**, for which both the folded and twisted forms had LUMOs primarily in the fluorene moiety due to the strongly electron-withdrawing nitro groups. We confirmed relatively large effect of direct substitution in FAs on MO distribution change. In addition, from the MO distribution, we concluded that the HOMO–LUMO transition is attributable to both intramolecular charge transfer and the π-π* transition. As Supplementtray Table 23 shows, compared with the folded conformers, the

twisted conformers have smaller bandgaps, with higher-lying HOMO levels from –5.54 to –4.73 eV and lower-lying LUMO levels from –2.04 to –2.73 eV. The bandgap E_g of the twisted **5f** was the smallest, with the deepest HOMO and LUMO levels among the six compounds. In **5f**, the LUMO was distributed on the nitro group itself, which is potentially problematic in charge carrier transporting devices.

11. Charge Carrier Mobility Data

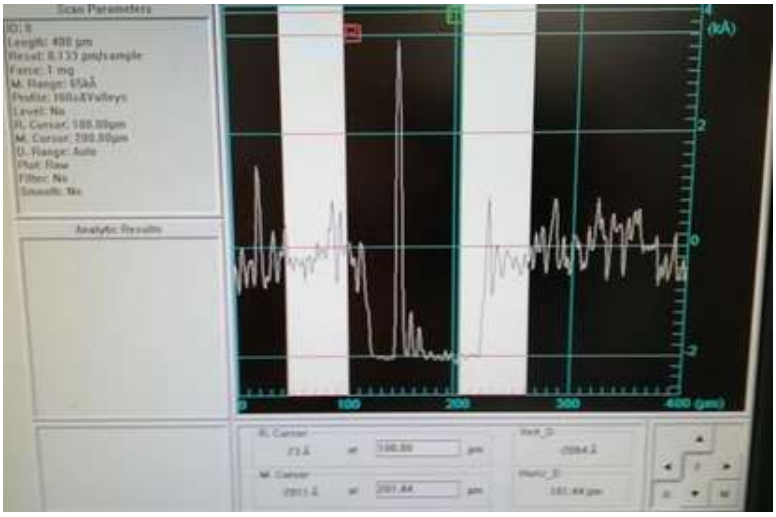

Supplementary Figure 74. Measurement window of SCLC file devices for selective sample **5b**.

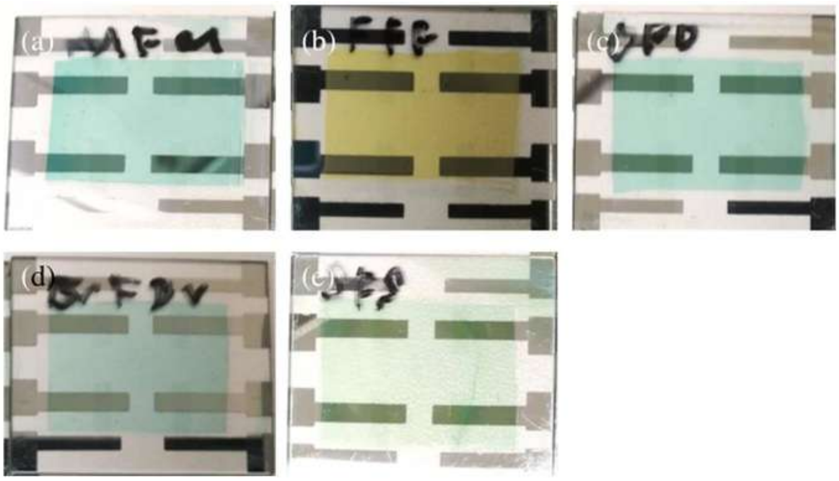

Supplementary Figure 75. Devices for SCLC measurement of **5a**, **5b** ,**5c**, **5d**, and **5e**.

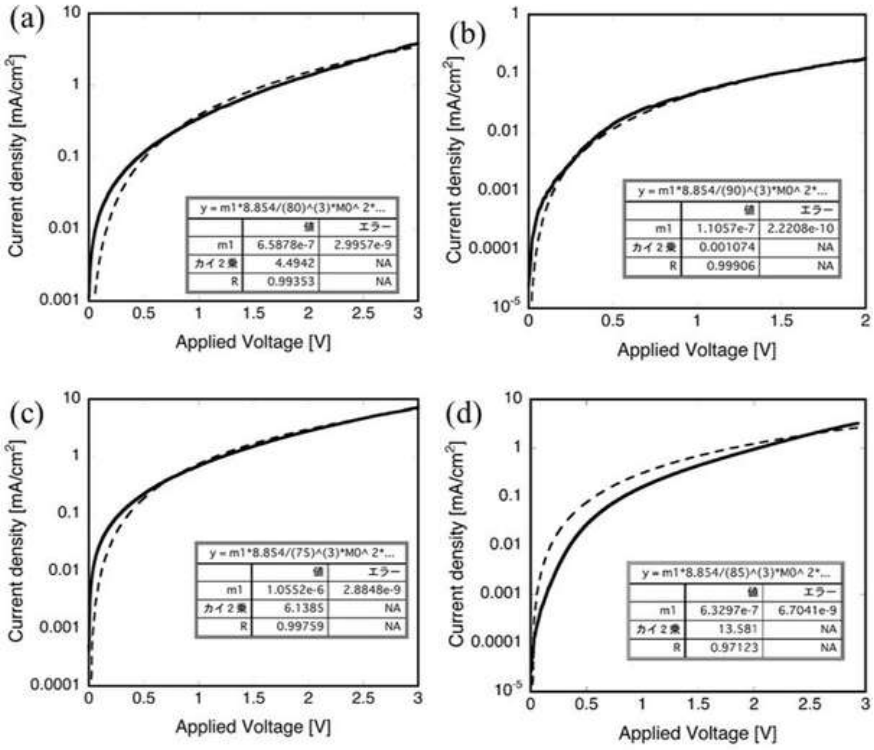

Supplementary Figure 76. SCLC measurement for hole mobility of selected compound. Solid and dashed lines are measurement and fitted data, respectively. (a) **5a**, (b) **5c**, (c) **5d**, and (d) **5e**, respectively. Device condition: ITO/PEDOT: PSS/active layer/MoO_3/Al. Concentration: 10 mg/mL in CH_2Cl_2. Spin ratio: 2000 rpm. Results: **5a**: 6.6×10^{-7} $cm^2·V^{-1}·s^{-1}$ (thickness 80nm); **5c**: 1.1×10^{-7} $cm^2·V^{-1}·s^{-1}$ (thickness 90nm); **5d**: 1.1×10^{-6} $cm^2·V^{-1}·s^{-1}$ (thickness 75nm); **5e**: 6.3×10^{-7} $cm^2·V^{-1}·s^{-1}$ (thickness 85nm).

12. Exaplanation of Equilibrium Study Based on Variable Temperature UV-Vis

Supplementary Table 23. Variable temperature UV-Vis data and themraldynamic calculation. ΔH = 8.314 × 506/1000 kJ/mol = 4.21 kJ/mol; ΔS = 8.314 × 3.0325/1000 = 0.025.2 kJ/mol; ΔG = 4.21 kJ/mol – 0.0252 × 303 kJ/mol = –3.43 kJ/mol. If we use the value of ΔG (-3.43 kJ/mol) we got above, we will get K = 3.8955, almost a same value as we calculated (3.86).

Temperature/°C	$\varepsilon_{folded}/\varepsilon_{twisted}$	A_{folded}	$A_{twisted}$	$c_{folded}/c_{twisted}$	ln$K$	T	1/T
30	1/6.84	0.423	0.693	3.862	1.351	303	0.00333
40	1/6.84	0.431	0.666	4.096	1.410	313	0.00320
50	1/6.84	0.434	0.634	4.328	1.465	323	0.00310
60	1/6.84	0.438	0.614	4.509	1.506	333	0.00300
70	1/6.84	0.442	0.588	4.759	1.560	343	0.00292
80	1/6.84	0.442	0.566	4.941	1.602	353	0.00283

k	b	ΔH (kJ/mol)	ΔS (kJ/mol)	ΔG (kJ/mol)
-506.05	3.03255	4.207	0.0252	-3.426

Note：Fitting from $y = k \cdot x + b = \ln K$; $\Delta H = R \cdot k$; $\Delta S = R \cdot b$.

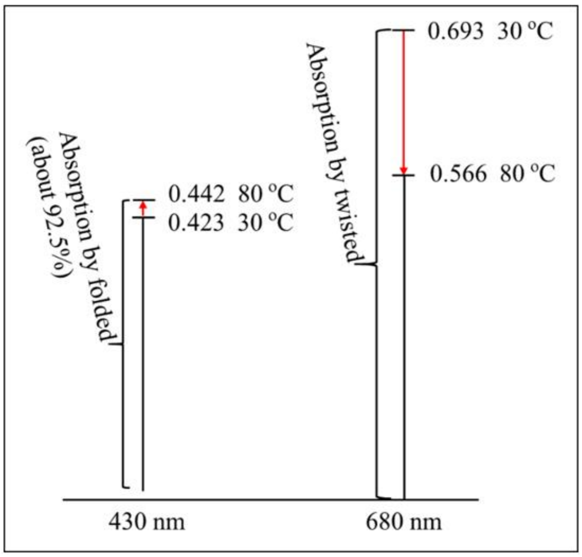

Supplementary Figure 77. Calculation explanation.

$$A = \begin{pmatrix} x_1^2 & x_1 & 1 \\ \vdots & \vdots & \vdots \\ x_n^2 & x_n & 1 \end{pmatrix} \qquad Y = \begin{pmatrix} \ln y_1 \\ \vdots \\ \ln y_n \end{pmatrix} \qquad X = \begin{pmatrix} a \\ b \\ c \end{pmatrix}$$

Supplementary Figure 78. Matrix for Gaussian fitting.

Supplementary Note 6

By simultaneous formulas, in 30 °C, the ratio of $\varepsilon_{folded}/\varepsilon_{twisted}$ = (0.693 – 0.566)/(0.442 – 0.423), was calculated to be 1/6.84, thus the ratio of $c_{folded}/c_{twisted}$ = (0.423/0.693) × 6.84 × 0.925 = 3.86. Two basis: (1) L-B law works well in that concentration range; (2) K is almost a constant value in that temperature range. Two Gaussian-shaped line at 650 nm and 430 nm were simulated as y = e^(–0.9085 × (x/100)^2 + 12.2173 × x/100

– 41.4515) and $y = e\hat{}(-7.8904 \times (x/100)^2 + 67.8576 \times x/100 - 146.8101)$. Absorbance were fitted by the Gauss formula $y = e\hat{}(a(x\hat{}2)+b\cdot x+c)$, which is equal to $\ln y = a\cdot x^2 + b\cdot x + c$. In this regard, the coefficient of Gauss formula can be calculated by matrix. Input matrix above (Supplementary Figure 78), Since $Y = A\cdot x$, we can get $X = (A^{T}\cdot A)^{-1}\cdot A^{T}\cdot Y$. The overlapped peaks were simulated by MATLAB 2019b of the following code: *x*=inv (*A*.'**A*) **A*.'**Y*

13. Melting and Protonation/Deprotonation

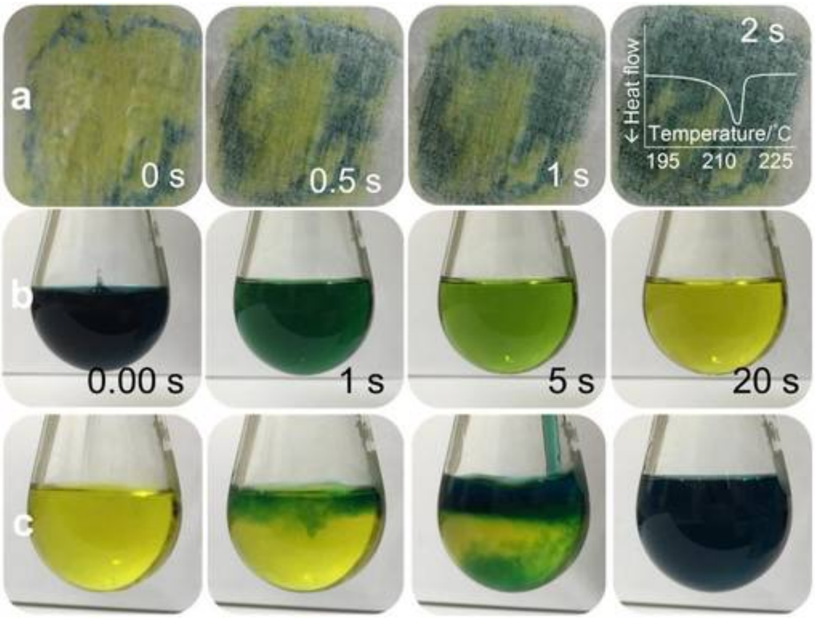

Supplementary Figure 79. (a) Melting of **5a**, (b) Protonation of **5a** by HOAc. That is reversible by addition of TEA, (c) Deprotonation by TEA.

14. Piezofluorochromism

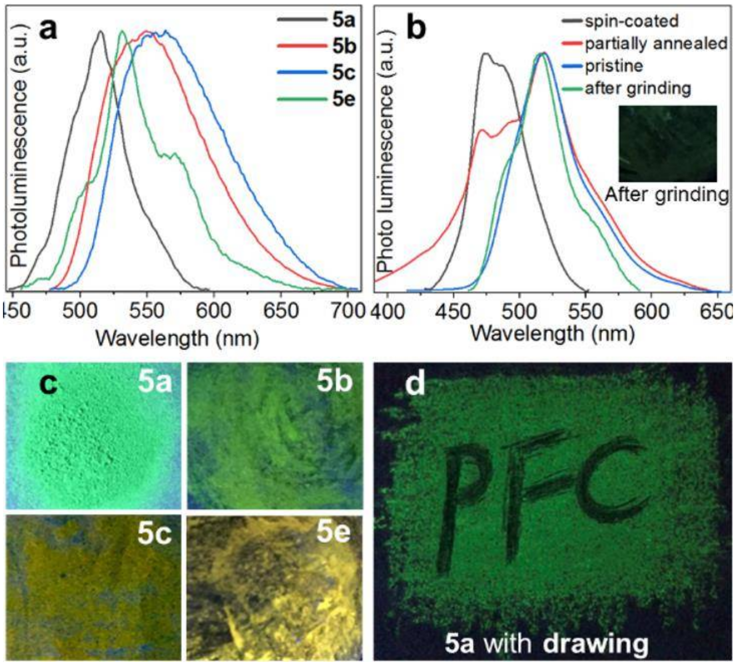

Supplementary Figure 80. Photoluminescence properties. a) Normalized photoluminescence spectra of **5a** (folded), **5b** (folded), **5c** (folded), and **5e** (folded) on glass substrates under 365 nm excitation. No photoluminescence emission peak was observed for any twisted conformers, b) Normalized photoluminescence spectra of **5a** spin-coated (black line), partially thermal annealed at 80 °C (red line), annealed (called the pristine state; blue line), and after grinding on a glass substrate (green line; intensity markedly decreased). Inset: **5a** after grinding under 365 nm excitation, c) Photographs of photoluminescence for **5a** (powder), **5b**, **5c**, and **5e** (absorbed in paper) under 365 nm excitation, d) Photograph of paper with absorbed **5a** after drawing under 365 nm excitation.

15. Powder XRD

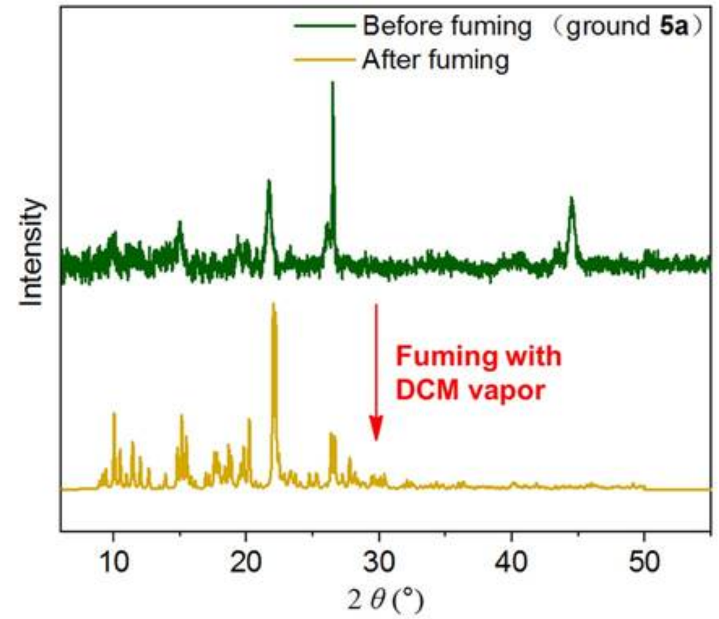

Supplementary Figure 81. Powder XRD measurements of **5a** before and after fuming with DCM vapor. After grinding yellow **5a**, ground **5a** was obtained. Powder XRD measurements of ground **5a** (before fuming) and **5a** after fuming with DCM vapor were carried out. By comparison, the peak around 44.48° and a shark peak around 26.49°(2*θ*) disappeared after fuming with DCM vapor. There is a slight shift towards high diffraction angle for the peak around 21.73°. After fuming, many diffraction peak appear sharp, indicating the process of micro crystallization.

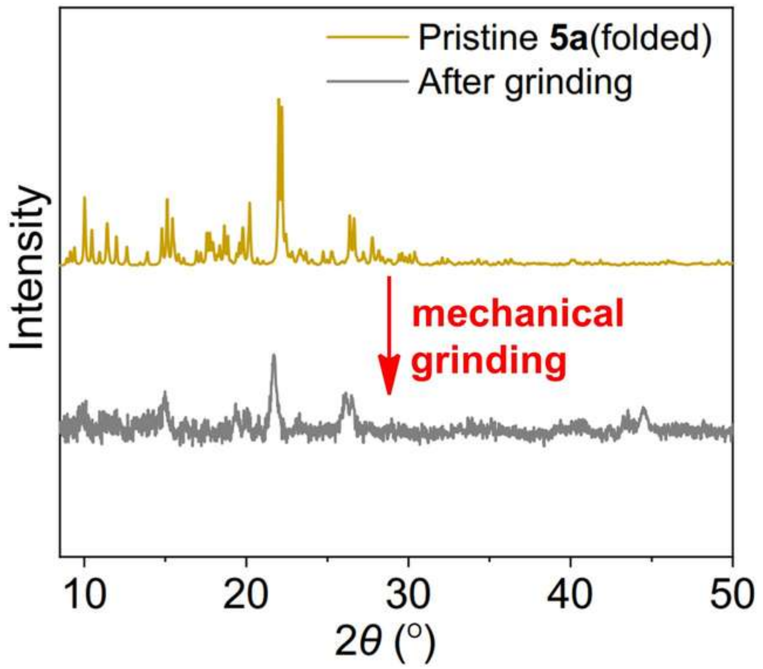

Supplementary Figure 82. Powder XRD measurements of **5a** before and after grinding upon pristine folded state. There are obvious differences between the XRD patterns of **5a** before and after grinding upon pristine folded state. Several diffraction peaks of **5a** powder degenerated, indicating the formation of an almost amorphous compound by morphological transition.

16. Supplementary References

1. D. Liao, H. Li, X. Lei, *Org. Lett.*, **2012**, *14*, 18
2. T. I. KirichenkoS. B. MeshkovaZ. M. TopilovaA. V. KiriyakA. Yu. LyapunovE. Yu. KulyginaN. G. Luk'yanenko, *Russian Journal of General Chemistry*, **2005**, *75(2)*, 272277
3. Z. D. Liu, Y. Z. Chang, C. J. Ou, J. Y. Lin, L.H. Xie, *Polym. Chem.*, **2011**, *2*, 2179
4. X. Zhang, J.B. Han, P.F. Li, X. Ji, *Synthetic Communications*, **2009**, *39*, 3804–3815.
5. M. Z. Asfari, V. Böhmer, J. Harrowfield, *Bull. Chem. Soc. Jpn.* **1996**, *69*, 3633–3638.
6. K. Okano; S. Ogino; M. Kawamoto; T. Yamashita, *Chem. Commun.* **2011**, *47*, 11891–11893.
7. T. Suzuki; H. Okada,; T. Nakagawa,; K. Komatsu; C. Fujimoto; H. Kagi; Y. Matsuo, *Chem. Sci.* **2018**, *9*, 475–482.
8. J. Luo, K. Song, F. L. Gu, Q. Miao, *Chem. Sci.*, **2011**, *2*, 2029.
9. Gaussian 16, Revision A.01, J. W. Ochterski, R. L. Martin, K. Morokuma, O. Farkas, J. B. Foresman, D. J. Fox, Gaussian, Inc., Wallingford CT, **2016**.
10. A. D. Becke, *Phys. Rev. A*, **1988**, *38*, 3098;
11. C. Lee, W. Yang, R. G. Parr, *Phys. Rev. B*, **1988**, *37*, 785;
12. A. D. Becke, *J. Chem. Phys.* **1993**, *98*, 1372.
13. S. Grimme, J. Anthony, S. Ehrlich, H. Krieg, *J. Chem. Phys.* **2010**, *132*, 154104.
14. A. V. Marenich, C. J. Cramer, D. G. Truhlar, *J. Phys. Chem. B* **2009**, *113*, 6378–6396.
