# Supplementary material for: Equilibrium and thermodynamic studies of chromic overcrowded fluorenylidene-acridanes with modified fluorene moieties
Source: Commun Chem. 2020 Jul 24;3:93. doi: 10.1038/s42004-020-00345-6 (PMC9814365; doi:10.1038/s42004-020-00345-6)
Supplement: Supplementary file 15 — Description of Additional Supplementary Files [file 42004_2020_345_MOESM15_ESM.pdf]

Description of Additional Supplementary Files

Supplementary Data 1
File Name: Supp Data 1 folded 5e b3lyp.xyz
Description: Coordinates file for the optimized structure of folded **5e**

Supplementary Data 2
File Name: Supp Data 2 twisted 5e b3lyp.xyz
Description: Coordinates file for the optimized structure of twisted **5e**

Supplementary Data 3
File Name: Supp Data 3 TS1 of 5e b3lyp.xyz
Description: Coordinates file for the transition state 1 (TS1) of **5e**

Supplementary Data 4
File Name: Supp Data 4 TS2 of 5e b3lyp.xyz
Description: Coordinates file for the transition state 2 (TS2) of **5e**

Supplementary Data 5
File Name: Supp Data 5 folded 5a.cif
Description: Crystallographic data file in CIF format for folded **5a**

Supplementary Data 6
File Name: Supp Data 6 folded 5b.cif
Description: Crystallographic data file in CIF format for folded **5b**

Supplementary Data 7
File Name: Supp Data 7 folded 5c.cif
Description: Crystallographic data file in CIF format for folded **5c**

Supplementary Data 8
File Name: Supp Data 8 folded 5e.cif
Description: Crystallographic data file in CIF format for folded **5e**

Supplementary Data 9
File Name: Supp Data 9 twisted 5d.cif
Description: Crystallographic data file in CIF format for twisted **5d**

Supplementary Data 10
File Name: Supp Data 10 twisted 5e.cif
Description: Crystallographic data file in CIF format for twisted **5e**

Supplementary Data 11
File Name: Supp Data 11 twisted and folded 5a.cif
Description: Crystallographic data file in CIF format for twisted and folded **5a**

Supplementary Data 12
File Name: Supp Data 12 MeFMeA(OMe)Ph (6a).cif
Description: Crystallographic data file in CIF format for compound **6a**
